# Supplementary material for: CATALYST trial protocol: a multicentre, open-label, phase II, multiarm trial for an early and accelerated evaluation of the potential treatments for COVID-19 in hospitalised adults
Source: BMJ Open. 2021 Nov 11;11(11):e050202. doi: 10.1136/bmjopen-2021-050202 (PMC8587583; doi:10.1136/bmjopen-2021-050202)
Supplement: Supplementary data [file bmjopen-2021-050202supp001.pdf]

CATALYST

Supplementary Appendix 2 – CATALYST WHO dataset

Data category	Information
Primary registry and trial identifying number	EudraCT Number<br>2020-001684-89
Date of registration in primary registry	15-May-2020
Secondary identifying numbers	ISRCTN: 40580903
Source(s) of monetary or material support	Medical Research Council
Primary sponsor	University of Birmingham
Secondary sponsor(s)	n/a
Contact for public queries	TV: t.v.veenith@bham.ac.uk<br>BF: b.fisher@bham.ac.uk
Contact for scientific queries	TV: t.v.veenith@bham.ac.uk<br>BF: b.fisher@bham.ac.uk
Public title	Which treatment could lessen the severity of a coronavirus infection when compared with usual care in an NHS setting?
Scientific title	A multicentre, open-label, phase II, multi-arm trial for an early and accelerated evaluation of the potential treatments for COVID-19 in hospitalised adults
Countries of recruitment	UK
Health condition(s) or problem(s) studied	COVID-19
Intervention(s)	Usual care provided following the current institutional policy for patients with COVID-19
	Usual care combined with namilumab
	Usual care combined with infliximab

Veenith T, *et al. BMJ Open* 2021; 11:e050202. doi: 10.1136/bmjopen-2021-050202

CATALYST

**Data category**	**Information**
Key inclusion and exclusion criteria	Ages eligible for study: ≥16 years<br>Sexes eligible for study: both<br>Accepts healthy volunteers: no
	Inclusion criteria: adult patient (≥ 16 years), patient hospitalised with SARS-CoV-2 pneumonia
	Exclusion criteria: allergy against namilumab or infliximab, pregnancy or breastfeeding women, tuberculosis or other severe (non-SARS-CoV-2) infections
Study type	Interventional
	Allocation: randomised, open-label
	Primary purpose: safety and biological signal for efficacy
	Phase II
Date of first enrolment	May-2020
Target sample size	Up to 60 per arm
Recruitment status	Closed
Primary outcome(s)	C-reactive protein concentration over time (time frame: 28 days)
Key secondary outcome	WHO Clinical Progression Improvement Scale (time frame: 28 days)

Veenith T, *et al. BMJ Open* 2021; 11:e050202. doi: 10.1136/bmjopen-2021-050202
