# Supplementary material for: CATALYST trial protocol: a multicentre, open-label, phase II, multiarm trial for an early and accelerated evaluation of the potential treatments for COVID-19 in hospitalised adults
Source: BMJ Open. 2021 Nov 11;11(11):e050202. doi: 10.1136/bmjopen-2021-050202 (PMC8587583; doi:10.1136/bmjopen-2021-050202)
Supplement: Supplementary data [file bmjopen-2021-050202supp002.pdf]

Supplementary Appendix 3 – Summary of CATALYST protocol changes

Amendment number	Date of approval	Protocol version number	Type of amendment	Summary of amendment
1	**REC:** 14-May-20	n/a	Substantial Amendment	Addition of Oxford and UCL as sites
2	**MHRA:** 29-May-20 **HRA:** 01-Jun-20	3.0	Substantial Amendment	Addition of two new IMPs: Namilumab and Infliximab.<br>Update SOE, amendments to inclusion/ exclusion criteria.<br>Specifically: New exclusion criteria relating to the addition of the new drugs:<br>1) Known hypersensitivity to drug products or excipients<br>2) Patients with tuberculosis or other severe infections such as (non-COVID-19) sepsis, abscesses, and opportunistic infections requiring treatment<br>3) Patients with moderate or severe heart failure (NYHA class III/IV)
3	**REC:** 10-Jun-20	n/a	Substantial Amendment	Addition of new sites
4	**MHRA:** 08-Jun-20	n/a	Substantial Amendment	IMPD update
5	**MHRA:** 12-Jun-20 **REC:** 12-Jun-20	4.0	Substantial Amendment	Amendment to inclusion criteria. Specifically:<br>Inclusion criterion 1 changed to:<br>'Hospitalised adult (≥16 yrs) patients with a clinical picture strongly suggestive of SARS-CoV-2 pneumonia (confirmed by chest X-ray or CT scan, with or without a positive reverse transcription polymerase chain reaction [RT-PCR] assay)' in order to:<br>• Allow CT imaging as evidence for COVID-19 pneumonia<br>• Allow recruitment of patients with strong clinical suspicion for COVID-19 pneumonia but with negative PCR assay
6	**MHRA:** 19-Jun-20 **REC:** 20-Jun-20	5.0	Substantial Amendment	Non-substantial amendments to Sample Collection Sub-study text.<br>Amendment to exclusion criteria.<br>Specifically:<br>'Concurrent immunosuppression with biological agents or prednisone dose > 20mg'<br>Was changed to<br>'Concurrent immunosuppression with biological agents' in order to allow patients to be recruited on dexamethasone, following the RECOVERY data

Veenith T, *et al. BMJ Open* 2021; 11:e050202. doi: 10.1136/bmjopen-2021-050202

CATALYST

**Amendment number**	**Date of approval**	**Protocol version number**	**Type of amendment**	**Summary of amendment**
7	**MHRA:**<br>12-Oct-20<br>**REC:**<br>12-Oct-20	6.0	Substantial Amendment	Change of Primary and Secondary Outcomes<br>Specifically:<br>Primary outcome changed to CRP (previously a secondary outcome) from the oxygen saturation to fractional inspired oxygen concentration (SpO2/FiO2) ratio, which now becomes a secondary outcome<br>Hospital free days added as a secondary outcome<br>Overall survival listed as a safety measure (previously death included under hospital survival status as a clinical outcome)<br><br>Applicable changes to Inclusion/ Exclusion Criteria<br>Specifically:<br>Inclusion criteria changed from<br>‘Oxygen saturation (SaO2) of ≤94% while breathing ambient air or a ratio of the partial pressure of Oxygen (PaO2) to the fraction of inspired oxygen (FiO2) (PaO2:FiO2) ≤ 300 mg Hg (≤40kPa’), to ‘CRP ≥40’<br>The following exclusion criteria that relate to the unopened Myelotarg arm were removed from general exclusion and made arm specific:<br>• Known veno-occlusive disease<br>• Neutrophil count < 2 x 109/l or White Blood Cell Count < 4.0 x 109/l<br>The following exclusion criteria was removed as it was felt to be unnecessarily hindering recruitment:<br>• Chronic Obstructive Pulmonary Disease (known FEV1 < 50% predicted or ambulatory or long term oxygen therapy<br><br>Inclusion of Abbreviations list and eCRF table<br>Update to Statistical Analysis section<br>• Justification for CRP, operating characteristics and decision rules

Veenith T, *et al. BMJ Open* 2021; 11:e050202. doi: 10.1136/bmjopen-2021-050202
