# Supplementary material for: CATALYST trial protocol: a multicentre, open-label, phase II, multiarm trial for an early and accelerated evaluation of the potential treatments for COVID-19 in hospitalised adults
Source: BMJ Open. 2021 Nov 11;11(11):e050202. doi: 10.1136/bmjopen-2021-050202 (PMC8587583; doi:10.1136/bmjopen-2021-050202)
Supplement: Supplementary data [file bmjopen-2021-050202supp005.pdf]

Appendix 6 – CATALYST schedule of events for intervention arms

Arm 2: Gemtuzumab Ozogamicin (Mylotarg):

	Baseline	Day 1	Day 2	Day 3	Day 4	Day 5 (+24 hrs)	Day 6	Day 7	Day 8	Day 9	Day 10 (+24 hrs)	Day 11	Day 12	Day 13	Day 14	Day 15 – Day 27	Day 28⌂
IMP pre-medications #		x				x					x						
IMP Administration – Mylotarg		x				x					x						
Liver function test π		x				x					x						
Vital signs (heart rate, blood pressure, temperature) ~		x				x					x						

⌂ Information on Serious Adverse Events (SAEs) will be collected until 28 days after the last IMP administration, which may be after this time point.

Dexamethasone (9.9mg), antihistamine (chlorpheniramine 4-8mg PO or 10mg IV), paracetamol (1g PO or IV) to be given one hour prior to administration.

π Liver function test must be obtained (within the last 24hrs) and REVIEWED prior to IMP administration.

~ Vital signs must be monitored during the infusion and the patient observed for 4 hours after the infusion has ended.

Arm 3: Namilumab

	Baseline	Day 1	Day 2	Day 3	Day 4	Day 5	Day 6	Day 7	Day 8	Day 9	Day 10	Day 11	Day 12	Day 13	Day 14	Day 15 – Day 27	Day 28⌂
IMP administration – Namilumab		x															
Vital signs (heart rate, blood pressure, temperature) ~		x															

⌂ Information on Serious Adverse Events (SAEs) will be collected until 28 days after the last IMP administration, which may be after this time point.

~ Vital signs must be monitored during the infusion and the patient observed for 1 hour after the infusion has ended

Arm 4: Infliximab (Remsima)

	Baseline	Day 1	Day 2	Day 3	Day 4	Day 5	Day 6	Day 7	Day 8	Day 9	Day 10	Day 11	Day 12	Day 13	Day 14	Day 15 – Day 27	Day 28⌂
IMP pre-medications #		X															
IMP Administration - Infliximab		X															
Vital signs (heart rate, blood pressure, temperature) ~		X															

⌂ Information on Serious Adverse Events (SAEs) will be collected until 28 days after the last IMP administration, which may be after this time point.

PRN only - antihistamine (chlorpheniramine 4-8mg PO or 10mg IV), paracetamol (1g PO or IV) to be given one hour prior to administration at local centre discretion.

~ Vital signs must be monitored during the infusion and the patient observed for 2 hours after the infusion has ended.

Veenith T, *et al. BMJ Open* 2021; 11:e050202. doi: 10.1136/bmjopen-2021-050202
