# Supplementary material for: CATALYST trial protocol: a multicentre, open-label, phase II, multiarm trial for an early and accelerated evaluation of the potential treatments for COVID-19 in hospitalised adults
Source: BMJ Open. 2021 Nov 11;11(11):e050202. doi: 10.1136/bmjopen-2021-050202 (PMC8587583; doi:10.1136/bmjopen-2021-050202)
Supplement: Supplementary data [file bmjopen-2021-050202supp006.pdf]

CATALYST

Supplementary Appendix 7 – Statistical considerations

The simulations and tables below demonstrate the operating characteristics of a trial design with the chosen decision criteria, based on a simpler analysis of the area under the curve for sequential CRP data, with effect sizes informed from a dataset from 1026 hospitalised COVID-19 patients at Queen Elizabeth Hospital, Birmingham. In our simulations, we compared a traditional fixed trial design recruiting 120 patients with candidate adaptive designs. We present basic operating characteristics for the fixed design (Table 6A) and the chosen adaptive design (Table 6B). We studied six scenarios of treatment effect, and estimated, through simulation, the probability of a trial stopping early for "success" or "futility," and ultimately concluding success. Simulations were performed in Fixed and Adaptive Clinical Trial Simulator (FACTS) software using default non-informative priors.

Table 6A. Operating characteristics for a fixed trial design of 120 patients.

Scenario	Probability stopping early for success	Probability stopping early for futility	Overall probability of success	Mean number of patients
Null	0	0	0.101	120
A	0	0	0.537	120
B	0	0	0.926	120
C	0	0	0.997	120
D	0	0	0.008	120
E	0	0	0	120

Scenarios A, B, and C are beneficial effects of the intervention with (true) treatment effects of 0.25, 0.5 and 0.75 standard deviations, "null" is zero treatment effect and D and E are harmful effects of 0.25 and 0.5 standard deviations. "success" and "futility" are defined as above.

Table 6B. Operating characteristics for an adaptive design with interim analyses at 40 and 80 patients.

Scenario	Probability stopping early for success	Probability stopping early for futility	Overall probability of success	Mean number of patients
Null	0.148	0.624	0.176	66
A	0.455	0.281	0.559	70
B	0.798	0.089	0.890	59
C	0.965	0.012	0.985	48
D	0.03	0.901	0.031	52
E	0.003	0.986	0.003	43

The adaptive design achieves similar probabilities of success in scenarios where the treatment effect is truly beneficial (A, B and C), and increases the probability of success only slightly if the intervention is harmful (D and E). There is some increase in the probability of success if the treatment effect is zero (Type I error) but this is offset by the very substantial reductions in the numbers of patients needed in all scenarios. Moreover, Type I error is not a serious problem as all interventions would be evaluated further in phase III trials.

Veenith T, *et al. BMJ Open* 2021; 11:e050202. doi: 10.1136/bmjopen-2021-050202
