# Supplementary material for: CATALYST trial protocol: a multicentre, open-label, phase II, multiarm trial for an early and accelerated evaluation of the potential treatments for COVID-19 in hospitalised adults
Source: BMJ Open. 2021 Nov 11;11(11):e050202. doi: 10.1136/bmjopen-2021-050202 (PMC8587583; doi:10.1136/bmjopen-2021-050202)
Supplement: Supplementary data [file bmjopen-2021-050202supp007.pdf]

Statistical Analysis Plan

A randomised phase II proof of principle multi-arm multi-stage trial designed to guide the selection of interventions for phase III trials in hospitalised patients with COVID-19 infection.

Version: 2.0
March 17, 2021

Sponsor(s): University of Birmingham
EudraCT Number: 2020-001684-89
Sponsor Reference Number: RG_20-030

Author(s):

**Name:**	Mr Daniel Slade
**Trial role:**	Statistical Lead
**Name:**	Miss Charlotte Gaskell
**Trial role:**	Trial Statistician
**Name:**	Professor Simon Gates
**Trial role:**	Statistical Lead & Co-Investigator

Reviewed & Approved by:

**Name:**	Dr. Benjamin Fisher
**Trial role:**	Acting Chief Investigator

Cancer Research UK Clinical Trials Unit
CRCTU

Veenith T, *et al. BMJ Open* 2021; 11:e050202. doi: 10.1136/bmjopen-2021-050202

Key personnel involved in the Statistical Analysis Plan:

Name	Trial role
Mr Daniel Slade	Statistical Lead
Miss Charlotte Gaskell	Trial Statistician
Dr. Tonny Venith	Chief Investigator
Dr. Benjamin Fisher	Deputy Chief Investigator
Professor. Simon Gates	Statistical Lead & Co-Investigator

Document Control Sheet:

Statistical Analysis Plan version:	Reason for update:
v1.0	Initial Version
v2.0	Incorporation of joint-modelling and AUC approach for the primary analysis, complimentary analyses to attempt to take account of censoring. Addition of ITT analysis for secondary endpoints and MITT definition for secondary endpoints. Modification of primary analysis to state inference will be based on only the interaction term of the model. Specification of subgroup analyses based on disease severity, a request of the DMC.

Veenith T, *et al. BMJ Open* 2021; 11:e050202. doi: 10.1136/bmjopen-2021-050202

Contents

1 INTRODUCTION 4
1.1 Purpose of the Statistical Analysis Plan . . . 4
1.2 Summary of the Trial . . . 4

2 TIMING AND REPORTING OF INTERIM AND FINAL ANALYSES 6

3 RECRUITMENT AND RANDOMISATION 6
3.1 Recruitment . . . 6
3.2 Randomisation . . . 6
3.3 Ineligible Patients . . . 6

4 DATA QUALITY 6
4.1 Data Quality: CRFs . . . 6
4.2 Return Rates: CRFs . . . 6

5 TRIAL POPULATION 7
5.1 Patient Characteristics . . . 7
5.2 Definition of Populations for Analysis . . . 7

6 TREATMENT RECEIVED 7

7 SAFETY ANALYSIS 7

8 ANALYSIS 7
8.1 Analysis of Primary Outcome Measure . . . 8
8.2 Analysis for Secondary Outcome Measures . . . 8
8.3 Subgroup Analysis . . . 9

9 SAMPLE SIZE 9

10 STATISTICAL SOFTWARE 10

11 STORAGE AND ARCHIVING 10

Veenith T, *et al. BMJ Open* 2021; 11:e050202. doi: 10.1136/bmjopen-2021-050202

Supplemental material BMJ Publishing Group Limited (BMJ) disclaims all liability and responsibility arising from any reliance placed on this supplemental material which has been supplied by the author(s) BMJ Open

1 INTRODUCTION

1.1 Purpose of the Statistical Analysis Plan

This Statistical Analysis Plan (SAP) provides guidelines for the analysis and presentation of results for the Catalyst trial. This plan, along with all other documents relating to the analysis of this trial, will be stored in the 'Statistical Documentation' section of the Trial Master File. The statistical analysis will be carried out by the trial Statisticians.

1.2 Summary of the Trial

Trial Design
Catalyst is a rapid, open-label, phase II, multi-arm, multi-stage trial permitting an efficient evaluation of the potential efficacy of these targeted drugs which can then be considered for larger-scale testing by one of the current national platform trials.

Objectives
Primary Objectives

- To investigate whether candidate treatments demonstrate evidence of greater attenuation of inflammation as defined by an improvement in C-reactive protein (CRP) concentrations compared with usual care in COVID-19 patients.
- To recommend drugs that should be evaluated further in one of the phase III trials.

Outcome Measures
Primary Outcome Measures

- C-reactive protein measured over time up to day 14 for each patient.

Secondary Outcome Measures

- World Health Organisation (WHO) Clinical Progression improvement Scale (1-10 scale; for the purposes of this trial level 0, no viral RNA detected, will not be assessed)
- The ratio of the oxygen saturation to fractional inspired oxygen concentration (SpO2/FiO2), measured from randomisation to day 14, hospital discharge or death. SpO2 and FiO2 are measured as part of routine clinical care
- Respiratory rate
- Body temperature
- NEWS-2 score
- Length of hospital stay
- Hospital survival status at day 28 / hospital free days
- Proportion of patients discharged at day 28
- Destination of discharge
- Lymphocyte and Neutrophil counts and ratios
- Ferritin, D-Dimer and LDH
- Adverse events (AEs) and Serious Adverse Events (SAEs) as recorded by Common Terminology Criteria for Adverse Events (CTCAE), version 4.03 of grade ≥ 3 with interest in veno-occlusive disease (VOD), secondary infection and allergic reaction
- Overall Survival

Exploratory Outcome Measures

Veenith T, et al. BMJ Open 2021; 11:e050202. doi: 10.1136/bmjopen-2021-050202

- Blood inflammatory mediators, biomarkers, transciptome and cellular immunology in relation to COVID-19 infection
- Viral load
- Host DNA assessed at baseline to assess for predictors of disease severity and drug response
- blood biomarkers of aveolar epithelial cell damage to include surfactant D and RAGE

Patient Population
This trial seeks to recruit hospitalised patients with COVID-19 who are hypoxic, admitted to either a hospital ward or ICU, and are at risk of deterioration.

Sample Size
A total of up to 60 patients per treatment arm will be recruited.

Veenith T, *et al. BMJ Open* 2021; 11:e050202. doi: 10.1136/bmjopen-2021-050202

2 TIMING AND REPORTING OF INTERIM AND FINAL ANALYSES

There two planned interim analyses for the primary endpoint at n=20 and n=40 per arm respectively. Data analyses pertaining to trial conduct, data quality and patient safety will be supplied in confidence to an independent DMC throughout the period the trial is running. The DMC shall review the available data on a proposed 3 monthly basis.

The final analyses for the trial will be conducted once the end of trial has been reached. The final analyses will incorporate the primary, secondary and all exploratory outcomes as detailed in this analysis plan. The end of trial is defined as 6 months after the last data capture.

3 RECRUITMENT AND RANDOMISATION

3.1 Recruitment

At the point of analysis the following data will be reported:

- Date of the database snapshot used for recruitment analysis
- Total number of patients who have been recruited into the trial and randomised to each treatment arm
- Recruitment over time (monthly and cumulative)
- Recruitment by site

3.2 Randomisation

Patients will be randomised 1:1 between Usual Care (Control Arm) and interventional arms using the minimisation procedure described by Pocock and Simon, with a single stratification variable with two levels; Care status: 'On ward' or 'ICU'. Patients will be randomised into either a control group or to receive interventional treatments that are available at their site.

3.3 Ineligible Patients

Ineligible patients are defined as those registered patients who are subsequently found to not meet the eligibility criteria of the trial after being recruited. The proportion of ineligible patients and reasons for their ineligibility will be reported for each treatment arm. In addition the number of patients who were screened in total will be reported along with the number of patients not recruited to the trial and their associated reasons e.g. ineligible.

4 DATA QUALITY

4.1 Data Quality: CRFs

Patient data is collected using case report forms (CRFs) and electronic case report forms (eCRFs). Data collected in this way will be stored on a trial database. The trial database will be checked for missing data and any discrepancies at least annually but prior to any analysis as according to the trial specific data validation plan, which will be developed by both the trial statisticians and the trial coordinator.

4.2 Return Rates: CRFs

The proportion of returned CRFs compared to those that were expected will be reported for each case report form.

5 TRIAL POPULATION

5.1 Patient Characteristics

A summary of patient characteristics will be reported. Descriptive statistics will be provided in the summary including counts and percentages for categorical data items and mean (sd), median and ranges for continuous data items.

5.2 Definition of Populations for Analysis

Safety Population - Safety population will include all patients who receive any trial treatment. For interventional arms this requires the patient to have received some IMP.

MITT Population - The Modified Intention-To-Treat population for the primary analyses will include all patients who receive any trial treatment and who have a baseline CRP measurement and at least one further CRP measurement post baseline. For the secondary endpoints, this includes all patients who receive any trial treatment and have available data for the respective outcome measure.

ITT Population - This includes all randomised patients in their treatment arms, that have available data for the respective outcome measure.

6 TREATMENT RECEIVED

For each treatment arm, the proportion of participants who received treatment as per protocol will be reported. The proportion of participants who discontinued treatment early will also be reported along with a tabulation of the reasons. Summary statistics for all participants on treatment arms will be reported e.g. median/mean time on treatment, these statistics will be tailored for the specific arms as naturally the treatments may widely differ and thus different summary measures will be relevant.

7 SAFETY ANALYSIS

The number of serious adverse events (including SARs and SUSUARs), and the number of treatment-related deaths will be reported for each treatment arm. The reporting period for Adverse Events/Serious Adverse Events (SAE's) will commence from the date of consent. Safety will be assessed by looking at adverse events (CTCAE).

The following details will be reported for each treatment arm for all patients who are part of the safety population:

- Adverse events at baseline, summarised by event and number of patients experiencing such events.
- Max grade experienced for all patients.
- A summary of number of events and patients for all toxicities by event and grade.
- The number of events and patients for all grades of toxicities.
- All serious adverse events will be reported, details to be presented include but are not limited to; admitting event, other events, reason for SAE, outcome, sequel and relatedness.

8 ANALYSIS

For all analyses data will be analysed for each intervention against the control group, including in each analysis only those participants who were eligible for the those treatment arms at the point of randomisation. The primary analysis will be conducted on the MITT population and all secondary analyses will be conducted on both the MITT population and ITT unless otherwise specified.

New intervention arms may be added as new interventions become available. All comparisons will be performed temporally with regards to control arm data.

Veenith T, *et al. BMJ Open* 2021; 11:e050202. doi: 10.1136/bmjopen-2021-050202

8.1 Analysis of Primary Outcome Measure

The CRP data will be modelled using Bayesian multi-level models that allow for nesting of the repeated measures data within patient, and allowing for non-linear responses. This approach will facilitate an assessment of the effects of the treatments on the CRP. Specifically, posterior probabilities for the treatment/time interaction term will be used to conduct decision making. Care status as a randomisation stratification factor will be incorporated accordingly into the model structure along with age as a known prognostic indicator.

At the specified decision points, with interim analyses at n=20 and n=40 and a final analysis at n=60 per arm, the CRP data will be considered in the context of the emerging safety data to make a recommendation as outlined below:

a) If there is strong evidence of an additional anti-inflammatory effect (CRP) and a satisfactory safety profile consider progression to clinical endpoint evaluation whether in this trial or in another one

b) Terminate arm and do not proceed (based on lack of evidence of an additional biological effect or of an unfavourable safety signal)

We will define that 'strong evidence' or 'success' will be if there is an 90% probability that the intervention arm is better than usual care in reducing CRP as seen by the treatment/time interaction covariate. 'lack of evidence' or 'futility' is defined as less than 50% probability of the intervention being better than usual care. However, given the large number of agents being investigated in various phase II trials, the size of effect and the totality of data will be reviewed before recommending adoption by a phase III platform.

In addition to the above analysis we will analyse the data using two further approaches, namely, modelling AUC and an additional joint-modelling approach for CRP and discharge/death, this is to ascertain if censoring events for CRP; discharge/death, have had any impact on inference and if so to model accordingly.

8.2 Analysis for Secondary Outcome Measures

<u>Outcome measures</u>

- World Health Organisation (WHO) Clinical Progression improvement Scale
  - Time to improvement, measured from the date of randomisation, an event here is defined as at least a one-point improvement on the Time to Clinical Improvement Scale. A Kaplan-Meier plot will be produced for each treatment and control arm comparison, estimates of median time to improvement will be reported along with associated confidence intervals (where they can be estimated). In addition to the one-point improvement an additional analysis utilising a two-point improvement will be conducted, to be comparable with other studies.
  - Patients' scores on the Clinical Improvement Scale for each day will be displayed graphically, and modelled using Bayesian longitudinal ordinal regression, as described by Harrell (`http://hbiostat.org/proj/covid19/bayesplan.html`).
- The ratio of the oxygen saturation to fractional inspired oxygen concentration (SpO2/FiO2) will be presented graphically over time.
- Length of hospital stay will be summarised via descriptive statistics, stratified by treatment group. Reasons for such lengths of stay will be reported and summarised accordingly.
- Respiratory rate, body temperature and NEWS-2, will be plotted over time and summarised through descriptive statistics. These measures may also be modelled over time using multilevel modelling. Exploratory data analysis will drive model formulation, assumptions will be tested accordingly. All modelling will be exploratory in nature.
- The proportion of patients discharged at day 28 along with destination of discharge will be presented accordingly.
- Hospital survival status at 28 days will be reported as a tabulation of the proportion of patients who have died, been discharged or are still in hospital by day 28. Hospital-free days will be summarised through descriptive statistics, patients still in hospital or who have died will be incorporated having 0 hospital-free days.

- Lymphocyte, neutrophil and full blood counts with lymphocyte: neutrophil ratios and ferritin, D-Dimer and Triglycerides LDH values will be plotted over time and summarised through descriptive statistics. These measures may also be modelled over time using multilevel modelling. Exploratory data analysis will drive model formulation, assumptions will be tested accordingly. All modelling will be exploratory in nature.
- AEs and SAEs will be analysed as per section 7
- Overall Survival - Measured from the date of registration, an event here is defined as death. Patients are followed up until they have either died or are censored at date last seen. A Kaplan-Meier plot will be produced for each comparison, estimates of median survival will be reported along with associated confidence intervals (where they can be estimated)

8.3 Subgroup Analysis

Exploratory subgroup analyses will be conducted to attempt to ascertain the effect of disease severity on outcomes. The subgroups of 'non-severe disease' and 'severe disease' are defined as those that have a baseline WHO score of < 6 and ≥ 6 respectively. Other exploratory subgroup analyses may be conducted based on known prognostic indicators e.g. age group.

9 SAMPLE SIZE

The tables below demonstrate the operating characteristics of a trial design with the chosen decision criteria, based on a simpler analysis of area under the curve for sequential CRP data, with effect sizes informed from a dataset from 1026 hospitalised COVID-19 patients at Queen Elizabeth Hospital, Birmingham.

It is anticipated that our proposed hierarchical analysis will have superior operating characteristics. In our simulations, we compared a traditional fixed trial design recruiting 120 patients with candidate adaptive designs. We present basic operating characteristics for the fixed design (Table 1) and the chosen adaptive design (Table 2). We studied six scenarios of treatment effect, and estimated, through simulation, the probability of a trial stopping early for "success" or "fultility," and ultimately concluding success. Scenarios A, B, and C are beneficial effects of the intervention with (true) treatment effects of 0.25, 0.5 and 0.75 standard deviations, "null" is zero treatment effect and D and E are harmful effects of 0.25 and 0.5 standard deviations. "success" and "futility" are defined as above.

Table 1: Operating characteristics for a fixed trial design of 120 patients

Scenario	Probability stopping early for success	Probability stopping early for futility	Overall probability of success	Mean number of patients
Null	0	0	0.101	120
A	0	0	0.537	120
B	0	0	0.926	120
C	0	0	0.997	120
D	0	0	0.008	120
E	0	0	0	120

The adaptive design achieves similar probabilities of success in scenarios where the treatment effect is truly beneficial (A, B and C), and increases the probability of success only slightly if the intervention is harmful (D and E). There is some increase in the probability of success if the treatment effect is zero (Type I error) but this is offset by the very substantial reducitions in the numbers of patients needed in all scenarios. Moreover, Type I error is not seen as a serious problem as all interventions would be evaluated further in Phase 3 trials.

Veenith T, *et al. BMJ Open* 2021; 11:e050202. doi: 10.1136/bmjopen-2021-050202

Table 2: operating characteristics for an adaptive design with interim analyses at 40 and 80 patients

Scenario	Probability stopping early for success	Probability stopping early for futility	Overall probability of success	Mean number of patients
Null	0.140	0.607	0.143	70
A	0.471	0.254	0.573	74
B	0.847	0.062	0.910	61
C	0.974	0.010	0.989	51
D	0.030	0.918	0.031	54
E	0.003	0.985	0.003	47

10 STATISTICAL SOFTWARE

Statistical analyses will be carried out using relevant statistical software; SAS , Stata or R respectively. Version numbers and session details will be stated and logged with any analysis.

11 STORAGE AND ARCHIVING

Catalyst files are stored in a restricted access directory on a secure server and will be saved for archive purposes according to CRCTU policy and procedure.

Veenith T, *et al. BMJ Open* 2021; 11:e050202. doi: 10.1136/bmjopen-2021-050202
