# Supplementary material for: CATALYST trial protocol: a multicentre, open-label, phase II, multiarm trial for an early and accelerated evaluation of the potential treatments for COVID-19 in hospitalised adults
Source: BMJ Open. 2021 Nov 11;11(11):e050202. doi: 10.1136/bmjopen-2021-050202 (PMC8587583; doi:10.1136/bmjopen-2021-050202)
Supplement: Author's manuscript [file bmjopen-2021-050202.draft_revisions.pdf]

BMJ Open is committed to open peer review. As part of this commitment we make the peer review history of every article we publish publicly available.

When an article is published we post the peer reviewers' comments and the authors' responses online. We also post the versions of the paper that were used during peer review. These are the versions that the peer review comments apply to.

The versions of the paper that follow are the versions that were submitted during the peer review process. They are not the versions of record or the final published versions. They should not be cited or distributed as the published version of this manuscript.

BMJ Open is an open access journal and the full, final, typeset and author-corrected version of record of the manuscript is available on our site with no access controls, subscription charges or pay-per-view fees (http://bmjopen.bmj.com).

If you have any questions on BMJ Open's open peer review process please email info.bmjopen@bmj.com

BMJ Open

CATALYST trial protocol: A multicentre, open-label, phase II, multi-arm trial for an early and accelerated evaluation of the potential treatments for COVID-19 in hospitalised adults

Journal:	*BMJ Open*
Manuscript ID	bmjopen-2021-050202
Article Type:	Protocol
Date Submitted by the Author:	16-Feb-2021
Complete List of Authors:	Veenith, Tonny; University of Birmingham, Birmingham Acute Care Research Group, Institute of Inflammation and Ageing, College of Medical and Dental Sciences; University Hospitals Birmingham NHS Foundation Trust, Department of Critical Care Medicine<br>Fisher, Benjamin; University of Birmingham College of Medical and Dental Sciences, Institute of Inflammation and Ageing<br>Slade, Daniel; University of Birmingham, Cancer Research UK Clinical Trials Unit, Institute of Cancer and Genomic Sciences<br>Rowe, Anna; University Hospitals Birmingham NHS Foundation Trust, National Institute for Health Research (NIHR) Birmingham Biomedical Research Centre; University of Birmingham, Cancer Research UK Clinical Trials Unit, Institute of Cancer and Genomic Sciences<br>Sharpe, Rowena; University of Birmingham, Cancer Research UK Clinical Trials Unit, Institute of Cancer and Genomic Sciences<br>Thickett , David ; University of Birmingham, Birmingham Acute Care Research Group, Institute of Inflammation and Ageing, College of Medical and Dental Sciences, ; University Hospitals Birmingham NHS Foundation Trust, Department of Respiratory Medicine<br>Whitehouse, Tony; University Hospitals Birmingham NHS Foundation Trust, Department of Critical Care Medicine; University of Birmingham, Birmingham Acute Care Research Group, Institute of Inflammation and Ageing, College of Medical and Dental Sciences,<br>Rowland, Matthew; University of Oxford, Nuffield Department of Clinical Neurosciences, John Radcliffe Hospital,<br>Scriven, James; University Hospitals Birmingham NHS Foundation Trust, Department of Infectious Diseases<br>Parekh, Dhruv; University of Birmingham, Birmingham Acute Care Research Group, Institute of Inflammation and Ageing, College of Medical and Dental Sciences, ; University Hospitals Birmingham NHS Foundation Trust, Department of Critical Care Medicine, and Department of Respiratory Medicine<br>Bowden, Sarah; University of Birmingham, Cancer Research UK Clinical Trials Unit, Institute of Cancer and Genomic Sciences,<br>Savage, Joshua; University of Birmingham, Cancer Research UK Clinical Trials Unit, Institute of Cancer and Genomic Sciences,<br>Richards, Duncan; University of Oxford, Oxford Clinical Trials Research Unit, Botnar Research Centre,<br>Bion, Julian; University Hospitals Birmingham NHS Foundation Trust, Department of Critical Care Medicine,

Kearns, Pamela; University Hospitals Birmingham NHS Foundation Trust, National Institute for Health Research (NIHR) Birmingham Biomedical Research Centre, ; University of Birmingham, Cancer Research UK Clinical Trials Unit, Institute of Cancer and Genomic Sciences,
Gates, Simon; University of Birmingham, Cancer Research UK Clinical Trials Unit, Institute of Cancer and Genomic Sciences,

Keywords: Adult intensive & critical care < ANAESTHETICS, COVID-19, INFECTIOUS DISEASES, Thoracic medicine < INTERNAL MEDICINE, Rheumatology < INTERNAL MEDICINE

SCHOLARONE™
Manuscripts

I, the Submitting Author has the right to grant and does grant on behalf of all authors of the Work (as defined in the below author licence), an exclusive licence and/or a non-exclusive licence for contributions from authors who are: i) UK Crown employees; ii) where BMJ has agreed a CC-BY licence shall apply, and/or iii) in accordance with the terms applicable for US Federal Government officers or employees acting as part of their official duties; on a worldwide, perpetual, irrevocable, royalty-free basis to BMJ Publishing Group Ltd ("BMJ") its licensees and where the relevant Journal is co-owned by BMJ to the co-owners of the Journal, to publish the Work in this journal and any other BMJ products and to exploit all rights, as set out in our licence.

The Submitting Author accepts and understands that any supply made under these terms is made by BMJ to the Submitting Author unless you are acting as an employee on behalf of your employer or a postgraduate student of an affiliated institution which is paying any applicable article publishing charge ("APC") for Open Access articles. Where the Submitting Author wishes to make the Work available on an Open Access basis (and intends to pay the relevant APC), the terms of reuse of such Open Access shall be governed by a Creative Commons licence – details of these licences and which Creative Commons licence will apply to this Work are set out in our licence referred to above.

Other than as permitted in any relevant BMJ Author's Self Archiving Policies, I confirm this Work has not been accepted for publication elsewhere, is not being considered for publication elsewhere and does not duplicate material already published. I confirm all authors consent to publication of this Work and authorise the granting of this licence.

For peer review only

CATALYST trial protocol: A multicentre, open-label, phase II, multi-arm trial for an early and accelerated evaluation of the potential treatments for COVID-19 in hospitalised adults

Tonny Veenith[1, 2]*, Benjamin A. Fisher[3,4,5]*, Daniel Slade[5], Anna Rowe[4,5], Rowena Sharpe[5], David R. Thickett[2,6], Tony Whitehouse[1,2], Matthew Rowland[7], James Scriven[8], Dhruv Parekh[1,2,6], Sarah J. Bowden[5], Joshua S. Savage[5], Duncan Richards[9], Julian Bion[1]†, Pamela Kearns[4,5]†, Simon Gates[5]†, on behalf of CATALYST investigators[&]

* These authors have made an equal contribution.

† These authors have made an equal contribution.

[&] Additional investigators of the CATALYST Trial are listed in Supplemental Appendix 1.

* Corresponding authors contact details:

Prof. Tonny Veenith, Institute of Inflammation and Ageing, College of Medical and Dental Sciences, University of Birmingham, Birmingham, B15 2TT, UK. Email: t.v.veenith@bham.ac.uk or;

Dr Benjamin A. Fisher, Institute of Inflammation and Ageing, College of Medical and Dental Sciences, University of Birmingham, Birmingham, B15 2TT, UK. Email: b.fisher@bham.ac.uk.

1. Department of Critical Care Medicine, University Hospitals Birmingham NHS Trust, United Kingdom
2. Birmingham Acute Care Research Group, Institute of Inflammation and Ageing, College of Medical and Dental Sciences, University of Birmingham, UK
3. Rheumatology Research Group, Institute of Inflammation and Ageing, College of Medical and Dental Sciences, University of Birmingham, UK
4. National Institute for Health Research (NIHR) Birmingham Biomedical Research Centre, University Hospitals Birmingham NHS Foundation Trust, Birmingham, UK.
5. Cancer Research UK Clinical Trials Unit, Institute of Cancer and Genomic Sciences, University of Birmingham, Birmingham, UK
6. Department of Respiratory Medicine, University Hospitals Birmingham NHS Trust, UK
7. Nuffield Department of Clinical Neurosciences, University of Oxford, John Radcliffe Hospital, Oxford, UK.
8. Department of Infectious Diseases, University Hospitals Birmingham NHS Trust, UK
9. Oxford Clinical Trials Research Unit, Botnar Research Centre, University of Oxford, Oxford, UK.

For peer review only - http://bmjopen.bmj.com/site/about/guidelines.xhtml

Abstract

Introduction

Severe SARS-CoV-2 infection is associated with a dysregulated immune response. Inflammatory monocytes and macrophages are crucial, promoting injurious, pro-inflammatory sequelae. Immunomodulation is, therefore, an attractive therapeutic strategy and we sought to test licensed and novel candidate drugs.

Methods and analysis

The CATALYST trial is a multi-arm, open-label, multi-centre, phase II platform trial designed to identify candidate novel treatments to improve outcomes of patients hospitalised with COVID-19 compared with usual care. Treatments with evidence of biomarker improvements will be put forward for larger-scale testing by current national phase III platform trials. Hospitalised patients ≥16 years with a clinical picture strongly suggestive of SARS-CoV-2 pneumonia (confirmed by chest X-ray or CT scan, with or without a positive reverse transcription polymerase chain reaction (RT-PCR) assay) and a C-Reactive Protein (CRP) ≥40 mg/L are eligible. The primary outcome measure is CRP, measured serially from admission to day 14, hospital discharge or death. Secondary outcomes include the WHO Clinical Progression Improvement Scale as a principal efficacy assessment.

Ethics and dissemination

The protocol was approved by the East Midlands - Nottingham 2 Research Ethics Committee (20/EM/0115) and given Urgent Public Health status; initial approval was received on 05-May-2020, current protocol version (v6.0) approval on 12-Oct-2020. The MHRA also approved all protocol versions. The results of this trial will be disseminated through national and international presentations and peer-reviewed publications.

Trial registration number

EudraCT Number: 2020-001684-89

ISRCTN Number: 40580903

Strengths and limitations of this trial

- CATALYST will provide a rapid readout on the safety and proof-of-concept of candidate novel treatments
- CATALYST will enable phase III trial resources to be focussed and allocated for agents with a high likelihood of success
- CATALYST uses Bayesian multi-level models to allow for nesting of repeated measures data, with factors for each individual patient and treatment arm, and allowing for non-linear responses
- CATALYST is not designed to provide a definitive signal on clinical outcomes

For peer review only - http://bmjopen.bmj.com/site/about/guidelines.xhtml

Introduction

Since the start of the pandemic, the UK has now passed the milestone of 100,000 deaths due to SARS-CoV-2 virus. Current data from the International Severe Acute Respiratory and emerging Infections Consortium (ISARIC) partnerships indicate that 20% of patients admitted to the hospital require critical care support, with an overall mortality of 34.5% after hospital admission in the last two waves (1). Mortality following respiratory support remains over 40% in the second wave (2). Recovery from severe disease may be associated with long-term health impact (3, 4). Despite the introduction of vaccination programmes in December-2020, there remains an urgent need to identify agents which prevent progression to critical illness, reduce morality and promote rapid recovery.

SARS-CoV-2 can cause severe pneumonia with diffuse alveolar damage, infiltrating perivascular lymphocytes, disrupted endothelial cell membranes, vascular thrombosis with microangiopathy and occlusion of alveolar capillaries (5). Subsequent multiple organ failure, is in part, driven by a dysregulated immune response. Inflammatory monocytes and macrophages contribute to endothelial damage and microthrombosis and drive cytokine production. The cellular response is characterised by an upregulation of pro-inflammatory cytokines and chemokines, leading to a host immune response targeted at the virus, damaging host tissues (6). The severity of the disease is proportional to the cytokine response (interleukin (IL)-6, interferon gamma inducible protein (IP)-10, monocyte chemoattractant protein (MCP)-1, macrophage inflammatory protein (MIP)-1A, and tumour necrosis factor (TNF)-α), with critically ill patients exhibiting the highest levels of cytokines and chemokines (7-9). This dysregulated immunity may be a modifiable pathobiological therapeutic target for preventing COVID-19 progression. Potential mechanisms underlying this immune pathobiology may be targeted with precision using existing licensed and novel drugs. The CATALYST platform, therefore, aims to study these drugs and other novel therapeutic options for rapid assessment of safety, biological signal for efficacy, and providing secure underpinning of science prior to large phase III trials. Those with potential efficacy can then be considered for larger-scale testing by national platform trials such as RECOVERY (10) or REMAP-CAP (11).

Methods and analysis

Study design

The CATALYST trial is a multi-arm, randomised, open-label phase II clinical trial. Initial candidate drugs included within the multi-arm design were namilumab, and infliximab, monoclonal antibodies targeting the pro-inflammatory cytokines granulocyte-macrophage colony-stimulating factor (GM-CSF) and TNF-α respectively. A third agent, gemtuzumab-ozogamicin (Mylotarg), was incorporated in the protocol but has not been prioritised for evaluation at this stage and has not opened to recruitment. Each candidate therapy will be given in addition to usual care and compared with usual care independently (Figure 1). Randomisation will be performed according to a 1:1 ratio (usual care versus research arms) using a minimisation procedure to stratify based on ward- or intensive care unit (ICU)-based care. New therapies may be introduced sequentially utilising this design. Therapies for evaluation will be based on the scientific rationale prioritised by a Scientific Advisory Board (SAB). This will enable the rapid addition of new treatment arms, whilst allowing the efficacious assessment and analysis of each of these treatment arms (12, 13). Participants are followed up for at least 28 days post-randomisation.

Patient and public involvement (PPI)

From its inception, the CATALYST trial was co-developed with the Critical Care PPI group based in the Surgical Reconstruction and Microbiology Research Centre (SRMRC) at University Hospitals Birmingham. The group reviewed and refined the protocol and participant-facing documents and provided input into

For peer review only - http://bmjopen.bmj.com/site/about/guidelines.xhtml

the design; specifically, their feedback supported a usual care arm without the inclusion of placebo controls given the context of the ongoing pandemic. PPI representatives are members of the Trial Steering Committee (TSC) who will supervise the conduct of the trial conduct, and monitor progress, including recruitment, and will support the dissemination of the trial results.

Inclusion and exclusion criteria

Patients aged 16 years or over, with a clinical picture strongly suggestive of SARS-CoV-2 pneumonia (confirmed by chest X-ray or CT scan, with or without a positive reverse transcription-polymerase chain reaction (RT-PCR) assay), and C-Reactive Protein (CRP) greater than or equal to 40 mg/L are eligible for this study. Exclusion criteria include patient or personal/professional legal representative refusal, planned palliative care, pregnancy or breastfeeding women, women of childbearing potential and non-vasectomised men who are unwilling to use effective contraception for the duration of the trial and throughout the drug-defined post-trial period. Patients with known HIV or chronic hepatitis B or C infection, contraindications to any of the Investigational Medicinal Products, receiving concurrent immunosuppression with biological agents, a history of haematopoietic stem cell transplant or solid organ transplant, known hypersensitivity to drug products or excipients, tuberculosis or other severe infections such as (non-SARS-CoV-2) sepsis, abscesses, and opportunistic infections requiring treatment, moderate or severe heart failure (NYHA class III/IV), or any other indication or medical history, that in the opinion of the patient's local investigator is unsuitable for trial participation. Patients will not be eligible if they are currently participating in another COVID-19 interventional trial; co-enrolment into RECOVERY-Respiratory Support (RS) trial is allowed.

Consent

Patients are identified as per site-established processes. This may include searching central logs of patients admitted with COVID-19, or via pre-screening processes already in situ at the site. Each eligible patient who has capacity will be given a Patient Information Sheet (PIS) to read more about the trial (Supplementary Appendix 2). Informed consent is requested from patients with capacity by an investigator who has been delegated the responsibility on the delegation log. Where a patient lacks capacity (for example from severity of illness) informed consent will be sought from the patient's personal legal representative (PerLR). In the event that the PerLR is unavailable, informed consent will be sought from the patient's professional legal representative (ProfLR) according to the requirements of the UK Health Research Authority (14). Specific PISs for both PerLR and ProfLR can also be emailed to aid this process. If a patient recovers their capacity, they should be reconsented as soon as possible using the standard PIS and Informed Consent Form (ICF). Patient and PerLR forms are also available in Bengali, English, French, Polish, Portuguese, Punjabi, Urdu, and Welsh. Supplementary Appendices 3 and 4 contain the English version of the patient, ProfLR and PerLR information sheets, and ICFs, respectively.

Interventions

Arm 1: Usual care provided following the current institutional policy for patients with COVID-19. Following the recommendation from the UK CMO in June 2020, standard care includes dexamethasone treatment.

Arm 3: Usual care combined with namilumab, administered in a single dose (150mg) on day one infused intravenously over 1-hour. Namilumab is an anti-GM-CSF monoclonal antibody with a good safety profile up to phase II studies in rheumatoid arthritis (RA) and axial spondyloarthropathy with over 360 individuals dosed in total (15-18). The objective of namilumab therapy in COVID-19 is to inhibit inflammatory

For peer review only - http://bmjopen.bmj.com/site/about/guidelines.xhtml

monocyte/macrophage activation and their trafficking to the lungs so as to reduce the aberrant immunopathology.

Arm 4: Usual care combined with infliximab, administered in a single dose of 5mg/Kg diluted in 250ml of 0.9% saline on day one and infused over a 2-hour period. Infliximab is a widely available anti-TNF alpha monoclonal antibody licensed for the treatment of a number of diseases including RA. TNF is a key pro-inflammatory cytokine produced by macrophages implicated in a number of processes contributing to early lung pathology (19).

No dose modification for namilumab or infliximab is permitted. Although there are no requirements for pre-medication, patients may receive pre-medication or treatment with antihistamines and paracetamol at local discretion to prevent or treat mild-to-moderate infusion reactions due to namilumab or infliximab administration.

Initially, gemtuzumab-ozogamicin, an antibody-drug conjugate approved for induction therapy of acute myeloid leukaemia was included for investigation in the trial (Arm 2), however, prioritisation discussions in the Government Committee overseeing the COVID-19 phase II studies, advised that this arm should be suspended without recruitment, in favour of continuing with namilumab and infliximab arms.

Trial outcomes

The primary outcome is CRP concentration, where a sequential reduction in one of the interventional arms as compared with usual care is considered indicative that this may be a clinically effective treatment suitable for testing in phase III clinical trials.

The secondary outcomes are aligned with Core Outcome Measures in Effectiveness Trials' initiatives (20, 21). The principal clinical efficacy measure is the World Health Organisation (WHO) Clinical Progression Improvement Scale measured daily for 28 days on a 1-10 scale; level 0 (no viral load detected) will not be assessed over the course of this study (Table 1). Other clinical measures assessed until day 14, discharge, or death, include the ratio of the oxygen saturation to fractional inspired oxygen concentration (SpO_2/FiO_2), respiratory rate, body temperature, and the National Early Warning Scale 2 (NEWS-2). Assessments assessed until day 28 include length of hospital stay, hospital survival status at day 28, the proportion of patients discharged at day 28, and the destination of discharge. Routine laboratory measurements at baseline, days 3, 5, 7, 9, and 14 include lymphocyte count, neutrophil count, neutrophil: lymphocyte ratio, ferritin, D-Dimer and LDH. Safety measures as defined by adverse events (AEs) and as recorded by Common Terminology Criteria for Adverse Events (CTCAE), version 4.03 (22) are those of Grade ≥3, secondary infection and allergic reaction, and survival status.

In a subset of the patients (those admitted to University Hospitals Birmingham NHS Trust and University of Oxford NHS Trust) optional samples consisting of whole blood (for RNA, DNA and also cellular assessments, preserved in Cytodelics buffer), peripheral blood mononuclear cells (PBMCs), and plasma, will be obtained on days one, three and nine (or day of discharge if earlier) and will broadly follow the ISARIC protocol (23). All samples will be collected in accordance with national regulations and requirements, including standard operating procedures for logistics and infrastructure. Samples will be taken in appropriately licensed premises, stored and transported per the Human Tissue Authority guidelines and NHS trust policies.

Statistical analysis plan

The primary outcome data will consist of a sequence through time of readings of each patient's CRP. These will be modelled using Bayesian multi-level models (also known as hierarchical or mixed-effects) that allow for nesting of the repeated measures data within patient, and allowing for non-linear responses.

For peer review only - http://bmjopen.bmj.com/site/about/guidelines.xhtml

Specifically, posterior probabilities for the treatment/time interaction covariates will be used to conduct decision making. Data will be analysed for each intervention arm against the control group, including in each analysis, only participants who were eligible for that comparison. Any important covariates will be incorporated accordingly into the model structure e.g., age and care status.

At the specified decision points, with interim analysis at n=20 and n=40 and a final analysis at n=60 per arm, CRP data will be considered in the context of the emerging safety data to make a recommendation as outlined below:

a) If there is strong evidence of an additional inflammatory effect (CRP) and a satisfactory safety profile consider progression to clinical endpoint evaluation whether in this trial or in another one;
b) Terminate arm and do not proceed (based on lack of evidence of an additional biological effect or of an unfavourable safety signal).

Success will be declared if there is a 90% probability that the intervention arm is better than usual care in reducing CRP. Futility is defined as less than 50% probability of the intervention being better than usual care. However, given the large number of agents being investigated in various phase II trials, the size of effect and the totality of data will be reviewed before recommending adoption by a phase III platform. In the event of a successful treatment being identified and the effect size being large, consideration may be given to continuing the arm within CATALYST to study clinical efficacy (based on the WHO scale), if this were deemed to be a more efficient path than translation to a phase III platform. More information, including the operating characteristics based on a simpler analysis of the area under the curve for sequential CRP data are included in Supplemental Appendix 4.

New arms will be added as new interventions become available. All comparisons will be performed temporally with regards to the control arm data as new arms are added A detailed secondary outcome measure analysis can be found in the Statistical Analysis Plan. Exploratory subgroup analyses will be conducted to ascertain the effect of treatment on the primary outcome measure within care status strata. Analyses will be conducted as per the primary outcome measure with any inference based on the treatment/time interaction covariate included in the model formulation.

Adverse events reporting and analysis

The collection and reporting of AEs will be in accordance with the Research Governance Framework for Health and Social Care and the requirements of the National Research Ethics Service (NRES). Definitions of different types of AEs are listed in Supplementary Appendix 5. The reporting period for AEs will be between the date of commencement of protocol-defined treatment until day 28. The investigator should assess the seriousness and causality (relatedness) of all AEs experienced by the patient (this should be documented in the source data) with reference to the protocol. Abnormal laboratory findings will only be reported if they satisfy one of the following; (a) events which are Grade 3 or above; (b) events which result in the early discontinuation of trial treatment (if applicable to the research arm); or (c) events which result in a dose modification or dose interruption (if applicable to the research arm). Pre-existing conditions and pre-existing abnormal laboratory findings will only be reported if the condition worsens by at least one CTCAE grade. Hospitalisations for pre-planned elective procedures, unless the condition worsens, will not be reported as Serious (S)AEs.

Data management

Data will be collected via a set of forms capturing details of eligibility, baseline characteristics, treatment and outcome details created using FORMAP Case Report Form design software developed by Birmingham Clinical Trials Unit at the University of Birmingham. This trial will use an electronic remote data capture

For peer review only - http://bmjopen.bmj.com/site/about/guidelines.xhtml

system, with the exception of SAE reporting and notification of pregnancy; these are both paper-based. All trial records must be archived and securely retained for at least 25 years. No documents will be destroyed without prior approval from the sponsor, via the central CATALYST Trial Office. On-site monitoring will be carried out as required following a risk assessment and as documented in the Quality Management Plan. Any monitoring activities will be reported to the central CATALYST Trial Office and any issues noted will be followed up to resolution. CATALYST will also be centrally monitored, which may trigger additional on-site monitoring. Further information regarding data management is provided in the study protocol.

Trial organisation structure

The University of Birmingham will act as single sponsor for this multi-centre study. The trial is being conducted under the auspices of the Cancer Research UK Clinical Trials Unit (CRCTU), University of Birmingham, in close partnership with the National Institute for Health Research (NIHR) Biomedical Research Centres (BRC) at the Universities of Birmingham and Oxford, University College London (UCL) and Imperial College London.

Given the combination of a novel disease, a range of novel potential therapies and a pandemic setting, a multidisciplinary collaboration was essential, bringing together experts in acute, respiratory and intensive care medicine, inflammation, oncology, data sciences, trials methodology and statistics. The Birmingham Acute Care Research group provides a single point of reference for the acute specialities (24).

The trial management group (TMG) is responsible for the day-to-day running and management of the trial. Members include the Chief Investigator, Deputy Chief Investigator, Co-investigators, Trial Statisticians, Trial Management Team Leader and Trial Coordinator. The TMG reports to the TSC.

The TSC provides oversight and governance. Members include independent clinicians and patient advocates. The TSC supervises the conduct of the trial, monitoring progress including recruitment, data completeness, losses to follow-up, and deviations from the protocol. They will make recommendations about conduct and continuation of the trial.

The independent data monitoring committee (DMC) includes clinicians and a statistician who will review unblinded data analyses to advise the TSC on whether the trial data (and results from other relevant research), justifies the continuing recruitment of further patients. The DMC will operate in accordance with a trial-specific charter based on the template created by the Damocles Group. The DMC will review the trial data three-monthly during the recruitment and while patients remain on treatment. These may occur more frequently if the DMC deem necessary or for interventions which have not previously been administered to patients in this specific setting.

The SAB makes recommendations on prioritising interventions and aspects of methodology such as co-enrolment in order to harmonise trial activities with other research platforms. The SAB includes multi-stakeholder representatives (detailed in Supplementary Appendix 1) including, the collaborating centres and the associated Biomedical Research Centres (25) in Birmingham (26), Oxford (27), and University College London (28).

Confidentiality statement

Confidential trial data will be stored in accordance with the General Data Protection Regulation (GDPR) 2018. As specified in the patient information sheet and with the patient's consent, patients will be identified using only their date of birth and unique trial ID number.

For peer review only - http://bmjopen.bmj.com/site/about/guidelines.xhtml

Trial status

Recruitment for the trial opened in May 2020 with the namilumab and infliximab arms. Although included in the protocol, the gemtuzumab-ozogamicin arm has not opened to recruitment.

Discussion

CATALYST is a nimble, accelerated, open-label, targeted phase II proof-of-principle multi-arm trial permitting efficient evaluation of repurposed and/or novel drugs to modify the disease progression of COVID-19 in patients admitted to wards and ICU. CATALYST aims to determine suitability of a proposed new treatment for evaluation in phase III national platform trials. CATALYST has an adaptive platform design, aiming to translate laboratory-based research to patients with SARS-CoV-2 infection without delay.

This trial is designed with clinical pressures caused by the pandemic in mind. Outcomes are easy to record, location-independent, and applicable across the spectrum of illness severity. We have also prioritised serial continuous measures over discontinuous or ordinal metrics, as these allow for greater statistical efficiency and thus smaller sample sizes. The primary outcome chosen provides a rapid, biologically-driven efficacy signal to allow early "go/no-go" decisions. While the WHO has adopted a consensus-based set of core outcome measures for studies of SARS-CoV-2 infection (29), our study aim was to develop a trial with a smaller sample size to provide earlier signals of potential efficacy for multiple investigational agents, allowing selection of the most promising to be taken forward by larger platforms with clinical outcomes.

We initially considered SpO_2/FiO_2 as a suitable primary outcome for CATALYST. This is an indicator of the severity, progression, or remission of acute lung injury (30). However, as real-world data emerged, we found the relationship between the ratio and outcome was complex and was also susceptible to measurement error in patients receiving ward-based forms of respiratory support. Furthermore, its prognostic utility is compromised if the inspired oxygen concentration is not rapidly adjusted to the patients' needs by the attending staff, which is often delayed in a pandemic. Other shortfalls included high variability between observations, the impact on the measure of switching the mode of oxygen delivery, and microthrombotic events altering SpO_2/FiO_2 through alteration of perfusion (31). We, therefore, evaluated whether CRP would be a better primary outcome.

C-Reactive Protein is produced as a homopentameric protein, termed native CRP (nCRP), which can irreversibly dissociate at sites of inflammation, tissue damage, and infection into five separate monomers, termed monomeric CRP (mCRP) (32). C-Reactive Protein levels are widely used as a marker of infection or inflammation; however, evidence suggests that CRP plays an active role in the inflammatory process (33-38).

During the first wave we modelled data on CRP over time in COVID-19 patients finding that it performed better than SpO_2/FiO_2. This is consistent with published data indicating that baseline and peak CRP, median CRP over time, slope of CRP rise over seven days, and rapid rise during early disease are all associated with outcome in hospitalised patients with COVID-19 (39-42). CRP trends over time tend to have greater predictive power, with change in CRP levels performing better at predicting respiratory failure and subsequent intubation than baseline CRP or respiratory rate-oxygenation (ROX) index (40). In addition, compared to those that die, patients who survive have lower peak CRP levels and earlier reductions (41). Notably, CRP at baseline also correlates with CT grading of lung involvement (43).

The recent success of dexamethasone in the treatment of COVID-19 reinforces the relevance of inflammatory pathology to clinical outcomes (44). Methylprednisolone has been associated with CRP reduction over seven days and improved clinical outcomes, in a strategy that continued this steroid until a target CRP, or the ratio of arterial oxygen partial pressure to fractional inspired oxygen (PiO_2/FiO_2) threshold, was reached (45). Although IL-6 blockade has a direct effect on CRP production, which might

For peer review only - http://bmjopen.bmj.com/site/about/guidelines.xhtml

obscure a relationship between CRP trends and outcome, small studies have suggested a differential effect on CRP decline between clinical responders and non-responders (46, 47).

While CRP may not show complete concordance with clinical outcomes, we argue that if dysregulated inflammation is indeed a key pathogenic driver in severe COVID-19, an immunomodulating drug capable of ameliorating those outcomes is also likely to show early improvement of CRP. Conversely, an immunomodulating drug unable to influence CRP is a less promising candidate to investigate in large phase III trials. Limitations of CRP, however, include lower utility for candidate therapeutics whose mechanism of action is not immunomodulation, and the diminished ability to assess drugs that directly target IL-6, due to the direct pharmacodynamics effects on CRP. This study was designed before use of IL-6 blockade outside of trials, and if tocilizumab were to be widely adopted, adaptive modification of the current trial design may be required to account for this.

In conclusion, the major strength of CATALYST is its ability to provide a rapid readout on safety, and proof-of-efficacy enabling phase III trial resources to be focussed and allocated for drugs with a high likelihood of success (44, 48). This will reduce the time lag in translating early phase drugs into effective therapeutics for COVID-19.

Ethics and dissemination

The trial will be performed in accordance with the recommendations guiding physicians in biomedical research involving human subjects, adopted by the 18th World Medical Association General Assembly, Helsinki, Finland and stated in the respective participating countries laws governing human research, and Good Clinical Practice. The initial protocol was approved by East Midlands - Nottingham 2 Research Ethics Committee, (REC Ref: 20/EM/0115) on 05-May-2020, with subsequent amendments approved on 28-May-2020 (addition of namilumab and infliximab), 12-Jun-2020 (inclusion change – suspected COVID-19), 20-Jun-2020 (following dexamethasone as standard of care use), 12-Oct-2020 (change of primary outcome to CRP). The MHRA has given its approval of all protocol versions; current version in use is 6.0.

A meeting will be held after the end of the trial to allow discussion of the main results among the collaborators prior to publication. Results of the primary and secondary endpoints will be submitted for publication in peer-reviewed journals.

Contributors

Study conception: TV, BAF, DS, AR, RS, DT, TW, MR, DP, DR, JB, PK, SG. Study design: TV, BAF, DS, AR, RS, DT, TW, MR, JS, DP, SJB, JSS, DR, JB, PK, SG. DS is the Trial Biostatistician and SG is the Senior Trial Biostatistician, both were responsible for developing the statistical plan.

Funding

This trial is supported by the Medical Research Council (MRC) grant number MC_PC_20007. SG is supported by a Senior Investigator Award from the National Institute of Health Research. Staff at the CRCTU are supported by core funding grants from Cancer Research UK (C22436/A25354), the NIHR Biomedical Research Centre (BRC-1215-20009), The Kennedy Trust for Rheumatology Research as part of the Arthritis – Trials Acceleration Programme (KENN161704), and Innovate UK as part of the Midlands – Wales Advanced Therapy Treatment Centres (104232). This paper presents independent research supported by the NIHR Birmingham Biomedical Research Centres at the University Hospitals Birmingham NHS Foundation Trust and the University of Birmingham, as well as Oxford and University College London Hospitals Biomedical Research Centres. The views expressed are those of the authors and not necessarily

For peer review only - http://bmjopen.bmj.com/site/about/guidelines.xhtml

those of the NHS, the NIHR or the Department of Health and Social Care. Namilumab is being provided free of charge by Izana Bioscience, Oxford, UK (now part of Roivant). Infliximab is being provided free of charge by Celltrion.

Competing interests

BAF has undertaken consultancy for Novartis, BMS, Servier, Galapagos and Janssen; MR is currently undertaking a Senior Clinical Fellowship financed by Roche; PK has undertaken consultancy for BMS, AstraZeneca, and AbbVie; all are unrelated to this trial. All other authors declare no competing interests.

Acknowledgements

We thank staff from the CRCTU, University of Birmingham including Dr Siân Lax for contributions to the paper.

References

1. Hall M, Pritchard M, Dankwa EA, Baillie JK, Carson G, Citarella BW, et al. ISARIC Clinical Data Report 20 November 2020. medRxiv. 2020:2020.07.17.20155218.

2. ICNARC Report on COVID-19 in Critcal Care: England, Wales and Northern Ireland, 29 January 2021 [Available from: https://www.icnarc.org/Our-Audit/Audits/Cmp/Reports.

3. Huang C, Huang L, Wang Y, Li X, Ren L, Gu X, et al. 6-month Consequences of COVID-19 in Patients Discharged from Hospital: A Cohort Study. The Lancet. 2021;397(10270):220-32.

4. Raman B, Cassar MP, Tunnicliffe EM, Filippini N, Griffanti L, Alfaro-Almagro F, et al. Medium-term Effects of SARS-CoV-2 Infection on Multiple Vital Organs, Exercise Capacity, Cognition, Quality of Life and Mental Health, Post-hospital Discharge. EClinicalMedicine. 2021;31:100683.

5. Ackermann M, Verleden SE, Kuehnel M, Haverich A, Welte T, Laenger F, et al. Pulmonary Vascular Endothelialitis, Thrombosis, and Angiogenesis in Covid-19. New England Journal of Medicine. 2020;383(2):120-8.

6. Leisman DE, Ronner L, Pinotti R, Taylor MD, Sinha P, Calfee CS, et al. Cytokine Elevation in Severe and Critical COVID-19: A Rapid Systematic Review, Meta-analysis, and Comparison with other Inflammatory Syndromes. The Lancet Respiratory Medicine. 2020;8(12):1233-44.

7. Huang C, Wang Y, Li X, Ren L, Zhao J, Hu Y, et al. Clinical Features of Patients Infected with 2019 Novel Coronavirus in Wuhan, China. The Lancet. 2020;395(10223):497-506.

8. Liao M, Liu Y, Yuan J, Wen Y, Xu G, Zhao J, et al. Single-cell Landscape of Bronchoalveolar Immune Cells in Patients with COVID-19. Nature Medicine. 2020;26(6):842-4.

9. Prompetchara E, Ketloy C, Palaga T. Immune Responses in COVID-19 and Potential Vaccines: Lessons Learned from SARS and MERS Epidemic. Asian Pacific Journal of Allergy and Immunology. 2020;38:1-9.

10. Randomised Evaluation of COVID-19 Therapy (RECOVERY) Trial Protocol 2020 [Available from: https://www.recoverytrial.net/for-site-staff.

11. Randomized, Embedded, Multifactorial Adaptive Platform trial for CommunityAcquired Pneumonia (REMAP-CAP): Core Protocol 2020 [Available from: https://www.remapcap.org/protocol-documents.

12. Dean NE, Gsell P-S, Brookmeyer R, Crawford FW, Donnelly CA, Ellenberg SS, et al. Creating a Framework for Conducting Randomized Clinical Trials during Disease Outbreaks. New England Journal of Medicine. 2020;382(14):1366-9.

13. Kieny MP, Salama P. WHO R&D Blueprint: a global coordination mechanism for R&D preparedness. Lancet. 2017;389(10088):2469-70.

For peer review only - http://bmjopen.bmj.com/site/about/guidelines.xhtml

14. Health Research Authority Consent and Participant Information Guidance [Available from: http://www.hra-decisiontools.org.uk/consent/principles-ALC-EnglandandWales.html.

15. Papp KA, Gooderham M, Jenkins R, Vender R, Szepietowski JC, Wagner T, et al. Granulocyte-macrophage Colony-stimulating Factor (GM-CSF) as a Therapeutic Target in Psoriasis: Randomized, Controlled Investigation using Namilumab, a Specific Human anti-GM-CSF Monoclonal Antibody. British Journal of Dermatology. 2019;180(6):1352-60.

16. Tanaka S, Harada S, Hiramatsu N, Nakaya R, Kawamura M. Randomized, double-blind, placebo-controlled, phase I study of the safety and pharmacokinetics of namilumab in healthy Japanese and Caucasian menInternational Journal of Clinical Pharmacology and Therapeutics. 2018;56(11):507-17.

17. Huizinga TW, Batalov A, Stoilov R, Lloyd E, Wagner T, Saurigny D, et al. Phase 1b Randomized, Double-blind Study of Namilumab, an anti-Granulocyte Macrophage Colony-stimulating Factor Monoclonal Antibody, in Mild-to-moderate Rheumatoid Arthritis. Arthritis Research & Therapy. 2017;19(1):53.

18. Taylor PC, Saurigny D, Vencovsky J, Takeuchi T, Nakamura T, Matsievskaia G, et al. Efficacy and Safety of Namilumab, a Human Monoclonal Antibody Against Granulocyte-macrophage Colony-stimulating Factor (GM-CSF) Ligand in Patients with Rheumatoid Arthritis (RA) with Either an Inadequate Response to Background Methotrexate Therapy or an Inadequate Response or Intolerance to an anti-TNF (Tumour Necrosis Factor) Biologic Therapy: A Randomized, Controlled Trial. Arthritis Research & Therapy. 2019;21(1):101.

19. Robinson PC, Richards D, Tanner HL, Feldmann M. Accumulating Evidence Suggests anti-TNF Therapy Needs to be Given Trial Priority in COVID-19 Treatment. The Lancet Rheumatology. 2020;2(11):e653-e5.

20. Williamson PR, Altman DG, Bagley H, Barnes KL, Blazeby JM, Brookes ST, et al. The COMET Handbook: version 1.0. Trials. 2017;18(3):280.

21. Core Outcome Measures in Effectiveness Trials [Available from: http://www.comet-initiative.org/.

22. Cancer Therapy Evaluation Program. Common Terminology Criteria for Adverse Events (CTCAE), Version 4.03. US Department of Health and Human Services, NIH, NCI. 2010.

23. ISARIC/WHO Clinical Characterisation Protocol for Severe Emerging Infections 2020 [Available from: https://isaric.tghn.org/clinical-characterisation-protocol-ccp/.

24. University of Birmingham Acute Care Research [Available from: https://www.birmingham.ac.uk/research/inflammation-ageing/acute-care-research.aspx.

25. National Institute for Health Research Biomedical Research Centres [Available from: https://www.nihr.ac.uk/explore-nihr/support/experimental-medicine.htm.

26. National Institute for Health Research Birmingham Biomedical Research Centre [Available from: https://www.birminghambrc.nihr.ac.uk/.

27. National Institute for Health Research Oxford Health Biomedical Research Centre [Available from: https://oxfordhealthbrc.nihr.ac.uk/.

28. National Institute for Health Research University College London Hospitals Biomedical Research Centre [Available from: https://www.uclhospitals.brc.nihr.ac.uk/content/biomedical-research-centre.

29. Marshall JC, Murthy S, Diaz J, Adhikari NK, Angus DC, Arabi YM, et al. A Minimal Common Outcome Measure Set for COVID-19 Clinical Research. The Lancet Infectious Diseases. 2020;20(8):e192-e7.

30. The ARDS Definition Task Force. Acute Respiratory Distress Syndrome: The Berlin Definition. JAMA. 2012;307(23):2526-33.

31. Vlaar APJ, de Bruin S, Busch M, Timmermans S, van Zeggeren IE, Koning R, et al. Anti-C5a Antibody IFX-1 (Vilobelimab) Treatment versus Best Supportive Care for Patients with Severe COVID-19 (PANAMO): An Exploratory, Open-label, Phase 2 Randomised Controlled Trial. The Lancet Rheumatology. 2020;2(12):e764-e73.

32. Sproston NR, Ashworth JJ. Role of C-Reactive Protein at Sites of Inflammation and Infection. Front Immunol. 2018;9:754-.

For peer review only

For peer review only - http://bmjopen.bmj.com/site/about/guidelines.xhtml

33. Thiele JR, Habersberger J, Braig D, Schmidt Y, Goerendt K, Maurer V, et al. Dissociation of Pentameric to Monomeric C-reactive Protein Localizes and Aggravates Inflammation: In Vivo Proof of a Powerful Proinflammatory Mechanism and a New Anti-inflammatory Strategy. Circulation. 2014;130(1):35-50.

34. Khreiss T, József L, Hossain S, Chan JS, Potempa LA, Filep JG. Loss of Pentameric Symmetry of C-reactive Protein is Associated with Delayed Apoptosis of Human Neutrophils. Journal of Biological Chemistry. 2002;277(43):40775-81.

35. Singh U, Devaraj S, Vasquez-Vivar J, Jialal I. C-reactive Protein Decreases Endothelial Nitric Oxide Synthase Activity via Uncoupling. Journal of Molecular and Cellular Cardiology. 2007;43(6):780-91.

36. Krayem I, Bazzi S, Karam M. The Combination of CRP Isoforms with oxLDL Decreases TNF-α and IL-6 Release by U937-derived Macrophages. Biomed Rep. 2017;7(3):272-6.

37. Khreiss T, József L, Potempa LA, Filep JG. Loss of Pentameric Symmetry in C-reactive Protein Induces Interleukin-8 Secretion Through Peroxynitrite Signaling in Human Neutrophils. Circulation Research. 2005;97(7):690-7.

38. Han KH, Hong KH, Park JH, Ko J, Kang DH, Choi KJ, et al. C-reactive Protein Promotes Monocyte Chemoattractant Protein-1 - Mediated Chemotaxis through Upregulating CC Chemokine Receptor 2 Expression in Human Monocytes. Circulation. 2004;109(21):2566-71.

39. Luo X, Zhou W, Yan X, Guo T, Wang B, Xia H, et al. Prognostic Value of C-Reactive Protein in Patients With Coronavirus 2019. Clinical Infectious Diseases. 2020;71(16):2174-9.

40. Mueller AA, Tamura T, Crowley CP, DeGrado JR, Haider H, Jezmir JL, et al. Inflammatory Biomarker Trends Predict Respiratory Decline in COVID-19 Patients. Cell Reports Medicine. 2020;1(8):100144.

41. Sharifpour M, Rangaraju S, Liu M, Alabyad D, Nahab FB, Creel-Bulos CM, et al. C-reactive Protein as a Prognostic Indicator in Hospitalized Patients with COVID-19. PLoS One. 2020;15(11):e0242400.

42. Malik P, Patel U, Mehta D, Patel N, Kelkar R, Akrmah M, et al. Biomarkers and Outcomes of COVID-19 Hospitalisations: Systematic Review and Meta-analysis. BMJ Evidence Based Medicine. 2020:doi: 10.1136/bmjebm-2020-111536.

43. Tan C, Huang Y, Shi F, Tan K, Ma Q, Chen Y, et al. C-reactive Protein Correlates with Computed Tomographic Findings and Predicts Severe COVID-19 Early. Journal of Medical Virology. 2020;92(7):856-62.

44. Horby P, Lim WS, Emberson JR, Mafham M, Bell JL, Linsell L, et al. Dexamethasone in Hospitalized Patients with Covid-19 - Preliminary Report. New England Journal of Medicine. 2020:doi: 10.1056/NEJMoa2021436.

45. Salton F, Confalonieri P, Meduri GU, Santus P, Harari S, Scala R, et al. Prolonged Low-Dose Methylprednisolone in Patients With Severe COVID-19 Pneumonia. Open Forum Infectious Diseases. 2020;7(10):ofaa421.

46. Montesarchio V, Parrela R, Iommelli C, Bianco A, Manzillo E, Fraganza F, et al. Outcomes and Biomarker Analyses Among Patients with COVID-19 Treated with Interleukin 6 (IL-6) Receptor Antagonist Sarilumab at a Single Institution in Italy. Journal for Immunotherapy of Cancer. 2020;8(2):e001089.

47. Jordan SC, Zakowski P, Tran HP, Smith EA, Gaultier C, Marks G, et al. Compassionate Use of Tocilizumab for Treatment of SARS-CoV-2 Pneumonia. Clinical Infectious Diseases. 2020;71(12):3168-73.

48. RECOVERY Collaborative Group. Azithromycin in Patients Admitted to Hospital with COVID-19 (RECOVERY): A Randomised, Controlled, Open-label, Platform Trial. The Lancet. 2021:doi: 10.1016/S0140-6736(21)00149-5.

For peer review only - http://bmjopen.bmj.com/site/about/guidelines.xhtml

Table 1 World Health Organisation Clinical Progression Scale

Patient State	Descriptor	Score
Uninfected	Uninfected; no viral RNA detected	0
Ambulatory	Asymptomatic; viral RNA detected	1
	Symptomatic; independent	2
	Symptomatic; assistance needed	3
Hospitalised; mild disease	Hospitalised; no oxygen therapy	4
	Hospitalised; oxygen by mask or nasal prongs	5
Hospitalised; severe disease	Hospitalised; oxygen by NIV or high flow	6
	Intubated and mechanical ventilation, $pO_2/FiO_2 \geq 150$ or $SpO_2/FiO_2 \geq 200$	7
	Mechanical ventilation $pO_2/FiO_2 < 150$ ($SpO_2/FiO_2 < 200$) **or** vasopressors	8
	Mechanical ventilation $pO_2/FiO_2 < 150$ ($SpO_2/FiO_2 < 200$) **and** vasopressors, dialysis or ECMO	9
Death	Dead	10

Adapted from Marshall et al., 2020 (29).

Footnotes for use in CATALYST

1. If pO_2 not available then use the SpO_2/FiO_2 ratio instead

2. For pO_2 measurements in kPa, use an online calculator e.g. https://www.msdmanuals.com/en-gb/medical-calculators/PaO2_FiO2Ratio.htm to calculate a pO_2/FiO_2 ratio equivalent to that obtained with pO_2 measured in mmHg, or else consider an equivalent ratio to 200, when dividing pO_2 in kPa by FiO_2, is 26.7, and an equivalent to 150 is 20.

3. If medically fit for discharge, record status as for ambulatory patient

4. Asymptomatic implies a return to baseline symptomatic state, i.e. no fever, and no cough, shortness of breath, confusion, myalgia, diarrhoea, fatigue, or weakness above what the participant would have experienced on a daily basis before their COVID-19 episode

5. Symptomatic but independent, implies that the participant has some of the additional symptoms as above, but needs no additional help with activities of daily living above what they required prior to their COVID-19 episode.

6. Symptomatic but needs assistance, implies that in addition to having symptoms as above, they require help with activities of daily living i.e. bathing/showering, personal hygiene and combing of hair, dressing, toileting, mobility/transferring and self-feeding, above what they required on a daily basis prior to their COVID-19 episode.

7. Score 0 (uninfected: no viral RNA detected) is not being assessed as part of CATALYST.

For peer review only - http://bmjopen.bmj.com/site/about/guidelines.xhtml

For peer review only

Figure Legends

Figure 1 CATALYST Trial Schema.

The gemtuzumab-ozogamicin arm has been de-prioritised and is not open to recruitment.

AE, adverse events; CRP, C-reactive protein; NEWS-2, National Early Warning Scale 2; WHO, World Health Organisation

For peer review only

For peer review only - http://bmjopen.bmj.com/site/about/guidelines.xhtml

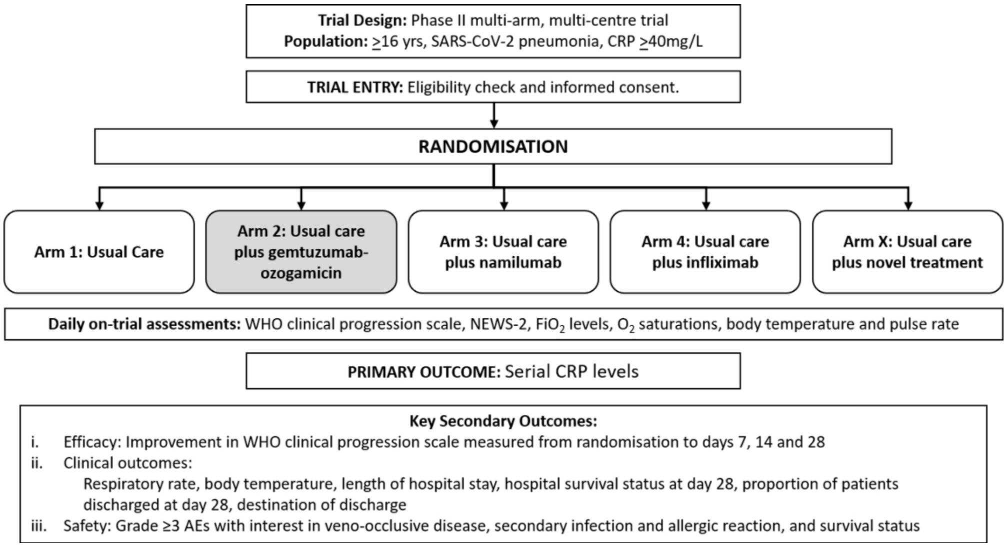

Figure 1 CATALYST Trial Schema. The gemtuzumab-ozogamicin arm has been de-prioritised and is not open to recruitment. AE, adverse events; CRP, C-reactive protein; NEW-2, National Early Warning Scale 2; WHO, World Health Organisation

203x109mm (300 x 300 DPI)

Supplementary Appendix 1 – CATALYST investigators

The CATALYST investigators include the following;

Chief Investigator Prof. Tonny Veenith (University Hospitals Birmingham), deputy Chief Investigator Dr Benjamin Fisher (University of Birmingham), Dr Francis Mussai (University of Birmingham), Prof Gary Middleton (University of Birmingham), Dr Dhruv Parekh (University of Birmingham), Dr Anna Rowe (Cancer Research UK Clinical Trials Unit (CRCTU), University of Birmingham), Prof. Duncan Richards (University of Oxford), Dr Mathew Rowland (University of Oxford), Prof. Julian Bion (University of Birmingham), Daniel Slade (CRCTU, University of Birmingham), Prof. Simon Gates (CRCTU, University of Birmingham), Prof. Pam Kearns (CRCTU, University of Birmingham), Dr Rowena Sharpe (CRCTU, University of Birmingham), Dr Sarah Bowden (CRCTU, University of Birmingham), Prof. David Thickett (University Hospitals Birmingham), Prof. Julian Bion (University of Birmingham), Dr Tony Whitehouse (University Hospitals Birmingham), Dr James Scriven (University Hospitals Birmingham), Dr Mansoor Bangash (University Hospitals Birmingham), Prof. Fang Gao-Smith (University Hospitals Birmingham), Dr Jaimin Patel (University Hospitals Birmingham), Prof. Elizabeth Sapey (University Hospitals Birmingham), Prof. Mark Coles (University of Oxford), Prof. Peter Watkinson (University of Oxford), Prof. Naj Rahman (University of Oxford), Prof. Ling-Pei Ho (University of Oxford), Prof. Brian Angus (University of Oxford), Dr Alex Mentzer (University of Oxford), Dr Alex Novak (University of Oxford), Prof. Marc Feldmann (University of Oxford).

CRCTU staff: Charlotte Gaskell, Camilla Bathurst, Joseph van de Wiel, Alex Vince, Lili Evans, Rhian Jones, Karan Kaliri, Susie Mee, Karen James, Bushra Rahman, Karen Turner.

Trial Steering Committee: Prof. Michael Matthay (Chair), Prof. Danny McAuley (Deputy Chair), Prof. Paul Dark, Prof. Sir Andrew McMichael, Andrew Hall (Independent Statistician), Simon Farrell (PPI representative), Hannah Farrell (PPI representative)

Independent Data Management Committee: Prof. Adam Hill (Chair), Prof. Christina Yap (Independent Statistician), Prof. Anthony Gordon.

Scientific Advisory Board: Prof. Philip Newsome (Chair; Birmingham BRC Deputy Director, Clinician), Prof. Helen McShane (Deputy Chair, Oxford BRC Director, Clinician), Prof. Graham Cooke (Imperial College BRC Representative, Clinician), Prof. Duncan Richards (Director of Oxford CTRU, Clinician), Prof. Bryan Williams (UCL BRC Director, Clinician), Prof. Ling-Pei Ho (Translational Research Collaborative Representative, Clinician), Prof. Tonny Veenith (Chief Investigator, Clinician), Dr Ben Fisher (Deputy Chief Investigator, Clinician), Prof. Vincenzo Libri (CRF Representative, Clinician), Prof. Julian Bion (Clinical Trials Oversight Committee Representative, Clinician), Prof. Pamela Kearns (Trial Sponsor Representative, Director of CRCTU, Clinician), Dr Rowena Sharpe (Trial Management Group Representative), Prof. Simon Gates (Senior Biostatistician, CRCTU), Dr Rebecca Turner (Statistician).

For peer review only - http://bmjopen.bmj.com/site/about/guidelines.xhtml

Supplementary Appendix 2 - Patient Information Sheets

<To be printed on hospital headed paper>

CATALYST - An early phase platform trial in (suspected) COVID-19

Which treatment could lessen the severity of a (suspected or confirmed) COVID-19 infection when compared with usual care in an NHS setting?

Patient Information Sheet

Invitation to take part

CATALYST is a clinical trial being led by doctors and researchers based at the University of Birmingham and University Hospitals Birmingham NHS Foundation Trust, and in collaboration with the University of Oxford. You do not have to take part. Taking part is entirely voluntary, and you should only participate if you want to. Choosing not to take part in this trial will not affect your care in any way.

Before you decide whether you want to take part, it is essential for you to understand why the research is being done and what your participation will involve.

Please take time to read the following information carefully and ask your doctor if there is anything that is not clear or if you would like more information.

The purpose of the trial

There is currently no vaccine and few effective treatments for COVID-19. As COVID-19 is a new illness, we are constantly learning more about how it affects the human body. We know that the COVID-19 virus affects a number of different cells in your body, including a type of blood cell called a macrophage (immune cell), and that it can cause the number of these cells to increase in your body. To fight an infection, your immune cells produce proteins called cytokines and chemokines. These proteins can cause inflammation and at high levels can lead to damage in the tissues and organs in your body. Researchers believe this is why some people with COVID-19 infection become very ill.

Therapies we are testing:

A new, unlicensed drug called namilumab which has been tested in patients with arthritis and other inflammatory conditions. It may reduce inflammation in the body caused by the coronavirus. It is currently being given to patients with COVID-19 in a clinical trial in Italy. Namilumab is being provided free of charge by Izana Bioscience Limited (now part of Roivant Sciences) for use in this trial.

For peer review only - http://bmjopen.bmj.com/site/about/guidelines.xhtml

A drug called infliximab (Remsima) which is widely used to treat arthritis and other conditions may reduce inflammation in the body caused by the coronavirus. Infliximab is being provided free of charge by Celltrion Healthcare UK Limited for use in this trial.

Once this inflammation has been reduced, it may be possible that your immune system will adapt and fight off the virus more effectively.

Infliximab has not been tested in patients with COVID-19, and namilumab has only been given to a limited number of patients in Italy, therefore testing these drugs is the purpose of this trial. If any of the drugs benefit people with a (suspected or confirmed) COVID-19 infection in this trial, the drug will then be included in another larger-scale clinical trial being conducted throughout the UK, which is designed to compare treatments to find out which is the best at treating this infection.

This is a national trial, and some hospitals running it are unable to offer patients all of the therapies available within the trial. Your doctor will tell you which treatments are available at your hospital, and if applicable, which ones are not available.

Why have I been invited?

You have been invited to take part in this trial because you are aged 16 years or older, you have been admitted to hospital, and your doctor believes you have a COVID -19 infection and you have higher levels of C-reactive protein in your blood than normal (this increases when there's an infection or inflammation in your body).

What will happen to me if I take part?

- You will be agreeing to take part in a clinical trial and will be actively monitored for 28 days and longer if necessary.
- This clinical trial has three treatment groups or 'arms': one for each of the treatments listed above, and the other arm is 'usual care'. Usual care means that you will receive exactly the same treatment that you would receive in the hospital whether you decide to take part in the trial or not.
- The treatment you will receive is randomly allocated (chosen by chance) by a computer. Neither you nor your doctor will be able to choose the treatment you receive. Your research doctor or nurse will inform you of which treatment you will receive once they have entered you into the trial.
- If you are allocated the 'usual care' arm, you will receive the same medical care as all other patients being treated for a (suspected or confirmed) COVID-19 infection.

For peer review only - http://bmjopen.bmj.com/site/about/guidelines.xhtml

- If you are allocated to receive namilumab, you will receive this in addition to the usual medical care received by patients who have this disease. Namilumab will be given to you through a drip in a vein, usually in your arm, on one occasion only.
- If you are allocated to infliximab, you will receive this in addition to the usual medical care received by patients who have this disease. Infliximab will be given to you through a drip in a vein, usually in your arm, on one occasion only.
- You will have routine blood samples whilst you are in the hospital as part of 'usual care.' If you are allocated to the namilumab or infliximab arm, you may have more blood samples taken regularly before and during treatment to check it is safe for you to receive treatment.
- If you are a female of child bearing potential, a pregnancy test will be performed as part of the screening tests.
- We would also like your permission to collect samples for research. The collection of these samples is optional. This will only take place at certain hospitals. Your doctor will tell you if research samples are being collected at your hospital. This includes collecting samples of blood, and may include swabs from your nose and throat, and if you are placed on a ventilator, samples of the secretions from the tube placed in your windpipe to help you breathe may be taken. Samples will be collected on up to three separate occasions. These samples will be used in laboratory studies to gain a better understanding of COVID-19 and how patients respond to treatment.
- While you are in hospital your health and wellbeing will be monitored in accordance with usual care.

What are the potential benefits of taking part?

This research has been designed to help develop treatment for future patients with suspected or confirmed COVID-19 infection. It is important to understand that you may not directly benefit from taking part in this trial. The benefits for you as an individual are unknown.

What are the possible disadvantages and risks of taking part?

This trial is testing a new way of treating suspected or confirmed COVID-19 infection and you may have side effects from the treatment while taking part in the trial.

All of the treatments have been given to humans before but not in suspected or confirmed COVID-19 infection so there may be side effects that your doctors are not currently aware of, and these may be serious because of this an independent committee will be monitoring the safety of the treatments in this trial on a regular basis.

Everyone taking part in the trial will be monitored carefully for side effects. However, the doctors do not know all the side effects that may occur and we don't know how the drugs used in this trial will interact with the

For peer review only - http://bmjopen.bmj.com/site/about/guidelines.xhtml

other drugs being used to treat (suspected or confirmed) COVID-19. Side effects may be mild or serious or may even be life-threatening. The doctors may give you medicines to help lessen side effects or the trial treatment may be postponed or stopped, depending on the side-effects they may experience.

Specific information is provided below for each of the trial treatments available in the trial is included below:

Namilumab

Namilumab is a new drug that is being tested to treat diseases that cause inflammation such as rheumatoid arthritis.

The side effects that are known so far are:

- Low neutrophil count (a type of white blood cell)
- Minor symptoms such as runny nose and headache
- Tachycardia (a fast heart beat)*

* A short change in heart rhythm was seen with no apparent harm in one person receiving namilumab in another trial.

Infliximab

Infliximab has been widely used for rheumatoid arthritis and other conditions for 20 years but it has some potential side effects. It has been used to treat patients with sepsis (serious infection) before on intensive care units and was demonstrated to be safe. The more common known side effects of infliximab when used to treat other conditions such as arthritis have been summarised below:

While you are receiving Infliximab:

Common side effects (experienced by 1 in 100 people) include:

- Allergic reaction: this can be mild but is sometimes severe and may even be life-threatening. For this reason you will be very carefully monitored while receiving the infliximab infusion and for the period afterwards
- Feeling and being sick
- Headache
- Flu-like symptoms

During the days or weeks receiving Infliximab:

Very common side effects (experienced by 1 in 10 people) include:

- Increased risk of infection. Infliximab can interfere with the body's ability to fight other infections caused by bacteria, fungi, other viruses such as hepatitis B, and tuberculosis. You will be monitored

For peer review only - http://bmjopen.bmj.com/site/about/guidelines.xhtml

carefully for signs of infection other than COVID-19 and be offered appropriate treatment if another infection is detected.

<u>*Common side effects (experienced by between 1 in 10 to 1 in 100 people) include:*</u>

- Abnormal liver function tests. Blood tests will be done frequently to check that your liver is working properly.
- Some patients may experience worsening of psoriasis

Pregnancy and Breast Feeding

There is very little known about the effects of namilumab on an unborn baby and there is some information available about the infliximab on an unborn baby. As a precaution, women who are breast feeding are excluded from the trial and women of child bearing potential must have a negative pregnancy test prior to startng the trial. It is important that if you receive a trial treatment that you use adequate birth control if you (or your partner if you are male) are of child bearing potential. For namilumab, male and female participants should use effective contraception for 18 weeks after the last dose of drug. For infliximab, male and female participants should use contraception for 26 weeks after the last dose of drug.

Effective contraception is a method that has a failure rate of less than 1% a year when used correctly and all the time. Examples of these include:

- combined (oestrogen and progesterone containing) hormonal contraception e.g. the "pill", patch
- progesterone only contraception (includes the "mini-pill", injection, implant)
- Intrauterine device (IUD) or hormone-releasing system (IUS)
- vasectomised partner
- sexual abstinence

Recent guidelines in rheumatology patients suggests infliximab can be given to patients up until the 16th week of pregnancy. However, as this is the first time infliximab is being used in suspected or confirmed COVID-19 patients it is important that you understand the contraception information above.

It is also important that you do not breastfeed for 6 months after the last dose of namilumab or infliximab.

Is there any prohibited medication whilst I am on the trial?

Your trial doctor will look at the medicines that you are already taking and let you know whether you are able to still take them while you are taking part in the trial, or whether you would need to stop any of them.

For peer review only - http://bmjopen.bmj.com/site/about/guidelines.xhtml

What will happen if I don't want to carry on with the trial?

You can withdraw from the trial at any time without this having any effect on your medical care, however all information and blood samples already collected from your time on the trial will still be used.

Will my taking part in this trial be kept confidential?

All information collected about you for this trial will be subject to the General Data Protection Regulation 2018 and Data Protection Act 2018 for Health and Care Research and will be kept strictly confidential.

The University of Birmingham is the Sponsor for this trial. The University of Birmingham will be using information from your medical records in order to undertake this trial and will act as the data controller. This means that the University of Birmingham are responsible for looking after your information and using it properly. University of Birmingham and the NHS will keep identifiable information about you for at least 10 years after the trial has finished; this allows the results of the trial to be verified if needed.

In the Trial Office you will be identified by a unique trial number. In routine communication between your hospital and the Trial Office you will only be identified by trial number, initials and date of birth. Information about you, your health and wellbeing may be provided to the Trial Office on paper or electronically. We would also like to collect your NHS Number. This will allow researchers to collect information about your health and wellbeing from national records (e.g. Office for National Statistics, NHS Central Registries or other registries including those managed by NHS Digital) after the trial has ended. This will help us to determine the long-term impact of the trial treatment and COVID-19 on people's health.

By taking part in the trial you will be agreeing to allow research staff from the Trial Office to look at the trial records, including your medical records. It may be necessary to allow authorised personnel from government regulatory agencies (e.g. Medicines and Healthcare products Regulatory Agency (MHRA)), the Sponsor and/or NHS bodies to have access to information about you. This is to ensure that the trial is being conducted to the highest possible standards.

If you are randomised to receive namilumab or infliximab, pseudo-anonymised data from the trial may also be provided to the pharmaceutical company who is providing the drug you are given for safety monitoring or licensing purposes, where applicable. This is for your and others' protection to track the safety of the trial treatment. This may involve sending data outside of the United Kingdom to a European country of the United States of America. Your name and any identifying details (such as date of birth) will not be given to any of these parties.

We may be asked to share the trial information (data) we have collected with researchers running other studies, so that they can perform analysis on the data to answer other important questions about COVID-19.

For peer review only - http://bmjopen.bmj.com/site/about/guidelines.xhtml

These other researchers may be based in universities, NHS organisations, companies involved in health research and may be in this country or abroad. Non-identifiable summary information may also be shared with COVID-19 related UK government departments. Any such request is carefully considered by the trial researchers and will only be granted if the research is of high scientific standard and the necessary procedures and approvals are in place. The information will only be used for the purpose of health research and cannot be used to contact you or to affect your care. It will not be used to make decisions about future services available to you, such as insurance.

All individuals who have access to your information have a duty of confidentiality to you. Under no circumstances will you be identified in any way in any report, presentation or publication arising from this or any other research study.

You can withdraw your consent to our processing any more of your data at any time. Your rights to access change or move your information are limited, as we need to manage your information in specific ways in order for the research to be reliable and accurate. If you withdraw from the trial, we will keep the information about you and the samples that we have already obtained. Under the provisions of the General Data Protection Regulation 2018 and Data Protection Act 2018 you have the right to know what information the University of Birmingham has recorded about you. If you wish to view this information or find more about how we use this information, please contact Legal Services at Legal Services, University of Birmingham, Edgbaston, Birmingham, B15 2TT.

What will happen to the samples I give?

If you agree to take part in the optional research sample collection, the samples will be sent to laboratories based in the Universities of Birmingham or Oxford for storage and use in laboratory based research for the trial. If you give your consent, any samples leftover at the end of analysis for the trial, may be kept for future ethically approved research. These laboratory-based research projects will include genetic analysis of these samples. This DNA and RNA analysis is for scientific purposes and is not expected to provide findings of any clinical significance for you or your relatives, so the results will not be fed back to you. It is difficult to predict exactly what scientific developments there may be so we cannot give precise details of what research might be done.

No one using your samples for laboratory research will have access to your personal details. The samples sent to the University laboratories will be identified by your unique trial number only. All samples relating to you will be stored in accordance with the Human Tissue Act 2004.

For peer review only - http://bmjopen.bmj.com/site/about/guidelines.xhtml

What will happen to the results of the research trial?

At the end of the trial, the findings will be published in peer-reviewed medical and scientific journals. These publications will be available upon request from your trial doctor. We will also make a lay summary of the result available on the trial websites.

Who is organising and funding the research?

The trial is sponsored and being undertaken by the University of Birmingham in collaboration with University Hospitals Birmingham NHS Foundation Trust. The trial is being coordinated by the Cancer Research UK Clinical Trials Unit (CRCTU) within the University of Birmingham.

The trial is funded by an educational grant from UK Research and Innovation and drugs are being provided free of charge by the pharmaceutical companies.

Who has reviewed the trial?

All research in the NHS is looked at by an independent group of people called a Research Ethics Committee to protect your safety, rights, wellbeing and dignity. This trial has been reviewed and given favourable opinion by the East Midlands-Nottingham 2 Research Ethics Committee and by the NHS Health Research Authority. While the trial is ongoing the results will be reviewed regularly by an independent Data Monitoring Committee to ensure that it is appropriate to continue with the trial.

Expenses and Payments

As you will already be an inpatient in the hospital during the course of the trial you will not have to make any extra visits to participate in the trial and therefore will incur no additional expenses.

What if there is a problem?

If you have a concern about any aspect of this trial, you should ask to speak with your trial doctor who will do their best to answer your questions (see contact number at the end of this form). If you remain unhappy and wish to complain formally, you can do this through your hospital's Patient Advice and Liaison Services (PALS); they can be contacted by:

(Insert local contact details).

If you are harmed and this is due to someone's negligence then you may have grounds for legal action for compensation against the Sponsor of the trial (University of Birmingham) or the NHS Trust but you may have to pay your legal costs. NHS Trust Hospitals have a duty of care to all patients treated, whether or not the patient is taking part in a clinical trial, and the normal NHS complaints mechanisms will still be available to you (if appropriate).

For peer review only - http://bmjopen.bmj.com/site/about/guidelines.xhtml

Further information and contact details

Trial Doctor: ____

Research Nurse: ____

☎: ______________________ Emergency (24 hours) ☎: ______________________

For peer review only

For peer review only - http://bmjopen.bmj.com/site/about/guidelines.xhtml

<To be printed on hospital headed paper>

CATALYST - An early phase platform trial in (suspected or confirmed) COVID-19

Which treatment could lessen the severity of a (suspected or confirmed) COVID-19 infection when compared with usual care in an NHS setting?

Personal Legal Representative Information Sheet

For peer review only

Invitation to take part

CATALYST is a clinical trial being led by the University of Birmingham and University Hospitals Birmingham NHS Foundation Trust in collaboration with the University of Oxford. You are being asked to assess the information contained within this information sheet on the patient's behalf, and make a decision based on the presumed wish of the patient on whether they would like to take part in the trial or not. The decision must be based on the patient's presumed will, and not based on your own views or objection.

Taking part is entirely voluntary, and choosing not to take part in this trial will not affect his/ her care in any way.

Before you decide whether the patient would want to take part, it is essential for you to understand why the research is being done and what his/her participation will involve. For this reason, the information contained within this personal legal representative information sheet is identical to that contained within the patient information sheet. The patient information sheet will be presented to the patient should he/ she clinically improve to the point of being able to assess the information to make an informed decision on whether to continue to take part in the trial or not.

Please take time to read the following information carefully.

The purpose of the trial

There is currently no vaccine and few effective treatments for COVID-19. As COVID-19 is a new illness, we are continually learning more about how it affects the human body. We know that the COVID-19 virus affects a number of different cells in the body, including a type of blood cell called a macrophage (immune cell), and that it can cause the number of these cells to increase in the body. To fight an infection, immune cells produce proteins called cytokines and chemokines. These proteins can cause inflammation and at high levels can lead to

For peer review only - http://bmjopen.bmj.com/site/about/guidelines.xhtml

damage in the tissues and organs in the body. Researchers believe this is why some people with COVID-19 infection become very ill.

Therapies we are testing:

A new, unlicensed drug called namilumab which has been tested in patients with arthritis and other inflammatory conditions. It may reduce inflammation in the body caused by the coronavirus. It is currently being given to patients with COVID-19 in a clinical trial in Italy. Namilumab is being provided free of charge by Izana Bioscience Limited (now part of Roivant Sciences) for use in this trial.

A drug called infliximab (Remsima) which is widely used to treat arthritis and other conditions may reduce inflammation in the body caused by the coronavirus. Infliximab is being provided free of charge by Celltrion Healthcare UK Limited for use in this trial.

Once this inflammation has been reduced, it may be possible that the immune system will adapt and fight off the virus more effectively.

Infliximab has not been tested in patients with COVID-19, and nalimumab has only been given to a limited number of patients in Italy. Therefore, the purpose of this trial is to assess if these drugs may be helpful in treating COVID-19. If in this trial any drug shows an initial sign of benefit in (suspected or confirmed) COVID-19, then it will be included in another larger-scale clinical trial being conducted throughout the UK, which is designed to compare treatments to find out which is the best at treating this infection.

This is a national trial and some hospitals running this trial are unable to offer patients all of the therapies available within this trial. The doctor will tell you which treatments are available at the hospital, and if applicable, which ones are not available.

Why has my relative/ friend been invited?

Your relative/ friend has been invited to take part in this trial because they are aged 16 years or older, they have been admitted to hospital, and their doctor believes they have either a suspected or confirmed COVID -19 infection or have tested positive with a COVID-19 infection and they have higher levels of C-reactive protein in their blood than normal (this increases when there's an infection or inflammation in your body).

What will happen to my relative/ friend if they take part?

For peer review only - http://bmjopen.bmj.com/site/about/guidelines.xhtml

- You will agree for your relative/ friend to take part in a clinical trial, and they will be actively monitored for 28 days and longer if necessary.
- This clinical trial has three treatment groups or ‘arms’: one for each of the treatments listed above, and the other arm is ‘usual care’. Usual care means that they will receive the same treatment that everyone with a (suspected or confirmed) COVID-19 infection would get in the hospital whether you decide your relative/ friend takes part in the trial or not.
- The treatment they will receive is randomly allocated (chosen by chance) by a computer. Neither you nor your relative’s/ friend’s doctor will be able to select the treatment your relative/ friend receives. The research doctor or nurse will inform you of which treatment your relative/ friend will receive once they have entered them into the trial.
- If your relative/ friend is allocated the ‘usual care’ arm, they will receive the same medical care as all other patients being treated for a (suspected or confirmed) COVID-19 infection.
- If your relative/ friend is allocated to receive namilumab, they will get this in addition to the usual medical care received by patients who have this disease. Namilumab will be given to your relative/ friend through a drip in a vein, usually in their arm, on one occasion only.
- If your relative/ friend is allocated to receive infliximab, they will get this in addition to the usual medical care received by patients who have this disease. Infliximab will be given to your relative/ friend through a drip in a vein, usually in their arm, on one occasion only.
- Your relative/ friend will have routine blood samples while they are in the hospital as part of ‘usual care.’ If your relative/ friend is allocated to the namilumab or infliximab arm, they may have more blood samples taken regularly before and during treatment to check it is safe for them to receive treatment.
- If your relative/ friend are a female of childbearing potential, a pregnancy test will be performed as part of the screening tests.
- We would also like your permission to collect samples from your relative/ friend for research. The collection of these samples is optional. **This will only take place at certain hospitals**. The doctor will tell you if research samples are being collected at your relative/ friend’s hospital. This includes collecting samples of blood, and may include swabs from your relative/ friend’s nose and throat, and if they are placed on a ventilator, samples of the secretions from the tube placed in their windpipe to help them breathe may be taken. Samples will be collected on up to three separate occasions. These samples will be used in laboratory studies to gain a better understanding of COVID-19 and how patients respond to treatment.
- While your relative/ friend is in hospital, their health and wellbeing will be monitored in accordance with usual care.

For peer review only - http://bmjopen.bmj.com/site/about/guidelines.xhtml

What are the potential benefits of taking part?

This research has been designed to help develop a treatment for future patients with (suspected or confirmed) COVID-19 infection. It is important to understand that your relative/ friend may not directly benefit from taking part in this trial. The benefits for them as an individual are unknown.

What are the possible disadvantages and risks of taking part?

Your relative/ friend may have side effects from the treatment while taking part in the trial. This trial is testing a new way of treating (suspected or confirmed) COVID-19 infection.

All of the treatments have been given to humans before but not in (suspected or confirmed) COVID-19 infection, so there may be side effects that the doctors are not currently aware of, and these may be serious; because of this an independent committee will be monitoring the safety of the treatments in this trial regularly.

Everyone taking part in the trial will be monitored carefully for side effects. However, the doctors do not know all the side effects that may occur and we don't know how the drugs used in this trial will interact with the other drugs being used to treat (suspected or confirmed) COVID-19. Side effects may be mild or serious or may even be life-threatening. The doctors may give your relative/ friend medicines to help lessen side effects or the trial treatment may be postponed or stopped, depending on the side-effects they may experience.

Specific information is provided below for each of the trial treatments available in the trial is included below:

Namilumab

Namilumab is a new drug that is being tested to treat diseases that cause inflammation such as rheumatoid arthritis.

The side effects that are known so far are:

- Low neutrophil count (a type of white blood cell)
- Minor symptoms such as runny nose and headache
- Tachycardia (a fast heart beat)*

* A short change in heart rhythm was seen with no apparent harm in one person receiving namilumab in another trial.

Infliximab

Infliximab has been widely used for rheumatoid arthritis and other conditions for 20 years but it has some potential side effects. It has been used to treat patients with sepsis (serious infection) before on intensive care units and was demonstrated to be safe. The more common known side effects of infliximab when used to treat other conditions such as arthritis have been summarised below:

For peer review only - http://bmjopen.bmj.com/site/about/guidelines.xhtml

While your relative/ friend is receiving Infliximab:

- Common side effects (experienced by 1 in 100 people) include:
- Allergic reaction: this can be mild but is sometimes severe and may even be life-threatening. For this reason your relative/ friend will be very carefully monitored while receiving the iInfliximab infusion and for the period afterwards
- Feeling and being sick
- Headache
- Flu-like symptoms

During the days or weeks receiving Infliximab:

Very common side effects (experienced by 1 in 10 people) include:

- Increased risk of infection. Infliximab can interfere with the body's ability to fight other infections caused by bacteria, fungi, other viruses such as hepatitis B, and tuberculosis. Your relative/ friend will be monitored carefully for signs of infection other than COVID-19 and be offered appropriate treatment if another infection is detected.

Common side effects (experienced by between 1 in 10 to 1 in 100 people) include:

- Abnormal liver function tests. Blood tests will be done frequently to check that your relative/ friend's liver is working properly.
- Some patients may experience worsening of psoriasis

Pregnancy and Breast Feeding

There is very little known about the effects of namilumab on an unborn baby and there is some information available about the and infliximab on an unborn baby,. As a precaution, women who are breast feeding are excluded from the trial and women of child bearing potential must have a negative pregnancy test prior to startng the trial. It is important that if your relative/ friend receives a trial treatment that they use adequate birth control if they (or their partner if they are male) are of child bearing potential. For namilumab, male and female participants should use effective contraception for 18 weeks after the last dose of drug. For infliximab, male and female participants should use contraception for 26 weeks after the last dose of drug.

Effective contraception is a method that has a failure rate of less than 1% a year when used correctly and all the time. Examples of these include:

- combined (oestrogen and progesterone containing) hormonal contraception e.g. the "pill", patch
- progesterone only contraception (includes the "mini-pill", injection, implant)
- Intrauterine device (IUD) or hormone-releasing system (IUS)
- vasectomised partner

For peer review only - http://bmjopen.bmj.com/site/about/guidelines.xhtml

- sexual abstinence

Recent guidelines in rheumatology patients suggests infliximab can be given to female patients up until the 16th week of pregnancy. However, as this is the first time infliximab is being used in (suspected or confirmed) COVID-19 patients it is important your relative/ friend understands the contraception information above.
It is also important that any female patients do not breastfeed for 6 months after the last dose of namilumab or infliximab.

Is there any prohibited medication whilst my relative/ friend is on the trial?

Your relative/ friend's trial doctor will look at the medicines that they are already taking and let you/ your relative/ friend know whether they are able to still take them while they are taking part in the trial, or whether they would need to stop any of them.

What will happen if I don't want my relative/ friend to carry on with the trial?

You can withdraw your relative/ friend from the trial at any time without this having any effect on their medical care, however, all information and blood samples already collected from their time on the trial will still be used.

Will their taking part in this trial be kept confidential?

All information collected about your relative/ friend for this trial will be subject to the General Data Protection Regulation 2018 and Data Protection Act 2018 for Health and Care Research and will be kept strictly confidential.

The University of Birmingham is the Sponsor for this trial. The University of Birmingham will be using information from your relative/ friend's medical records in order to undertake this trial and will act as the data controller. This means that the University of Birmingham are responsible for looking after your relative/ friend's information and using it properly. The University of Birmingham and the NHS will keep identifiable information about them for at least ten years after the trial has finished; this allows the results of the trial to be verified if needed.

In the Trial Office, your relative/ friend will be identified by a unique trial number. In routine communication between the hospital and the Trial Office, your relative/ friend will only be identified by trial number, initials and date of birth. Information about your relative/ friend, their health and wellbeing may be provided to the Trial Office on paper or electronically. We would also like to collect your relative/ friend's NHS Number. This will allow researchers to collect information about their health and wellbeing from national records (e.g. Office for National Statistics, NHS Central Registries or other registries including those managed by NHS Digital) after the

For peer review only - http://bmjopen.bmj.com/site/about/guidelines.xhtml

trial has ended. This will help us to determine the long-term impact of the trial treatment and COVID-19 on people's health.

By taking part in the trial, you will agree to allow research staff from the Trial Office to look at the trial records, including the medical records of your relative/ friend. It may be necessary to allow authorised personnel from government regulatory agencies (e.g. Medicines and Healthcare products Regulatory Agency (MHRA)), the Sponsor and/or NHS bodies to have access to information about your relative/ friend. This is to ensure that the trial is being conducted to the highest possible standards.

If the your relative/ friend is randomised to receive namilumab or infliximab, pseudo-anonymised data from the trial may also be provided to the pharmaceutical company who is providing the drug they are given for safety monitoring or licensing purposes, where applicable. This is for their and others' protection to track the safety of the trial treatment. This may involve sending data outside of the United Kingdom to a European country of the United States of America. Your relative/ friend's name and any identifying details (such as date of birth) will not be given to any of these parties.

We may be asked to share the trial information (data) we have collected with researchers running other studies so that they can perform analysis on the data to answer other important questions about COVID-19. These other researchers may be based in universities, NHS organisations companies involved in health research and maybe in this country or abroad. Non-identifiable summary information may also be shared with COVID-19 related UK government departments. Any such request is carefully considered by the trial researchers and will only be granted if the research is of high scientific standard and the necessary procedures and approvals are in place. The information will only be used for the purpose of health research and cannot be used to contact your relative/ friend or affect their care. It will not be used to make decisions about future services available to them, such as insurance.

All individuals who have access to your relative/ friend's information have a duty of confidentiality to them. Under no circumstances will they be identified in any way in any report, presentation or publication arising from this or any other research study.

You can withdraw your consent to our processing any more of your relative/ friend's data at any time. Your rights to access change or move your relative/ friend's information are limited, as we need to manage their information in specific ways in order for the research to be reliable and accurate. If you withdraw your relative/ friend from the trial, we will keep the information about them and the samples that we have already obtained. Under the provisions of the General Data Protection Regulation 2018 and Data Protection Act 2018, your relative/ friend has the right to know what information the University of Birmingham has recorded about them.

For peer review only - http://bmjopen.bmj.com/site/about/guidelines.xhtml

If your relative/ friend wishes to view this information or find more about how we use this information, please contact Legal Services at Legal Services, University of Birmingham, Edgbaston, Birmingham, B15 2TT.

What will happen to the samples they give?

If you agree for your relative/ friend to take part in the optional research sample collection, the samples will be sent to laboratories based in the Universities of Birmingham or Oxford for storage and use in laboratory-based research for the trial. If you give your consent, any samples leftover at the end of analysis for the trial, may be kept for future ethically approved research. This laboratory-based research will include genetic analysis of these samples. This DNA and RNA analysis is for scientific purposes and is not expected to provide findings of any clinical significance for your relative/ friend or their relatives, so the results will not be fed back to your relative/ friend. It is difficult to predict precisely what scientific developments there may be so we cannot give precise details of what research might be done.

No one using your relative/ friend's samples for laboratory research will have access to their personal details. The samples sent to the University laboratories will be identified by your relative/ friend's unique trial number only. All samples relating to them will be stored in accordance with the Human Tissue Act 2004.

What will happen to the results of the research trial?

At the end of the trial, the findings will be published in in peer-reviewed medical and scientific journals. These publications will be available upon request from your relative/ friend's trial doctor. We will also make a lay summary of the result available on the trial websites.

Who is organising and funding the research?

The trial is sponsored and being undertaken by the University of Birmingham in collaboration with University Hospitals Birmingham NHS Foundation Trust . The trial is being coordinated by the Cancer Research UK Clinical Trials Unit (CRCTU) within the University of Birmingham.

The trial is funded by an educational grant from UK Research and Innovation and drugs are being provided free of charge by the pharmaceutical companies.

Who has reviewed the trial?

All research in the NHS is looked at by an independent group of people called a Research Ethics Committee to protect your relative/ friend's safety, rights, wellbeing and dignity. This trial has been reviewed and given favourable opinion by the East Midlands-Nottingham 2 Research Ethics Committee and by the NHS Health Research Authority. While the trial is ongoing the results will be reviewed regularly by an independent Data Monitoring Committee to ensure that it is appropriate to continue with the trial.

For peer review only - http://bmjopen.bmj.com/site/about/guidelines.xhtml

Expenses and Payments

As your relative/ friend will already be an inpatient in the hospital during the trial they will not have to make any extra visits to participate in the trial and therefore will incur no additional expenses.

What if there is a problem?

If you have a concern about any aspect of this trial, you should ask to speak with your relative/ friend's trial doctor who will do their best to answer your questions (see contact number at the end of this form). If you remain unhappy and wish to complain formally, you can do this through your relative/ friend's hospital's Patient Advice and Liaison Services (PALS); they can be contacted by:

(Insert local contact details).

If your relative/ friend is harmed and this is due to someone's negligence then your relative/ friend may have grounds for legal action for compensation against the Sponsor of the trial (University of Birmingham) or the NHS Trust but they may have to pay their legal costs. NHS Trust Hospitals have a duty of care to all patients treated, whether or not the patient is taking part in a clinical trial, and the normal NHS complaints mechanisms will still be available to your relative/ friend (if appropriate).

Further information and contact details

Trial Doctor: ____

Research Nurse: ____

☎: _________________________ Emergency (24 hours) ☎: _________________________

For peer review only - http://bmjopen.bmj.com/site/about/guidelines.xhtml

<To be printed on hospital headed paper>

CATALYST - An early phase platform trial in (suspected or confirmed) COVID-19

Which treatment could lessen the severity of a (suspected or confirmed) COVID-19 infection when compared with usual care in an NHS setting?

Professional Legal Representative Information Sheet

For peer review only

Invitation to take part

CATALYST is a clinical trial being led by the University of Birmingham and University Hospitals Birmingham NHS Foundation Trustand in collaboration with the University of Oxford. You are being asked to consent on the basis of the presumed will of the patient by assessing the information contained within this information sheet. This applies both to the wish of the patient to take part, or to refuse to take part. Taking part is entirely voluntary, and choosing not to take part in this trial will not affect his/ her care in any way.

Before you decide whether the patient would want to take part, it is essential for you to understand why the research is being done and what his/her participation will involve. For this reason, the information contained within this professional legal information sheet is identical to that contained within the patient information sheet. The patient information sheet will be presented to the patient should he/ she clinically improve to the point of being able to assess the information in order to make an informed decision on whether to continue to take part in the trial or not.

Please take time to read the following information carefully.

The purpose of the trial

There is currently no vaccine and few effective treatments for COVID-19. As COVID-19 is a new illness, we are constantly learning more about how it affects the human body. We know that the COVID-19 virus affects several different cells in the body, including a type of blood cell called a macrophage (immune cell), and that it can cause the number of these cells to increase in the body. To fight an infection, immune cells produce proteins called cytokines and chemokines. These proteins can cause inflammation and at high levels can lead to damage in the tissues and organs in the body. Researchers believe this is why some people with COVID-19 infection become very ill.

For peer review only - http://bmjopen.bmj.com/site/about/guidelines.xhtml

Therapies we are testing:

A new, unlicensed drug called namilumab which has been tested in patients with arthritis and other inflammatory conditions. It may reduce inflammation in the body caused by the coronavirus. It is currently being given to patients with COVID-19 in a clinical trial in Italy. Namilumab is being provided free of charge by Izana Bioscience Limited (now part of Roivant Sciences) for use in this trial.

A drug called Infliximab (Remsima) which is widely used to treat arthritis and other conditions may reduce inflammation in the body caused by the coronavirus. Infliximab is being provided free of charge by Celltrion Healthcare UK Limited for use in this trial.

Once this inflammation has been reduced, it may be possible that the immune system will adapt and fight off the virus more effectively.

Infliximab has not been tested in patients with COVID-19, and namilumab has only been given to a limited number of patients in Italy. Therefore, the purpose of this trial is to assess if these drugs may be helpful in treating COVID-19. If any of the drugs benefit people with a (suspected or confirmed) COVID-19 infection in this trial, the drug will be included in another larger-scale clinical trial being conducted throughout the UK, which is designed to compare treatments to find out which is the best at treating this infection.

This is a national trial, and some hospitals running this trial are unable to offer patients all of the therapies available within this trial. The trial doctor will tell you which treatments are available at your hospital, and if applicable, which ones are not available.

Why has the patient been invited?

The patient has been invited to take part in this trial because they are aged 16 years or older, they have been admitted to hospital, and their doctor believes they have either a suspected or confirmed COVID -19 infection and the they have higher levels of C-reactive protein in their blood than normal (this increases when there's an infection or inflammation in your body).

What will happen to the patient if they take part?

- You will agree for the patient to take part in a clinical trial and they will be actively monitored for 28 days and longer if necessary.

For peer review only - http://bmjopen.bmj.com/site/about/guidelines.xhtml

- This clinical trial has three treatment groups or 'arms': one for each of the treatments listed above, and the other arm is 'usual care'. Usual care means that the patient will receive exactly the same treatment that everyone with a (suspected or confirmed) COVID-19 infection would receive in the hospital whether you decide the patient takes part in the trial or not. The treatment the patient will receive is randomly allocated (chosen by chance) by a computer. Neither you nor the patient's doctor will be able to choose the treatment they receive. The research doctor or nurse will inform you of which treatment the patient will receive once they have entered the patient into the trial.
- If the patient is allocated the 'usual care' arm, they will receive the same medical care as all other patients being treated for a (suspected or confirmed) COVID-19 infection.
- If the patient is allocated to receive namilumab, they will receive this in addition to the usual medical care received by patients who have this disease. Namilumab will be given to the patient through a drip in a vein, usually in their arm, on one occasion only.
- If the patient is allocated to receive infliximab, they will receive this in addition to the usual medical care received by patients who have this disease. Infliximab will be given to the patient through a drip in a vein, usually in their arm, on one occasion only.
- The patient will have routine blood samples whilst they are in the hospital as part of 'usual care.' If the patient is allocated to the namilumab or infliximab arm, the patient may have more blood samples taken regularly before and during treatment to check it is safe for them to receive treatment.
- If the patient is a female of childbearing potential, a pregnancy test will be performed as part of the screening tests.
- We would also like your permission to collect samples for research from the patient. The collection of these samples is optional. **This will only take place at certain hospitals.** The doctor will tell you if research samples are being collected at the patient's hospital. This includes collecting samples of blood, and may include swabs from their nose and throat, and if they are placed on a ventilator, samples of the secretions from the tube placed in their windpipe to help them breathe may be taken. Samples will be collected on up to three separate occasions. These samples will be used in laboratory studies to gain a better understanding of COVID-19 and how patients respond to treatment.
- While the patient is in hospital their health and wellbeing will be monitored in accordance with usual care

What are the potential benefits of taking part?

This research has been designed to help develop a treatment for future patients with (suspected or confirmed) COVID-19 infection. It is important to understand that the patient may not directly benefit from taking part in this trial. The benefits for the patient as an individual are unknown.

For peer review only - http://bmjopen.bmj.com/site/about/guidelines.xhtml

What are the possible disadvantages and risks of taking part?

This trial is testing a new way of treating (suspected or confirmed) COVID-19 infection and the patient may have side effects from the treatment while taking part in the trial.

All of the treatments have been given to humans before but not in (suspected or confirmed) COVID-19 infection so there may be side effects that the doctors are not currently aware of, and these may be serious; because of this an independent committee will be monitoring the safety of the treatments in this trial on a regular basis.

Everyone taking part in the trial will be monitored carefully for side effects. However, the doctors do not know all the side effects that may occur and we don't know how the drugs used in this trial will interact with the other drugs being used to treat (suspected or confirmed) COVID-19. Side effects may be mild or serious or may even be life-threatening. The doctors may give the patient medicines to help lessen side effects or the trial treatment may be postponed or stopped, depending on the side-effects they may experience.

Specific information is provided below for each of the trial treatments available in the trial is included below:

Namilumab

Namilumab is a new drug that is being tested to treat diseases that cause inflammation such as rheumatoid arthritis.

The side effects that are known so far are:

- Low neutrophil count (a type of white blood cell)
- Minor symptoms such as runny nose and headache
- Tachycardia (a fast heart beat)*

* A short change in heart rhythm was seen with no apparent harm in one person receiving namilumab in another trial.

Infliximab

Infliximab has been widely used for rheumatoid arthritis and other conditions for 20 years but it has some potential side effects. It has been used to treat patients with sepsis (serious infection) before on intensive care units and was demonstrated to be safe. The more common known side effects of infliximab when used to treat other conditions such as arthritis have been summarised below:

Whilst receiving Infliximab:

- Common side effects (experienced by 1 in 100 people) include:

For peer review only - http://bmjopen.bmj.com/site/about/guidelines.xhtml

- Allergic reaction: this can be mild but is sometimes severe and may even be life-threatening. For this reason the patient will be very carefully monitored while receiving the iInfliximab infusion and for the period afterwards
- Feeling and being sick
- Headache
- Flu-like symptoms

During the days or weeks receiving Infliximab:

Very common side effects (experienced by 1 in 10 people) include:

- Increased risk of infection. Infliximab can interfere with the body's ability to fight other infections caused by bacteria, fungi, other viruses such as hepatitis B, and tuberculosis. The patient will be monitored carefully for signs of infection other than COVID-19 and be offered appropriate treatment if another infection is detected.

Common side effects (experienced by between 1 in 10 to 1 in 100 people) include:

- Abnormal liver function tests. Blood tests will be done frequently to check that the patient's liver is working properly.
- Some patients may experience worsening of psoriasis

Pregnancy and Breast Feeding

There is very little known about the effects of namilumab on an unborn baby and there is some information available about the and infliximab on an unborn baby. As a precaution, women who are breast feeding are excluded from the trial and women of child bearing potential must have a negative pregnancy test prior to startng the trial. It is important that if the patient receive's a trial treatment that they use adequate birth control if they (or their partner if they are male) are of child bearing potential. For namilumab, male and female participants should use effective contraception for 18 weeks after the last dose of drug. For infliximab, male and female participants should use contraception for 26 weeks after the last dose of drug.

Effective contraception is a method that has a failure rate of less than 1% a year when used correctly and all the time. Examples of these include:

- combined (oestrogen and progesterone containing) hormonal contraception e.g. the "pill", patch
- progesterone only contraception (includes the "mini-pill", injection, implant)
- Intrauterine device (IUD) or hormone-releasing system (IUS)
- vasectomised partner
- sexual abstinence

For peer review only - http://bmjopen.bmj.com/site/about/guidelines.xhtml

Recent guidelines in rheumatology patients suggests infliximab can be given to patients up until the 16th week of pregnancy. However, as this is the first time infliximab is being used in (suspected or confirmed) COVID-19 patients it is important that the patient understands the contracpetion information above.
It is also important that any female patients do not breastfeed for 6 months after the last dose of namilumab or infliximab.

Is there any prohibited medication whilst the patient is on the trial?

The trial doctor will look at the medicines that the patient is already taking and let you know whether the patient is able to still take them while they are taking part in the trial, or whether they would need to stop any of them.

What will happen if I don't want the patient to carry on with the trial?

You can withdraw the patient from the trial at any time without this having any effect on their medical care, however all information and blood samples already collected from the patient's time on the trial will still be used.

Will their taking part in this trial be kept confidential?

All information collected about the patient for this trial will be subject to the General Data Protection Regulation 2018 and Data Protection Act 2018 for Health and Care Research and will be kept strictly confidential.

The University of Birmingham is the Sponsor for this trial. The University of Birmingham will be using information from the patient's medical records in order to undertake this trial and will act as the data controller. This means that the University of Birmingham are responsible for looking after the patient's information and using it properly. The University of Birmingham and the NHS will keep identifiable information about the patient for at least 10 years after the trial has finished; this allows the results of the trial to be verified if needed.

In the Trial Office the patient will be identified by a unique trial number. In routine communication between their hospital and the Trial Office the patient will only be identified by trial number, initials and date of birth. Information about the patient, their health and wellbeing may be provided to the Trial Office on paper or electronically. We would also like to collect the patient's NHS Number. This will allow researchers to collect information about their health and wellbeing from national records (e.g. Office for National Statistics, NHS Central Registries or other registries including those managed by NHS Digital) after the trial has ended. This will help us to determine the long-term impact of the trial treatment and COVID-19 on people's health.

For peer review only - http://bmjopen.bmj.com/site/about/guidelines.xhtml

By taking part in the trial, you will be agreeing to allow research staff from the Trial Office to look at the trial records, including the patient's medical records. It may be necessary to allow authorised personnel from government regulatory agencies (e.g. Medicines and Healthcare products Regulatory Agency (MHRA)), the Sponsor and/or NHS bodies to have access to information about the patient. This is to ensure that the trial is being conducted to the highest possible standards.

If the patient is randomised to receive namilumab or infliximab, pseudo-anonymised data from the trial may also be provided to the pharmaceutical company who is providing the drug the patient is given for safety monitoring or licensing purposes, where applicable. This is for the patient's and others' protection to track the safety of the trial treatment. This may involve sending data outside of the United Kingdom to a European country of the United States of America. The patient's name and any identifying details (such as date of birth) will not be given to any of these parties.

We may be asked to share the trial information (data) we have collected with researchers running other studies, so that they can perform analysis on the data to answer other important questions about COVID-19. These other researchers may be based in universities, NHS organisations, companies involved in health research and may be in this country or abroad. Non-identifiable summary information may also be shared with COVID-19 related UK government departments. Any such request is carefully considered by the trial researchers and will only be granted if the research is of high scientific standard and the necessary procedures and approvals are in place. The information will only be used for the purpose of health research and cannot be used to contact you, the patient, or to affect the patient's care. It will not be used to make decisions about future services available to them, such as insurance.

All individuals who have access to the patient's information have a duty of confidentiality to them. Under no circumstances will the patient be identified in any way in any report, presentation or publication arising from this or any other research study.

You can withdraw your consent to our processing any more of the patient's data at any time. Your rights to access change or move the patient's information are limited, as we need to manage the information in specific ways in order for the research to be reliable and accurate. If you withdraw the patient from the trial, we will keep the information about them and the samples that we have already obtained. Under the provisions of the General Data Protection Regulation 2018 and Data Protection Act 2018 the patient has the right to know what information the University of Birmingham has recorded about the patient. If the patient wishes to view this information or find more about how we use this information, they should contact Legal Services at Legal Services, University of Birmingham, Edgbaston, Birmingham, B15 2TT.

For peer review only - http://bmjopen.bmj.com/site/about/guidelines.xhtml

What will happen to the samples the patient gives?

If you agree for the patient to take part in the optional research sample collection, the samples will be sent to laboratories based in the Universities of Birmingham or Oxford for storage and use in laboratory-based research for the trial. If you give your consent, any samples leftover at the end of analysis for the trial, may be kept for future ethically approved research. These laboratory-based research projects will include genetic analysis of these samples. This DNA and RNA analysis is for scientific purposes and is not expected to provide findings of any clinical significance for the patient or their relatives, so the results will not be fed back to the patient. It is difficult to predict precisely what scientific developments there may be so we cannot give precise details of what research might be done.

No one using the samples for laboratory research will have access to the patient's personal details. The samples sent to the University laboratories will be identified by the patient's unique trial number only. All samples relating to the patient will be stored in accordance with the Human Tissue Act 2004.

What will happen to the results of the research trial?

At the end of the trial, the findings will be published in peer-reviewed medical and scientific journals. These publications will be available to the patient upon request from their trial doctor. We will also make a lay summary of the result available on the trial websites.

Who is organising and funding the research?

The trial is sponsored and being undertaken by the University of Birmingham in collaboration with University Hospitals Birmingham NHS Foundation Trust The trial is being coordinated by the Cancer Research UK Clinical Trials Unit (CRCTU) within the University of Birmingham.

The trial is funded by an educational grant from UK Research and Innovation and drugs are being provided free of charge by the pharmaceutical companies.

Who has reviewed the trial?

All research in the NHS is looked at by an independent group of people called a Research Ethics Committee to protect the patient's safety, rights, wellbeing and dignity. This trial has been reviewed and given favourable opinion by the East Midlands-Nottingham 2 Research Ethics Committee and by the NHS Health Research Authority. While the trial is ongoing the results will be reviewed regularly by an independent Data Monitoring Committee to ensure that it is appropriate to continue with the trial.

For peer review only - http://bmjopen.bmj.com/site/about/guidelines.xhtml

Expenses and Payments

As the patient will already be an inpatient in the hospital during the course of the trial they will not have to make any extra visits to participate in the trial and therefore will incur no additional expenses.

What if there is a problem?

If you have a concern about any aspect of this trial, you should ask to speak with the patient's trial doctor who will do their best to answer your questions (see contact number at the end of this form). If you remain unhappy and wish to complain formally, you can do this through the hospital's Patient Advice and Liaison Services (PALS); they can be contacted by:

(Insert local contact details).

If the patient is harmed and this is due to someone's negligence then there may be grounds for legal action for compensation against the Sponsor of the trial (University of Birmingham) or the NHS Trust. NHS Trust Hospitals have a duty of care to all patients treated, whether or not the patient is taking part in a clinical trial, and the normal NHS complaints mechanisms will still be available to them (if appropriate).

Further information and contact details

Trial Doctor: ____

Research Nurse: ____

☎: ____________________ Emergency (24 hours) ☎: ____________________

For peer review only - http://bmjopen.bmj.com/site/about/guidelines.xhtml

Supplementary Appendix 3 - Informed Consent Forms

<To be printed on hospital headed paper>

CATALYST- Early phase platform trial in (suspected or confirmed) COVID-19

Which treatment could lessen the severity of a (suspected or confirmed) COVID-19 infection when compared with usual care in an NHS setting?

For peer review only

Informed Consent Form

Site: ....

Patient's Trial Number: [ ][ ][ ]

Investigator: ....

EudraCT: 2020-001684-89

Please initial each box

1. I confirm that I have read and understood the Patient Information Sheet (version .... dated..........................) for the above trial. I have had the opportunity to consider the information, ask questions and have had these answered satisfactorily. [ ]

2. I understand that my participation is voluntary and that I am free to withdraw at any time without giving any reason, without my medical care or legal rights being affected. [ ]

3. I give permission for my initials, date of birth and NHS Number to be given to the Trial Office based within the University of Birmingham (Birmingham, UK) when I am randomised into the trial. [ ]

Original to be kept in the Investigator Site File, 1 copy in hospital notes, 1 copy to the patient

For peer review only - http://bmjopen.bmj.com/site/about/guidelines.xhtml

4. I understand that relevant sections of my medical notes and data collected during the trial may be looked at by individuals from the Trial Office, regulatory authorities, the Sponsor (the University of Birmingham) and/or NHS bodies, where it is relevant to my taking part in this research. I give permission for these individuals to have access to my records.

5. I understand that data and samples from the trial may be provided to third parties (e.g. laboratories, pharmaceutical companies, other academic institutions or relevant UK government departments) for research, safety monitoring or licensing purposes, where applicable. I understand that this may involve sending data outside of the United Kingdom to a European country or the United States of America and that my name and any identifying details will NOT be given to these third parties, instead I will be identified by my unique Trial Number.

6. I understand that if I withdraw from the trial, any data collected up until the date of my withdrawal will be analysed and used as part of the research.

7. I agree to my GP being informed of my participation in this trial.

8. I agree to take part in the above trial.

OPTIONAL

9. I agree to the collection and storage of research samples for analysis as part of the CATALYST trial and for use in other ethically approved laboratory projects, which may include genetic analysis of these samples. I understand that I will not be personally informed of the results of these additional research projects and that if I withdraw from the trial any samples that have been collected up to the date of my withdrawal will not be destroyed.

For peer review only - http://bmjopen.bmj.com/site/about/guidelines.xhtml

______________________	______________	__________________
**Name of patient**	**Date**	**Signature**
________________________	______________	____________________
**Name of person taking consent**	**Date**	**Signature**

You must have signed the Site Signature & Delegation Log

Original to be kept in the Investigator Site File, 1 copy in hospital notes, 1 copy to the patient

For peer review only - http://bmjopen.bmj.com/site/about/guidelines.xhtml

<To be printed on hospital headed paper>

CATALYST- Early phase platform trial in (suspected or confirmed) COVID 19

Which treatment could lessen the severity of a (suspected or confirmed) COVID 19 infection when compared with usual care in an NHS setting?

For peer review only

Personal Representative Informed Consent Form

Site: ………………………………………… **Patient's Trial Number:** [ ][ ][ ]

Investigator: ………………………………………… **EudraCT:** 2020-001684-89

Please initial each box

1. I confirm that I been consulted about ………………………………….'s participant in this trial and have read and understand the Personal Legal Representative Information Sheet (version .... dated.......................) for the above trial and what it means to be a personal legal representative. I have had the opportunity to consider the information, ask questions and have had these answered satisfactorily. [ ]

2. I understand that his/her participation is voluntary and that I am free to change my opinion on what he/she would have wished for at any time without giving any reason, and without his/her medical care or legal rights being affected. [ ]

3. I understand that his/her direct informed consent will be obtained at the earliest opportunity. [ ]

Original to be kept in the Investigator Site File, 1 copy in hospital notes, 1 copy to the patient/ legal representative

For peer review only - http://bmjopen.bmj.com/site/about/guidelines.xhtml

4. I understand that his/her initials, date of birth and NHS Number will be given to the Trial Office based within the University of Birmingham (Birmingham, UK) when he/she is randomised to the trial.

5. I understand that relevant sections of his/her medical notes and data collected during the trial may be looked at by individuals from the Trial Office, regulatory authorities, the Sponsor (the University of Birmingham) and/or NHS bodies, where it is relevant to him/her taking part in this research.

6. I understand that data and samples from the trial may be provided to third parties (e.g. laboratories, pharmaceutical companies, other academic institutions or relevant UK government departments) for research, safety monitoring or licensing purposes, where applicable. I understand that this may involve sending data outside of the United Kingdom to a European country or the United States of America and that the patients name and any identifying details will NOT be given to these 3rd parties, instead he/she will be identified by his/ her unique Trial Number.

7. I understand that if he/she withdraws or is withdrawn from the trial, any samples and data that have been collected up to the date of withdrawal will be analysed and used as part of the research.

8. I agree to the patients GP being informed of his/ her participation in this trial.

9. In my opinion, the patient would have no objections to taking part in the above trial

OPTIONAL

10. I agree to the collection and storage of research samples for analysis as part of the CATALYST trial and for use in other ethically approved laboratory projects, which may include genetic analysis of these

For peer review only

For peer review only - http://bmjopen.bmj.com/site/about/guidelines.xhtml

samples. I understand that the patient will not be personally informed of the results of these additional research projects and that if he/ she withdraws from the trial any samples that have been collected up to the date of his/her withdrawal will not be destroyed.

Name of patient: ____

______________________________ ________________ ________________________

Name of Personal Representative **Date** **Signature**

____

Relationship to patient

______________________________ ________________ ________________________

Name of person taking consent **Date** **Signature**

You must have signed the Site Signature & Delegation Log

Original to be kept in the Investigator Site File, 1 copy in hospital notes, 1 copy to the patient/ legal representative

For peer review only - http://bmjopen.bmj.com/site/about/guidelines.xhtml

<To be printed on hospital headed paper>

CATALYST- Early phase platform trial in (suspected or confirmed) COVID 19

Which treatment could lessen the severity of a (suspected or confirmed) COVID 19 infection when compared with usual care in an NHS setting?

Professional Legal Representative Informed Consent Form

Site: .... **Patient's Trial Number:** [ ][ ][ ]

Investigator: .... **EudraCT:** 2020-001684-89

Please initial each box

1. I confirm that I been consulted about ....'s participant in this trial and have read and understand the Professional Legal Representative Information Sheet (version .... dated.......................) for the above trial and what it means to be a professional legal representative. I have had the opportunity to consider the information, ask questions and have had these answered satisfactorily. [ ]

2. I understand that his/her participation is voluntary and that I am free to change my opinion on what he/she would have wished for at any time without giving any reason, and without his/her medical care or legal rights being affected. [ ]

3. I understand that his/her direct informed consent will be obtained at the earliest opportunity. [ ]

Original to be kept in the Investigator Site File, 1 copy in hospital notes, 1 copy to the patient/ legal representative,

For peer review only - http://bmjopen.bmj.com/site/about/guidelines.xhtml

4. I understand that his/her initials, date of birth and NHS Number will be given to the Trial Office based within the University of Birmingham (Birmingham, UK) when he/she is randomised to the trial.

5. I understand that relevant sections of his/her medical notes and data collected during the trial may be looked at by individuals from the Trial Office, regulatory authorities, the Sponsor (the University of Birmingham) and/or NHS bodies, where it is relevant to him/her taking part in this research.

6. I understand that anonymised data and samples from the trial may be provided to third parties (e.g. laboratories, pharmaceutical companies, other academic institutions or relevant UK government departments) for research, safety monitoring or licensing purposes, where applicable. I understand that this may involve sending data outside of the United Kingdom to a European country or the United States of America and that the patients name and any identifying details will NOT be given to these third parties, instead he/ she will be identified by his/ her unique Trial Number.

7. I understand that if he/she withdraws or is withdrawn from the trial, any samples and data that have been collected up to the date of withdrawal will be analysed and used as part of the research.

8. I agree to the patients GP being informed of his/ her participation in this trial.

9. In my opinion, the patient would have no objections to taking part in the above trial

OPTIONAL

10. I agree to the collection and storage of research samples for analysis as part of the CATALYST trial and for use in other ethically approved laboratory projects, which may include genetic analysis of these

For peer review only - http://bmjopen.bmj.com/site/about/guidelines.xhtml

For peer review only

samples. I understand that the patient will not be personally informed of the results of these additional research projects and that if he/ she withdraws from the trial any samples that have been collected up to the date of his/her withdrawal will not be destroyed.

____

Name of patient:

________________________________	____________________	____________________________
**Name of Professional Legal Representative**	**Date**	**Signature**
___________________________________	____________________	____________________________
**Name of person taking consent**	**Date**	**Signature**

You must have signed the Site Signature & Delegation Log

For peer review only

Original to be kept in the Investigator Site File, 1 copy in hospital notes, 1 copy to the patient/ legal representative,

For peer review only - http://bmjopen.bmj.com/site/about/guidelines.xhtml

Supplementary Appendix 4 – Statistical considerations

The simulations and tables below demonstrate the operating characteristics of a trial design with the chosen decision criteria, based on a simpler analysis of the area under the curve for sequential CRP data, with effect sizes informed from a dataset from 1026 hospitalised COVID-19 patients at Queen Elizabeth Hospital, Birmingham. In our simulations, we compared a traditional fixed trial design recruiting 120 patients with candidate adaptive designs. We present basic operating characteristics for the fixed design (Table 2A) and the chosen adaptive design (Table 2B). We studied six scenarios of treatment effect (see Table 5), and estimated, through simulation, the probability of a trial stopping early for "success" or "futility," and ultimately concluding success.

Table 2A. Operating characteristics for a fixed trial design of 120 patients.

Scenario	Probability stopping early for success	Probability stopping early for futility	Overall probability of success	Mean number of patients
Null	0	0	0.101	120
A	0	0	0.537	120
B	0	0	0.926	120
C	0	0	0.997	120
D	0	0	0.008	120
E	0	0	0	120

Scenarios A, B, and C are beneficial effects of the intervention with (true) treatment effects of 0.25, 0.5 and 0.75 standard deviations, "null" is zero treatment effect and D and E are harmful effects of 0.25 and 0.5 standard deviations. "success" and "futility" are defined as above.

Table 2B. Operating characteristics for an adaptive design with interim analyses at 40 and 80 patients.

Scenario	Probability stopping early for success	Probability stopping early for futility	Overall probability of success	Mean number of patients
Null	0.148	0.624	0.176	66
A	0.455	0.281	0.559	70
B	0.798	0.089	0.890	59
C	0.965	0.012	0.985	48
D	0.03	0.901	0.031	52
E	0.003	0.986	0.003	43

The adaptive design achieves similar probabilities of success in scenarios where the treatment effect is truly beneficial (A, B and C), and increases the probability of success only slightly if the intervention is harmful (D and E). There is some increase in the probability of success if the treatment effect is zero (Type I error) but this is offset by the very substantial reductions in the numbers of patients needed in all scenarios. Moreover, Type I error is not a serious problem as all interventions would be evaluated further in phase III trials.

For peer review only - http://bmjopen.bmj.com/site/about/guidelines.xhtml

Supplementary Appendix 5 – Adverse event definitions

Adverse Event

Any untoward medical occurrence in a patient or clinical trial subject administered a medicinal product and which does not necessarily have a causal relationship with this treatment.

Comment:

An AE can therefore be any unfavourable and unintended sign (including abnormal laboratory findings), symptom or disease temporally associated with the use of an investigational medicinal product, whether or not related to the investigational medicinal product.

Adverse Reaction

All untoward and unintended responses to an IMP related to any dose administered.

Comment:

An AE judged by either the reporting Investigator or Sponsor as having causal relationship to the IMP qualifies as an AR. The expression reasonable causal relationship means to convey in general that there is evidence or argument to suggest a causal relationship.

Serious Adverse Event

Any untoward medical occurrence or effect that at any dose:

Results in death

Is life threatening*

Requires hospitalisation** or prolongation of existing inpatients' hospitalisation

Results in persistent or significant disability or incapacity

Is a congenital anomaly/birth defect

Or is otherwise considered medically significant by the investigator ***

Comments:

The term severe is often used to describe the intensity (severity) of a specific event. This is not the same as serious, which is based on patients/event outcome or action criteria.

* Life threatening in the definition of an SAE refers to an event in which the patient was at risk of death at the time of the event; it does not refer to an event that hypothetically might have caused death if it were more severe.

** hospitalisation is defined as an unplanned, formal inpatient admission, even if the hospitalisation is a precautionary measure for continued observation. Thus, hospitalisation for protocol treatment (e.g. line insertion), elective procedures (unless brought forward because of worsening symptoms) or for social reasons (e.g. respite care) are not regarded as an SAE.

*** Medical judgment should be exercised in deciding whether an AE is serious in other situations. Important AEs that are not immediately life threatening or do not result in death or hospitalisation but may jeopardise the subject or may require intervention to prevent one of the other outcomes listed in the definition above, should be considered serious.

Serious Adverse Reaction

An Adverse Reaction which also meets the definition of a Serious Adverse Event.

Suspected Unexpected Serious Adverse Reaction

A SAR that is unexpected i.e. the nature, or severity of the event is not consistent with the applicable product information.

For peer review only - http://bmjopen.bmj.com/site/about/guidelines.xhtml

A SUSAR should meet the definition of an AR, UAR and SAR.

Unexpected Adverse Reaction

An AR, the nature or severity of which is not consistent with the applicable product information (e.g. Investigator Brochure for an unapproved IMP or (compendium of) Summary of Product Characteristics (SPC) for a licensed product).

When the outcome of an AR is not consistent with the applicable product information the AR should be considered unexpected.

For peer review only

For peer review only - http://bmjopen.bmj.com/site/about/guidelines.xhtml

BMJ Open

CATALYST trial protocol: A multicentre, open-label, phase II, multi-arm trial for an early and accelerated evaluation of the potential treatments for COVID-19 in hospitalised adults

Journal:	*BMJ Open*
Manuscript ID	bmjopen-2021-050202.R1
Article Type:	Protocol
Date Submitted by the Author:	06-Sep-2021
Complete List of Authors:	Veenith, Tonny; University of Birmingham, Birmingham Acute Care Research Group, Institute of Inflammation and Ageing, College of Medical and Dental Sciences; University Hospitals Birmingham NHS Foundation Trust, Department of Critical Care Medicine<br>Fisher, Benjamin; University of Birmingham College of Medical and Dental Sciences, Institute of Inflammation and Ageing; University of Birmingham Cancer Research UK Clinical Trials Unit<br>Slade, Daniel; University of Birmingham, Cancer Research UK Clinical Trials Unit, Institute of Cancer and Genomic Sciences<br>Rowe, Anna; University Hospitals Birmingham NHS Foundation Trust, National Institute for Health Research (NIHR) Birmingham Biomedical Research Centre; University of Birmingham, Cancer Research UK Clinical Trials Unit, Institute of Cancer and Genomic Sciences<br>Sharpe, Rowena; University of Birmingham, Cancer Research UK Clinical Trials Unit, Institute of Cancer and Genomic Sciences<br>Thickett , David ; University of Birmingham, Birmingham Acute Care Research Group, Institute of Inflammation and Ageing, College of Medical and Dental Sciences, ; University Hospitals Birmingham NHS Foundation Trust, Department of Respiratory Medicine<br>Whitehouse, Tony; University Hospitals Birmingham NHS Foundation Trust, Department of Critical Care Medicine; University of Birmingham, Birmingham Acute Care Research Group, Institute of Inflammation and Ageing, College of Medical and Dental Sciences,<br>Rowland, Matthew; University of Oxford, Nuffield Department of Clinical Neurosciences, John Radcliffe Hospital,<br>Scriven, James; University Hospitals Birmingham NHS Foundation Trust, Department of Infectious Diseases<br>Parekh, Dhruv; University of Birmingham, Birmingham Acute Care Research Group, Institute of Inflammation and Ageing, College of Medical and Dental Sciences, ; University Hospitals Birmingham NHS Foundation Trust, Department of Critical Care Medicine, and Department of Respiratory Medicine<br>Bowden, Sarah; University of Birmingham, Cancer Research UK Clinical Trials Unit, Institute of Cancer and Genomic Sciences,<br>Savage, Joshua; University of Birmingham, Cancer Research UK Clinical Trials Unit, Institute of Cancer and Genomic Sciences,<br>Richards, Duncan; University of Oxford, Oxford Clinical Trials Research Unit, Botnar Research Centre,<br>Bion, Julian; University Hospitals Birmingham NHS Foundation Trust,

	Department of Critical Care Medicine,<br>Kearns, Pamela; University Hospitals Birmingham NHS Foundation Trust, National Institute for Health Research (NIHR) Birmingham Biomedical Research Centre, ; University of Birmingham, Cancer Research UK Clinical Trials Unit, Institute of Cancer and Genomic Sciences,<br>Gates, Simon; University of Birmingham, Cancer Research UK Clinical Trials Unit, Institute of Cancer and Genomic Sciences,
**Primary Subject Heading**:	Intensive care
Secondary Subject Heading:	Infectious diseases
Keywords:	Adult intensive & critical care < ANAESTHETICS, COVID-19, INFECTIOUS DISEASES, Thoracic medicine < INTERNAL MEDICINE, Rheumatology < INTERNAL MEDICINE

SCHOLARONE™
Manuscripts

I, the Submitting Author has the right to grant and does grant on behalf of all authors of the Work (as defined in the below author licence), an exclusive licence and/or a non-exclusive licence for contributions from authors who are: i) UK Crown employees; ii) where BMJ has agreed a CC-BY licence shall apply, and/or iii) in accordance with the terms applicable for US Federal Government officers or employees acting as part of their official duties; on a worldwide, perpetual, irrevocable, royalty-free basis to BMJ Publishing Group Ltd ("BMJ") its licensees and where the relevant Journal is co-owned by BMJ to the co-owners of the Journal, to publish the Work in this journal and any other BMJ products and to exploit all rights, as set out in our licence.

The Submitting Author accepts and understands that any supply made under these terms is made by BMJ to the Submitting Author unless you are acting as an employee on behalf of your employer or a postgraduate student of an affiliated institution which is paying any applicable article publishing charge ("APC") for Open Access articles. Where the Submitting Author wishes to make the Work available on an Open Access basis (and intends to pay the relevant APC), the terms of reuse of such Open Access shall be governed by a Creative Commons licence – details of these licences and which Creative Commons licence will apply to this Work are set out in our licence referred to above.

Other than as permitted in any relevant BMJ Author's Self Archiving Policies, I confirm this Work has not been accepted for publication elsewhere, is not being considered for publication elsewhere and does not duplicate material already published. I confirm all authors consent to publication of this Work and authorise the granting of this licence.

For peer review only

CATALYST trial protocol: A multicentre, open-label, phase II, multi-arm trial for an early and accelerated evaluation of the potential treatments for COVID-19 in hospitalised adults

Tonny Veenith[1, 2]*, Benjamin A. Fisher[3,4,5]*, Daniel Slade[5], Anna Rowe[4,5], Rowena Sharpe[5], David R. Thickett[2,6], Tony Whitehouse[1,2], Matthew Rowland[7], James Scriven[8], Dhruv Parekh[1,2,6], Sarah J. Bowden[5], Joshua S. Savage[5], Duncan Richards[9], Julian Bion[1]†, Pamela Kearns[4,5]†, Simon Gates[5]†, on behalf of CATALYST investigators[&]

* These authors have made an equal contribution.

† These authors have made an equal contribution.

[&] Additional investigators of the CATALYST Trial are listed in Supplementary Appendix 1.

* Corresponding authors contact details:

Prof. Tonny Veenith, Institute of Inflammation and Ageing, College of Medical and Dental Sciences, University of Birmingham, Birmingham, B15 2TT, UK. Email: t.v.veenith@bham.ac.uk or;

Dr Benjamin A. Fisher, Institute of Inflammation and Ageing, College of Medical and Dental Sciences, University of Birmingham, Birmingham, B15 2TT, UK. Email: b.fisher@bham.ac.uk.

1. Department of Critical Care Medicine, University Hospitals Birmingham NHS Trust, United Kingdom
2. Birmingham Acute Care Research Group, Institute of Inflammation and Ageing, College of Medical and Dental Sciences, University of Birmingham, UK
3. Rheumatology Research Group, Institute of Inflammation and Ageing, College of Medical and Dental Sciences, University of Birmingham, UK
4. National Institute for Health Research (NIHR) Birmingham Biomedical Research Centre, University Hospitals Birmingham NHS Foundation Trust, Birmingham, UK.
5. Cancer Research UK Clinical Trials Unit, Institute of Cancer and Genomic Sciences, University of Birmingham, Birmingham, UK
6. Department of Respiratory Medicine, University Hospitals Birmingham NHS Trust, UK
7. Nuffield Department of Clinical Neurosciences, University of Oxford, John Radcliffe Hospital, Oxford, UK.
8. Department of Infectious Diseases, University Hospitals Birmingham NHS Trust, UK
9. Oxford Clinical Trials Research Unit, Botnar Research Centre, University of Oxford, Oxford, UK.

For peer review only

For peer review only - http://bmjopen.bmj.com/site/about/guidelines.xhtml

Abstract

Introduction

Severe SARS-CoV-2 infection is associated with a dysregulated immune response. Inflammatory monocytes and macrophages are crucial, promoting injurious, pro-inflammatory sequelae. Immunomodulation is, therefore, an attractive therapeutic strategy and we sought to test licensed and novel candidate drugs.

Methods and analysis

The CATALYST trial is a multi-arm, open-label, multi-centre, phase II platform trial designed to identify candidate novel treatments to improve outcomes of patients hospitalised with COVID-19 compared with usual care. Treatments with evidence of biomarker improvements will be put forward for larger-scale testing by current national phase III platform trials. Hospitalised patients $\geq$16 years with a clinical picture strongly suggestive of SARS-CoV-2 pneumonia (confirmed by chest X-ray or CT scan, with or without a positive reverse transcription polymerase chain reaction (RT-PCR) assay) and a C-Reactive Protein (CRP) ≥40 mg/L are eligible. The primary outcome measure is CRP, measured serially from admission to day 14, hospital discharge or death. Secondary outcomes include the WHO Clinical Progression Improvement Scale as a principal efficacy assessment.

Ethics and dissemination

The protocol was approved by the East Midlands - Nottingham 2 Research Ethics Committee (20/EM/0115) and given Urgent Public Health status; initial approval was received on 05-May-2020, current protocol version (v6.0) approval on 12-Oct-2020. The MHRA also approved all protocol versions. The results of this trial will be disseminated through national and international presentations and peer-reviewed publications.

Trial registration number

EudraCT Number: 2020-001684-89

ISRCTN Number: 40580903

Strengths and limitations of this trial

- CATALYST will provide a rapid readout on the safety and proof-of-concept of candidate novel treatments
- CATALYST will enable phase III trial resources to be focussed and allocated for agents with a high likelihood of success
- CATALYST uses Bayesian multi-level models to allow for nesting of repeated measures data, with factors for each individual patient and treatment arm, and allowing for non-linear responses
- CATALYST is not designed to provide a definitive signal on clinical outcomes

For peer review only - http://bmjopen.bmj.com/site/about/guidelines.xhtml

Introduction

Since the start of the pandemic, the UK has now passed the milestone of 100,000 deaths due to SARS-CoV-2 virus. Current data from the International Severe Acute Respiratory and emerging Infections Consortium (ISARIC) partnerships indicate that 20% of patients admitted to the hospital require critical care support, with an overall mortality of 34.5% after hospital admission in the last two waves (1). Mortality following respiratory support remains over 40% in the second wave (2). Recovery from severe disease may be associated with long-term health impact (3, 4). Despite the introduction of vaccination programmes in December-2020, there remains an urgent need to identify agents which prevent progression to critical illness, reduce morality and promote rapid recovery.

SARS-CoV-2 can cause severe pneumonia with diffuse alveolar damage, infiltrating perivascular lymphocytes, disrupted endothelial cell membranes, vascular thrombosis with microangiopathy and occlusion of alveolar capillaries (5). Subsequent multiple organ failure, is in part, driven by a dysregulated immune response. Inflammatory monocytes and macrophages contribute to endothelial damage and microthrombosis and drive cytokine production. The cellular response is characterised by an upregulation of pro-inflammatory cytokines and chemokines, leading to a host immune response targeted at the virus, damaging host tissues (6). The severity of the disease is proportional to the cytokine response (interleukin (IL)-6, interferon gamma inducible protein (IP)-10, monocyte chemoattractant protein (MCP)-1, macrophage inflammatory protein (MIP)-1A, and tumour necrosis factor (TNF)-α), with critically ill patients exhibiting the highest levels of cytokines and chemokines (7-9). This dysregulated immunity may be a modifiable pathobiological therapeutic target for preventing COVID-19 progression. Potential mechanisms underlying this immune pathobiology may be targeted with precision using existing licensed and novel drugs. The CATALYST platform, therefore, aims to study these drugs and other novel therapeutic options for rapid assessment of safety, biological signal for efficacy, and providing secure underpinning of science prior to large phase III trials. Those with potential efficacy can then be considered for larger-scale testing by national platform trials such as RECOVERY (10) or REMAP-CAP (11).

Methods and analysis

Study design

The CATALYST trial is a multi-arm, randomised, open-label phase II clinical trial. Initial candidate drugs included within the multi-arm design were namilumab, and infliximab, monoclonal antibodies targeting the pro-inflammatory cytokines granulocyte-macrophage colony-stimulating factor (GM-CSF) and TNF-α respectively. A third agent, gemtuzumab-ozogamicin (Mylotarg), was incorporated in the protocol but has not been prioritised for evaluation at this stage and has not opened to recruitment. Each candidate therapy will be given in addition to usual care and compared with usual care independently (Figure 1). Randomisation will be performed by an automated minimisation procedure that attempts to allocate participants in a balanced manner between treatment groups allowing for the stratification variable (ward or ICU) and with a 20% random component. The World Health Organization (WHO) Trial Registration Data Set is attached in Supplementary Appendix 2.

New therapies may be introduced sequentially utilising this design. Therapies for evaluation will be based on the scientific rationale prioritised by a Scientific Advisory Board (SAB). This will enable the rapid addition of new treatment arms, whilst allowing the efficacious assessment and analysis of each of these treatment arms (12, 13). Participants are followed up for at least 28 days post-randomisation. A list of protocol changes is detailed in Supplementary Appendix 3.

For peer review only - http://bmjopen.bmj.com/site/about/guidelines.xhtml

Patient and public involvement (PPI)

From its inception, the CATALYST trial was co-developed with the Critical Care PPI group based in the Surgical Reconstruction and Microbiology Research Centre (SRMRC) at University Hospitals Birmingham. The group reviewed and refined the protocol and participant-facing documents and provided input into the design; specifically, their feedback supported a usual care arm without the inclusion of placebo controls given the context of the ongoing pandemic. PPI representatives are members of the Trial Steering Committee (TSC) who will supervise the conduct of the trial conduct, and monitor progress, including recruitment, and will support the dissemination of the trial results.

Inclusion and exclusion criteria

Hopitalised patients aged 16 years or over, with a clinical picture strongly suggestive of SARS-CoV-2 pneumonia (confirmed by chest X-ray or CT scan, with or without a positive reverse transcription-polymerase chain reaction (RT-PCR) assay), and C-Reactive Protein (CRP) greater than or equal to 40 mg/L are eligible for this study. Exclusion criteria include patient or personal/professional legal representative refusal, planned palliative care, pregnancy or breastfeeding women, women of childbearing potential and non-vasectomised men who are unwilling to use effective contraception for the duration of the trial and throughout the drug-defined post-trial period. Patients with known HIV or chronic hepatitis B or C infection, contraindications to any of the investigational medicinal products (IMPs), receiving concurrent immunosuppression with biological agents, a history of haematopoietic stem cell transplant or solid organ transplant, known hypersensitivity to drug products or excipients, tuberculosis or other severe infections such as (non-SARS-CoV-2) sepsis, abscesses, and opportunistic infections requiring treatment, moderate or severe heart failure (NYHA class III/IV), or any other indication or medical history, that in the opinion of the patient's local investigator is unsuitable for trial participation. Patients will not be eligible if they are currently participating in another COVID-19 interventional trial; co-enrolment into RECOVERY-Respiratory Support (RS) trial is allowed.

Consent

Patients are identified as per site-established processes. This may include searching central logs of patients admitted with COVID-19, or via pre-screening processes already in situ at the site. Each eligible patient who has capacity will be given a Patient Information Sheet (PIS) to read more about the trial (Supplementary Appendix 4). Informed consent is requested from patients with capacity by an investigator who has been delegated the responsibility on the delegation log. Where a patient lacks capacity (for example from severity of illness) informed consent will be sought from the patient's personal legal representative (PerLR). In the event that the PerLR is unavailable, informed consent will be sought from the patient's professional legal representative (ProfLR) according to the requirements of the UK Health Research Authority (14). Specific PISs for both PerLR and ProfLR can also be emailed to aid this process. If a patient recovers their capacity, they should be reconsented as soon as possible using the standard PIS and Informed Consent Form (ICF). Patient and PerLR forms are also available in Bengali, English, French, Polish, Portuguese, Punjabi, Urdu, and Welsh. Supplementary Appendices 4 and 5 contain the English version of the patient, ProfLR and PerLR information sheets, and ICFs, respectively.

As soon as the patient is considered eligible the site investigator or delegated team member should enter the patient into the trial by completing the Randomisation Form on the electronic Remote Data Capture (eRDC) system. This will allocate the participant, as described above, into an open trial arm.

For peer review only - http://bmjopen.bmj.com/site/about/guidelines.xhtml

Interventions

Arm 1: Usual care provided following the current institutional policy for patients with COVID-19. This may vary by site and over time. Following the recommendation from the UK CMO in June 2020, standard care includes dexamethasone treatment.

Arm 3: Usual care combined with namilumab, administered in a single dose (150mg) on day one infused intravenously over 1-hour. Namilumab is an anti-GM-CSF monoclonal antibody with a good safety profile up to phase II studies in rheumatoid arthritis (RA) and axial spondyloarthropathy with over 360 individuals dosed in total (15-18). The objective of namilumab therapy in COVID-19 is to inhibit inflammatory monocyte/macrophage activation and their trafficking to the lungs so as to reduce the aberrant immunopathology.

Arm 4: Usual care combined with infliximab, administered in a single dose of 5mg/Kg diluted in 250ml of 0.9% saline on day one and infused over a 2-hour period. Infliximab is a widely available anti-TNF alpha monoclonal antibody licensed for the treatment of a number of diseases including RA. TNF is a key pro-inflammatory cytokine produced by macrophages implicated in a number of processes contributing to early lung pathology (19).

No dose modification for namilumab or infliximab is permitted. Although there are no requirements for pre-medication, patients may receive pre-medication or treatment with antihistamines and paracetamol at local discretion to prevent or treat mild-to-moderate infusion reactions due to namilumab or infliximab administration.

Initially, gemtuzumab-ozogamicin, an antibody-drug conjugate approved for induction therapy of acute myeloid leukaemia was included for investigation in the trial (Arm 2), however, prioritisation discussions in the Government Committee overseeing the COVID-19 phase II studies, advised that this arm should be suspended without recruitment, in favour of continuing with namilumab and infliximab arms.

Concomitant medication

Upon admission to hospital, and in accordance with usual care, concomitant medication should be reviewed for contraindications to the IMPs. Where not contra-indicated, concomitant medication may be given as medically indicated and in line with the summary of product characteristics or Investigational Brochure as applicable for that IMP. There are no known contraindications for namilumab. For patients randomised to Arm 4, usual care and infliximab, the use of anakinra, abatacept, and tocilizumab is not recommended.

Trial outcomes

The primary outcome is CRP concentration over time, where a sequential reduction in one of the interventional arms as compared with usual care is considered indicative that this may be a clinically effective treatment suitable for testing in phase III clinical trials.

The secondary outcomes are aligned with Core Outcome Measures in Effectiveness Trials' initiatives (20, 21). The principal clinical efficacy measure is the WHO Clinical Progression Improvement Scale measured daily for 28 days on a 1-10 scale; level 0 (no viral load detected) will not be assessed over the course of this study (Table 1). Other clinical measures assessed until day 14, discharge, or death, include the ratio of the oxygen saturation to fractional inspired oxygen concentration (SpO_2/FiO_2), respiratory rate, body temperature, and the National Early Warning Scale 2 (NEWS-2). Assessments assessed until day 28 include length of hospital stay, hospital survival status at day 28, the proportion of patients discharged at day 28, and the destination of discharge. Routine laboratory measurements at baseline, days 3, 5, 7, 9, and 14

For peer review only - http://bmjopen.bmj.com/site/about/guidelines.xhtml

include lymphocyte count, neutrophil count, neutrophil: lymphocyte ratio, ferritin, D-Dimer and LDH. Safety measures as defined by adverse events (AEs) and as recorded by Common Terminology Criteria for Adverse Events (CTCAE), version 4.03 (22) are those of Grade ≥3, secondary infection and allergic reaction, and survival status. The schedule of events is shown in Table 2 with additional IMP-specific schedules in Supplementary Appendix 6.

If the patient is discharged from the hospital before their next scheduled visit, the participant should be provided with the WHO clinical improvement scale diary and the visits on days 7, 14 and 28 should take place by telephone (if it is not possible to see the patient). If the visit is via telephone this will include an adverse event review collect data on WHO clinical improvement scale assessment.

In a subset of the patients (those admitted to University Hospitals Birmingham NHS Trust and University of Oxford NHS Trust) optional samples consisting of whole blood (for RNA, DNA and also cellular assessments, preserved in Cytodelics buffer), peripheral blood mononuclear cells (PBMCs), and plasma, will be obtained on days one, three and nine (or day of discharge if earlier) and will broadly follow the ISARIC protocol (23). All samples will be collected in accordance with national regulations and requirements, including standard operating procedures for logistics and infrastructure. Samples will be taken in appropriately licensed premises, stored and transported per the Human Tissue Authority guidelines and NHS trust policies.

Statistical analysis plan

The primary outcome data will consist of a sequence through time of readings of each patient's CRP. These will be modelled using Bayesian multi-level models (also known as hierarchical or mixed-effects) that allow for nesting of the repeated measures data within patient, and allowing for non-linear responses. Specifically, posterior probabilities for the treatment/time interaction covariates will be used to conduct decision making. Data will be analysed for each intervention arm against the control group, including in each analysis, only participants who were eligible for that comparison. The model will be adjusted for age and care status at recruitment (ward or ICU).

At the specified decision points, with interim analysis at n=20 and n=40 and a final analysis at n=60 per arm, CRP data will be considered in the context of the emerging safety data to make a recommendation as outlined below:

a) If there is strong evidence of an additional inflammatory effect (CRP) and a satisfactory safety profile consider progression to clinical endpoint evaluation whether in this trial or in another one;
b) Terminate arm and do not proceed (based on lack of evidence of an additional biological effect or of an unfavourable safety signal).

Success will be declared if there is a 90% probability that the intervention arm is better than usual care in reducing CRP. Futility is defined as less than 50% probability of the intervention being better than usual care. However, given the large number of agents being investigated in various phase II trials, the size of effect and the totality of data will be reviewed before recommending adoption by a phase III platform. In the event of a successful treatment being identified and the effect size being large, consideration may be given to continuing the arm within CATALYST to study clinical efficacy (based on the WHO scale), if this were deemed to be a more efficient path than translation to a phase III platform. More information, including the operating characteristics based on a simpler analysis of the area under the curve for sequential CRP data are included in Supplementary Appendix 7.

New arms will be added as new interventions become available. Each intervention will be compared to temporally-relevant usual care controls, using only those patients for whom that intervention was a randomisation option. A detailed secondary outcome measure analysis can be found in a predefined Statistical Analysis Plan (Supplementary Appendix 8). The trial statisticians will not be blinded. Exploratory

For peer review only - http://bmjopen.bmj.com/site/about/guidelines.xhtml

subgroup analyses will be conducted to ascertain the effect of treatment on the primary outcome measure within care status strata (ward or ICU) and disease severity (WHO score <6 or ≥6). Analyses will be conducted as per the primary outcome measure with any inference based on the treatment/time interaction covariate included in the model formulation. Efficacy measurements will be performed primarily on a modified intention to treat population that will include all patients who receive treatment and have at least a baseline and one post-treatment measurement. Missing data will not be imputed. The safety population will include all patients in the usual care arm and all patients who receive a trial intervention in the active arms.

Adverse events reporting and analysis

The collection and reporting of AEs will be in accordance with the Research Governance Framework for Health and Social Care and the requirements of the National Research Ethics Service (NRES). Definitions of different types of AEs are listed in Supplementary Appendix 9. The reporting period for AEs will be between the date of commencement of protocol-defined treatment until day 28. The investigator should assess the seriousness and causality (relatedness) of all AEs experienced by the patient (this should be documented in the source data) with reference to the protocol. Abnormal laboratory findings will only be reported if they satisfy one of the following; (a) events which are Grade 3 or above; (b) events which result in the early discontinuation of trial treatment (if applicable to the research arm); or (c) events which result in a dose modification or dose interruption (if applicable to the research arm). Pre-existing conditions and pre-existing abnormal laboratory findings will only be reported if the condition worsens by at least one CTCAE grade. Hospitalisations for pre-planned elective procedures, unless the condition worsens, will not be reported as Serious (S)AEs.

Data management

Data will be collected via a set of forms capturing details of eligibility, baseline characteristics, treatment and outcome details created using FORMAP Case Report Form design software developed by Birmingham Clinical Trials Unit at the University of Birmingham. This trial will use an electronic remote data capture system, with the exception of SAE reporting and notification of pregnancy; these are both paper-based. All trial records must be archived and securely retained for at least 25 years. No documents will be destroyed without prior approval from the sponsor, via the central CATALYST Trial Office. On-site monitoring will be carried out as required following a risk assessment and as documented in the Quality Management Plan. Any monitoring activities will be reported to the central CATALYST Trial Office and any issues noted will be followed up to resolution. CATALYST will also be centrally monitored, which may trigger additional on-site monitoring. The trial management group and authors will have access to the final dataset. Further information regarding data management is provided in the study protocol.

Trial organisation structure

The University of Birmingham will act as single sponsor for this multi-centre study: Support Group, Aston Webb Building, Room 119, Birmingham, B15 2TT. Email: researchgovernance@contacts.bham.ac.uk). The trial is being conducted under the auspices of the Cancer Research UK Clinical Trials Unit (CRCTU), University of Birmingham, in close partnership with the National Institute for Health Research (NIHR) Biomedical Research Centres (BRC) at the Universities of Birmingham and Oxford, University College London (UCL) and Imperial College London.

Given the combination of a novel disease, a range of novel potential therapies and a pandemic setting, a multidisciplinary collaboration was essential, bringing together experts in acute, respiratory and intensive

For peer review only - http://bmjopen.bmj.com/site/about/guidelines.xhtml

care medicine, inflammation, oncology, data sciences, trials methodology and statistics. The Birmingham Acute Care Research group provides a single point of reference for the acute specialities (24).

The trial management group (TMG) is responsible for the day-to-day running and management of the trial. Members include the Chief Investigator, Deputy Chief Investigator, Co-investigators, Trial Statisticians, Trial Management Team Leader and Trial Coordinator. The TMG reports to the TSC.

The TSC provides oversight and governance. Members include independent clinicians and patient advocates. The TSC supervises the conduct of the trial, monitoring progress including recruitment, data completeness, losses to follow-up, and deviations from the protocol. They will make recommendations about conduct and continuation of the trial.

The independent data monitoring committee (DMC) includes clinicians and a statistician who will review unblinded data analyses to advise the TSC on whether the trial data (and results from other relevant research), justifies the continuing recruitment of further patients. The DMC will operate in accordance with a trial-specific charter based on the template created by the Damocles Group. The DMC will review the trial data three-monthly during the recruitment and while patients remain on treatment. These may occur more frequently if the DMC deem necessary or for interventions which have not previously been administered to patients in this specific setting.

The SAB makes recommendations on prioritising interventions and aspects of methodology such as co-enrolment in order to harmonise trial activities with other research platforms. The SAB includes multi-stakeholder representatives (detailed in Supplementary Appendix 1) including, the collaborating centres and the associated Biomedical Research Centres (25) in Birmingham (26), Oxford (27), and University College London (28).

Confidentiality statement

Confidential trial data will be stored in accordance with the General Data Protection Regulation (GDPR) 2018. As specified in the patient information sheet and with the patient's consent, patients will be identified using only their date of birth and unique trial ID number.

Trial status

Recruitment for the trial opened in May 2020 with the namilumab and infliximab arms. Although included in the protocol, the gemtuzumab-ozogamicin arm has not opened to recruitment.

Discussion

CATALYST is a nimble, accelerated, open-label, targeted phase II proof-of-principle multi-arm trial permitting efficient evaluation of repurposed and/or novel drugs to modify the disease progression of COVID-19 in patients admitted to wards and ICU. CATALYST aims to determine suitability of a proposed new treatment for evaluation in phase III national platform trials. CATALYST has an adaptive platform design, aiming to translate laboratory-based research to patients with SARS-CoV-2 infection without delay.

This trial is designed with clinical pressures caused by the pandemic in mind. Outcomes are easy to record, location-independent, and applicable across the spectrum of illness severity. We have also prioritised serial continuous measures over discontinuous or ordinal metrics, as these allow for greater statistical efficiency and thus smaller sample sizes. The primary outcome chosen provides a rapid, biologically-driven efficacy signal to allow early "go/no-go" decisions. While the WHO has adopted a consensus-based set of core outcome measures for studies of SARS-CoV-2 infection (29), our study aim was to develop a trial with a

For peer review only - http://bmjopen.bmj.com/site/about/guidelines.xhtml

smaller sample size to provide earlier signals of potential efficacy for multiple investigational agents, allowing selection of the most promising to be taken forward by larger platforms with clinical outcomes.

We initially considered SpO_2/FiO_2 as a suitable primary outcome for CATALYST. This is an indicator of the severity, progression, or remission of acute lung injury (30). However, as real-world data emerged, we found the relationship between the ratio and outcome was complex and was also susceptible to measurement error in patients receiving ward-based forms of respiratory support. Furthermore, its prognostic utility is compromised if the inspired oxygen concentration is not rapidly adjusted to the patients' needs by the attending staff, which is often delayed in a pandemic. Other shortfalls included high variability between observations, the impact on the measure of switching the mode of oxygen delivery, and microthrombotic events altering SpO_2/FiO_2 through alteration of perfusion (31). We, therefore, evaluated whether CRP would be a better primary outcome.

C-Reactive Protein is produced as a homopentameric protein, termed native CRP (nCRP), which can irreversibly dissociate at sites of inflammation, tissue damage, and infection into five separate monomers, termed monomeric CRP (mCRP) (32). C-Reactive Protein levels are widely used as a marker of infection or inflammation; however, evidence suggests that CRP plays an active role in the inflammatory process (33-38).

During the first wave we modelled data on CRP over time in COVID-19 patients finding that it performed better than SpO_2/FiO_2. This is consistent with published data indicating that baseline and peak CRP, median CRP over time, slope of CRP rise over seven days, and rapid rise during early disease are all associated with outcome in hospitalised patients with COVID-19 (39-42). CRP trends over time tend to have greater predictive power, with change in CRP levels performing better at predicting respiratory failure and subsequent intubation than baseline CRP or respiratory rate-oxygenation (ROX) index (40). In addition, compared to those that die, patients who survive have lower peak CRP levels and earlier reductions (41). Notably, CRP at baseline also correlates with CT grading of lung involvement (43). A recent large study found that CRP ≥40 mg/L, a key entry criteria for our study, was the optimal CRP cut-off for predicting mortality on hospital admission (44).

The recent success of dexamethasone in the treatment of COVID-19 reinforces the relevance of inflammatory pathology to clinical outcomes (45). Methylprednisolone has been associated with CRP reduction over seven days and improved clinical outcomes, in a strategy that continued this steroid until a target CRP, or the ratio of arterial oxygen partial pressure to fractional inspired oxygen (PiO_2/FiO_2) threshold, was reached (46). Although IL-6 blockade has a direct effect on CRP production, which might obscure a relationship between CRP trends and outcome, small studies have suggested a differential effect on CRP decline between clinical responders and non-responders (47, 48).

While CRP may not show complete concordance with clinical outcomes, we argue that if dysregulated inflammation is indeed a key pathogenic driver in severe COVID-19, an immunomodulating drug capable of ameliorating those outcomes is also likely to show early improvement of CRP. Conversely, an immunomodulating drug unable to influence CRP is a less promising candidate to investigate in large phase III trials. Limitations of CRP, however, include lower utility for candidate therapeutics whose mechanism of action is not immunomodulation, and the diminished ability to assess drugs that directly target IL-6, due to the direct pharmacodynamics effects on CRP. This study was designed before use of IL-6 blockade outside of trials, and if tocilizumab were to be widely adopted, adaptive modification of the current trial design may be required to account for this.

In conclusion, the major strength of CATALYST is its ability to provide a rapid readout on safety, and proof-of-efficacy enabling phase III trial resources to be focussed and allocated for drugs with a high likelihood of success (45, 49). This will reduce the time lag in translating early phase drugs into effective therapeutics for COVID-19.

For peer review only - http://bmjopen.bmj.com/site/about/guidelines.xhtml

Ethics and dissemination

The trial will be performed in accordance with the recommendations guiding physicians in biomedical research involving human subjects, adopted by the 18th World Medical Association General Assembly, Helsinki, Finland and stated in the respective participating countries laws governing human research, and Good Clinical Practice. The initial protocol was approved by East Midlands - Nottingham 2 Research Ethics Committee, (REC Ref: 20/EM/0115) on 05-May-2020, with subsequent amendments approved on 28-May-2020 (addition of namilumab and infliximab), 12-Jun-2020 (inclusion change – suspected COVID-19), 20-Jun-2020 (following dexamethasone as standard of care use), 12-Oct-2020 (change of primary outcome to CRP). The MHRA has given its approval of all protocol versions; current version in use is 6.0.

A meeting will be held after the end of the trial to allow discussion of the main results among the collaborators prior to publication. Results of the primary and secondary endpoints will be submitted for publication in peer-reviewed journals. Manuscripts will be prepared by the TMG and authorship will be determined by mutual agreement.

Contributors

Study conception: TV, BAF, DS, AR, RS, DT, TW, MR, DP, DR, JB, PK, SG. Study design: TV, BAF, DS, AR, RS, DT, TW, MR, JS, DP, SJB, JSS, DR, JB, PK, SG. DS is the Trial Biostatistician and SG is the Senior Trial Biostatistician, both were responsible for developing the statistical plan.

Funding

This trial is supported by the Medical Research Council (MRC) grant number MC_PC_20007. SG is supported by a Senior Investigator Award from the National Institute of Health Research. Staff at the CRCTU are supported by core funding grants from Cancer Research UK (C22436/A25354), the NIHR Biomedical Research Centre (BRC-1215-20009), The Kennedy Trust for Rheumatology Research as part of the Arthritis – Trials Acceleration Programme (KENN161704), and Innovate UK as part of the Midlands – Wales Advanced Therapy Treatment Centres (104232). This paper presents independent research supported by the NIHR Birmingham Biomedical Research Centres at the University Hospitals Birmingham NHS Foundation Trust and the University of Birmingham, as well as Oxford and University College London Hospitals Biomedical Research Centres. The views expressed are those of the authors and not necessarily those of the NHS, the NIHR or the Department of Health and Social Care. Namilumab is being provided free of charge by Izana Bioscience, Oxford, UK (now part of Roivant). Infliximab is being provided free of charge by Celltrion.

Role of funders and sponsor

Neither the sponsor or funders had any role in trial design, data collection, data analysis, data interpretation or writing of the report. The corresponding author had full access to all the data in the trial and had final responsibility for the decision to submit for publication.

Competing interests

BAF has undertaken consultancy for Novartis, BMS, Servier, Galapagos and Janssen; MR is currently undertaking a Senior Clinical Fellowship financed by Roche; PK has undertaken consultancy for BMS, AstraZeneca, and AbbVie; all are unrelated to this trial. All other authors declare no competing interests.

For peer review only - http://bmjopen.bmj.com/site/about/guidelines.xhtml

For peer review only

Acknowledgements

We thank staff from the CRCTU, University of Birmingham including Dr Siân Lax for contributions to the paper.

References

1. Hall M, Pritchard M, Dankwa EA, Baillie JK, Carson G, Citarella BW, et al. ISARIC Clinical Data Report 20 November 2020. medRxiv. 2020:2020.07.17.20155218.

2. ICNARC Report on COVID-19 in Critcal Care: England, Wales and Northern Ireland, 29 January 2021 [Available from: https://www.icnarc.org/Our-Audit/Audits/Cmp/Reports.

3. Huang C, Huang L, Wang Y, Li X, Ren L, Gu X, et al. 6-month Consequences of COVID-19 in Patients Discharged from Hospital: A Cohort Study. The Lancet. 2021;397(10270):220-32.

4. Raman B, Cassar MP, Tunnicliffe EM, Filippini N, Griffanti L, Alfaro-Almagro F, et al. Medium-term Effects of SARS-CoV-2 Infection on Multiple Vital Organs, Exercise Capacity, Cognition, Quality of Life and Mental Health, Post-hospital Discharge. EClinicalMedicine. 2021;31:100683.

5. Ackermann M, Verleden SE, Kuehnel M, Haverich A, Welte T, Laenger F, et al. Pulmonary Vascular Endothelialitis, Thrombosis, and Angiogenesis in Covid-19. New England Journal of Medicine. 2020;383(2):120-8.

6. Leisman DE, Ronner L, Pinotti R, Taylor MD, Sinha P, Calfee CS, et al. Cytokine Elevation in Severe and Critical COVID-19: A Rapid Systematic Review, Meta-analysis, and Comparison with other Inflammatory Syndromes. The Lancet Respiratory Medicine. 2020;8(12):1233-44.

7. Huang C, Wang Y, Li X, Ren L, Zhao J, Hu Y, et al. Clinical Features of Patients Infected with 2019 Novel Coronavirus in Wuhan, China. The Lancet. 2020;395(10223):497-506.

8. Liao M, Liu Y, Yuan J, Wen Y, Xu G, Zhao J, et al. Single-cell Landscape of Bronchoalveolar Immune Cells in Patients with COVID-19. Nature Medicine. 2020;26(6):842-4.

9. Prompetchara E, Ketloy C, Palaga T. Immune Responses in COVID-19 and Potential Vaccines: Lessons Learned from SARS and MERS Epidemic. Asian Pacific Journal of Allergy and Immunology. 2020;38:1-9.

10. Randomised Evaluation of COVID-19 Therapy (RECOVERY) Trial Protocol 2020 [Available from: https://www.recoverytrial.net/for-site-staff.

11. Randomized, Embedded, Multifactorial Adaptive Platform trial for CommunityAcquired Pneumonia (REMAP-CAP): Core Protocol 2020 [Available from: https://www.remapcap.org/protocol-documents.

12. Dean NE, Gsell P-S, Brookmeyer R, Crawford FW, Donnelly CA, Ellenberg SS, et al. Creating a Framework for Conducting Randomized Clinical Trials during Disease Outbreaks. New England Journal of Medicine. 2020;382(14):1366-9.

13. Kieny MP, Salama P. WHO R&D Blueprint: a global coordination mechanism for R&D preparedness. Lancet. 2017;389(10088):2469-70.

14. Health Research Authority Consent and Participant Information Guidance [Available from: http://www.hra-decisiontools.org.uk/consent/principles-ALC-EnglandandWales.html.

15. Papp KA, Gooderham M, Jenkins R, Vender R, Szepietowski JC, Wagner T, et al. Granulocyte-macrophage Colony-stimulating Factor (GM-CSF) as a Therapeutic Target in Psoriasis: Randomized, Controlled Investigation using Namilumab, a Specific Human anti-GM-CSF Monoclonal Antibody. British Journal of Dermatology. 2019;180(6):1352-60.

16. Tanaka S, Harada S, Hiramatsu N, Nakaya R, Kawamura M. Randomized, double-blind, placebo-controlled, phase I study of the safety and pharmacokinetics of namilumab in healthy Japanese and Caucasian menInternational Journal of Clinical Pharmacology and Therapeutics. 2018;56(11):507-17.

For peer review only - http://bmjopen.bmj.com/site/about/guidelines.xhtml

17. Huizinga TW, Batalov A, Stoilov R, Lloyd E, Wagner T, Saurigny D, et al. Phase 1b Randomized, Double-blind Study of Namilumab, an anti-Granulocyte Macrophage Colony-stimulating Factor Monoclonal Antibody, in Mild-to-moderate Rheumatoid Arthritis. Arthritis Research & Therapy. 2017;19(1):53.

18. Taylor PC, Saurigny D, Vencovsky J, Takeuchi T, Nakamura T, Matsievskaia G, et al. Efficacy and Safety of Namilumab, a Human Monoclonal Antibody Against Granulocyte-macrophage Colony-stimulating Factor (GM-CSF) Ligand in Patients with Rheumatoid Arthritis (RA) with Either an Inadequate Response to Background Methotrexate Therapy or an Inadequate Response or Intolerance to an anti-TNF (Tumour Necrosis Factor) Biologic Therapy: A Randomized, Controlled Trial. Arthritis Research & Therapy. 2019;21(1):101.

19. Robinson PC, Richards D, Tanner HL, Feldmann M. Accumulating Evidence Suggests anti-TNF Therapy Needs to be Given Trial Priority in COVID-19 Treatment. The Lancet Rheumatology. 2020;2(11):e653-e5.

20. Williamson PR, Altman DG, Bagley H, Barnes KL, Blazeby JM, Brookes ST, et al. The COMET Handbook: version 1.0. Trials. 2017;18(3):280.

21. Core Outcome Measures in Effectiveness Trials [Available from: http://www.comet-initiative.org/.

22. Cancer Therapy Evaluation Program. Common Terminology Criteria for Adverse Events (CTCAE), Version 4.03. US Department of Health and Human Services, NIH, NCI. 2010.

23. ISARIC/WHO Clinical Characterisation Protocol for Severe Emerging Infections 2020 [Available from: https://isaric.tghn.org/clinical-characterisation-protocol-ccp/.

24. University of Birmingham Acute Care Research [Available from: https://www.birmingham.ac.uk/research/inflammation-ageing/acute-care-research.aspx.

25. National Institute for Health Research Biomedical Research Centres [Available from: https://www.nihr.ac.uk/explore-nihr/support/experimental-medicine.htm.

26. National Institute for Health Research Birmingham Biomedical Research Centre [Available from: https://www.birminghambrc.nihr.ac.uk/.

27. National Institute for Health Research Oxford Health Biomedical Research Centre [Available from: https://oxfordhealthbrc.nihr.ac.uk/.

28. National Institute for Health Research University College London Hospitals Biomedical Research Centre [Available from: https://www.uclhospitals.brc.nihr.ac.uk/content/biomedical-research-centre.

29. Marshall JC, Murthy S, Diaz J, Adhikari NK, Angus DC, Arabi YM, et al. A Minimal Common Outcome Measure Set for COVID-19 Clinical Research. The Lancet Infectious Diseases. 2020;20(8):e192-e7.

30. The ARDS Definition Task Force. Acute Respiratory Distress Syndrome: The Berlin Definition. JAMA. 2012;307(23):2526-33.

31. Vlaar APJ, de Bruin S, Busch M, Timmermans S, van Zeggeren IE, Koning R, et al. Anti-C5a Antibody IFX-1 (Vilobelimab) Treatment versus Best Supportive Care for Patients with Severe COVID-19 (PANAMO): An Exploratory, Open-label, Phase 2 Randomised Controlled Trial. The Lancet Rheumatology. 2020;2(12):e764-e73.

32. Sproston NR, Ashworth JJ. Role of C-Reactive Protein at Sites of Inflammation and Infection. Front Immunol. 2018;9:754-.

33. Thiele JR, Habersberger J, Braig D, Schmidt Y, Goerendt K, Maurer V, et al. Dissociation of Pentameric to Monomeric C-reactive Protein Localizes and Aggravates Inflammation: In Vivo Proof of a Powerful Proinflammatory Mechanism and a New Anti-inflammatory Strategy. Circulation. 2014;130(1):35-50.

34. Khreiss T, József L, Hossain S, Chan JS, Potempa LA, Filep JG. Loss of Pentameric Symmetry of C-reactive Protein is Associated with Delayed Apoptosis of Human Neutrophils. Journal of Biological Chemistry. 2002;277(43):40775-81.

35. Singh U, Devaraj S, Vasquez-Vivar J, Jialal I. C-reactive Protein Decreases Endothelial Nitric Oxide Synthase Activity via Uncoupling. Journal of Molecular and Cellular Cardiology. 2007;43(6):780-91.

36. Krayem I, Bazzi S, Karam M. The Combination of CRP Isoforms with oxLDL Decreases TNF-α and IL-6 Release by U937-derived Macrophages. Biomed Rep. 2017;7(3):272-6.

For peer review only

For peer review only - http://bmjopen.bmj.com/site/about/guidelines.xhtml

37. Khreiss T, József L, Potempa LA, Filep JG. Loss of Pentameric Symmetry in C-reactive Protein Induces Interleukin-8 Secretion Through Peroxynitrite Signaling in Human Neutrophils. Circulation Research. 2005;97(7):690-7.

38. Han KH, Hong KH, Park JH, Ko J, Kang DH, Choi KJ, et al. C-reactive Protein Promotes Monocyte Chemoattractant Protein-1 - Mediated Chemotaxis through Upregulating CC Chemokine Receptor 2 Expression in Human Monocytes. Circulation. 2004;109(21):2566-71.

39. Luo X, Zhou W, Yan X, Guo T, Wang B, Xia H, et al. Prognostic Value of C-Reactive Protein in Patients With Coronavirus 2019. Clinical Infectious Diseases. 2020;71(16):2174-9.

40. Mueller AA, Tamura T, Crowley CP, DeGrado JR, Haider H, Jezmir JL, et al. Inflammatory Biomarker Trends Predict Respiratory Decline in COVID-19 Patients. Cell Reports Medicine. 2020;1(8):100144.

41. Sharifpour M, Rangaraju S, Liu M, Alabyad D, Nahab FB, Creel-Bulos CM, et al. C-reactive Protein as a Prognostic Indicator in Hospitalized Patients with COVID-19. PLoS One. 2020;15(11):e0242400.

42. Malik P, Patel U, Mehta D, Patel N, Kelkar R, Akrmah M, et al. Biomarkers and Outcomes of COVID-19 Hospitalisations: Systematic Review and Meta-analysis. BMJ Evidence Based Medicine. 2020:doi: 10.1136/bmjebm-2020-111536.

43. Tan C, Huang Y, Shi F, Tan K, Ma Q, Chen Y, et al. C-reactive Protein Correlates with Computed Tomographic Findings and Predicts Severe COVID-19 Early. Journal of Medical Virology. 2020;92(7):856-62.

44. Stringer D, Braude P, Myint PK, Evans L, Collins JT, Verduri A, et al. The role of C-reactive protein as a prognostic marker in COVID-19. International Journal of Epidemiology. 2021;50(2):420-9.

45. Horby P, Lim WS, Emberson JR, Mafham M, Bell JL, Linsell L, et al. Dexamethasone in Hospitalized Patients with Covid-19 - Preliminary Report. New England Journal of Medicine. 2020:doi: 10.1056/NEJMoa2021436.

46. Salton F, Confalonieri P, Meduri GU, Santus P, Harari S, Scala R, et al. Prolonged Low-Dose Methylprednisolone in Patients With Severe COVID-19 Pneumonia. Open Forum Infectious Diseases. 2020;7(10):ofaa421.

47. Montesarchio V, Parrela R, Iommelli C, Bianco A, Manzillo E, Fraganza F, et al. Outcomes and Biomarker Analyses Among Patients with COVID-19 Treated with Interleukin 6 (IL-6) Receptor Antagonist Sarilumab at a Single Institution in Italy. Journal for Immunotherapy of Cancer. 2020;8(2):e001089.

48. Jordan SC, Zakowski P, Tran HP, Smith EA, Gaultier C, Marks G, et al. Compassionate Use of Tocilizumab for Treatment of SARS-CoV-2 Pneumonia. Clinical Infectious Diseases. 2020;71(12):3168-73.

49. RECOVERY Collaborative Group. Azithromycin in Patients Admitted to Hospital with COVID-19 (RECOVERY): A Randomised, Controlled, Open-label, Platform Trial. The Lancet. 2021:doi: 10.1016/S0140-6736(21)00149-5.

For peer review only

For peer review only - http://bmjopen.bmj.com/site/about/guidelines.xhtml

Table 1 World Health Organization Clinical Progression Scale

Patient State	Descriptor	Score
Uninfected	Uninfected; no viral RNA detected	0
Ambulatory	Asymptomatic; viral RNA detected	1
	Symptomatic; independent	2
	Symptomatic; assistance needed	3
Hospitalised; mild disease	Hospitalised; no oxygen therapy	4
	Hospitalised; oxygen by mask or nasal prongs	5
Hospitalised; severe disease	Hospitalised; oxygen by NIV or high flow	6
	Intubated and mechanical ventilation, $pO_2/FiO_2 \geq 150$ or $SpO_2/FiO_2 \geq 200$	7
	Mechanical ventilation pO_2/FiO_2 <150 (SpO_2/FiO_2 <200) **or** vasopressors	8
	Mechanical ventilation pO_2/FiO_2 <150 (SpO_2/FiO_2 <200) **and** vasopressors, dialysis or ECMO	9
Death	Dead	10

Adapted from Marshall et al., 2020 (29).

Footnotes for use in CATALYST

1. If pO_2 not available then use the SpO_2/FiO_2 ratio instead

2. For pO_2 measurements in kPa, use an online calculator e.g., https://www.msdmanuals.com/en-gb/medical-calculators/PaO2_FiO2Ratio.htm to calculate a pO_2/FiO_2 ratio equivalent to that obtained with pO_2 measured in mmHg, or else consider an equivalent ratio to 200, when dividing pO_2 in kPa by FiO_2, is 26.7, and an equivalent to 150 is 20.

3. If medically fit for discharge, record status as for ambulatory patient

4. Asymptomatic implies a return to baseline symptomatic state i.e., no fever, and no cough, shortness of breath, confusion, myalgia, diarrhoea, fatigue, or weakness above what the participant would have experienced on a daily basis before their COVID-19 episode

5. Symptomatic but independent, implies that the participant has some of the additional symptoms as above, but needs no additional help with activities of daily living above what they required prior to their COVID-19 episode.

6. Symptomatic but needs assistance, implies that in addition to having symptoms as above, they require help with activities of daily living i.e. bathing/showering, personal hygiene and combing of hair, dressing, toileting, mobility/transferring and self-feeding, above what they required on a daily basis prior to their COVID-19 episode.

7. Score 0 (uninfected: no viral RNA detected) is not being assessed as part of CATALYST.

For peer review only

For peer review only - http://bmjopen.bmj.com/site/about/guidelines.xhtml

Table 2 CATALYST Schedule of Events

	Baseline	Day 1	Day 2	Day 3	Day 4	Day 5	Day 6	Day 7	Day 8	Day 9	Day 10	Day 11	Day 12	Day 13	Day 14	Day 15 – Day 27 ¤	Day 28⌂
Eligibility assessment	x																
Consent	x																
Weight/ height (estimated; BSA calculation)	x																
Demographics ±	x																
Pregnancy test *(females only)*	x																
Frailty Score, Co-morbidity assessment	x																
Review of medical history	x																
Randomisation	x																
World Health Organization clinical progression scale	x	x	x	x	x	x	x	x	x	x	x	x	x	x	x	x	x
Routine blood tests *	x	x	[x]	x	[x]	x	[x]	x	{x}	x	[x]	[x]	[x]	[X]	x		
Research Samples≠ *optional – see section 8*		x		x						x$^{\Sigma}$							
National Early Warning Score (NEWS-2)	x	x	x	x	x	x	x	x	x	x	x	x	x	x	x		
FiO_2 levels, O_2 saturations, respiratory rate ¥	x	x	x	x	x	x	x	x	x	x	x	x	x	x	x		
Body temperature, pulse rate	x	x	x	x	x	x	x	x	x	x	x	x	x	x	x		
Adverse event review								x							x		x
Concomitant medication review	x																
IMP Administration **See additional schedules in Supplementary Appendix 6**		x															

¤ Information to be collected at day 28 if patient discharged. A collection tool will be available.

For peer review only - http://bmjopen.bmj.com/site/about/guidelines.xhtml

⌂ Information on Serious Adverse Events (SAEs) will be collected until 28 days after the last IMP administration, which may be after this time point.

± To include age, sex and smoking status (if known).

* Full blood count (WBC, platelets, lymphocytes, neutrophils, monocytes, eosinophils, haemoglobin), D-Dimer, C-reactive protein (CRP), ferritin, lactate dehydrogenase (LDH), , liver function test (alanine aminotransferase (ALT) or aspartate aminotransferase (AST), bilirubin, alkaline phosphatase (ALP), albumin), urea and electrolytes (U&Es; urea, creatinine, sodium and potassium) NB. On day of IMP administration, this should be taken pre (up to 24hrs earlier). [x] – not mandatory for usual care but if undertaken for clinical or safety reasons, data will be collected.

∑ Or on day of discharge if earlier.

≠ The Sample Sub-study is only open at specific sites. Samples can be taken +/- 24 hrs of Day 1. On Day 3, samples can be taken up to 24 hrs before and up to 48 hrs after the visit. Day 9 samples can be taken up to 24 hrs before and up to 48 hrs after the visit OR at discharge if earlier. Please note the samples should be taken before IMP administration on Day 1 where possible.

¥ O_2 and saturation levels will be recorded in line with the standard of care: approximately every 4hrs in Intensive Care and 4-6 hourly on the ward.

Notes:

If the patient is discharged from the hospital before their next scheduled visit, the participant should be provided with the WHO clinical improvement scale diary and the visits on days 7, 14 and 28 should take place by telephone (if it is not possible to see the patient). If the visit is via telephone please perform an adverse event review including asking about any hospitalisations and a WHO clinical improvement scale assessment.

On Day 1 tests and interventions should be recorded pre-dose (if randomised to interventional arms).

For peer review only

For peer review only - http://bmjopen.bmj.com/site/about/guidelines.xhtml

Figure 1 CATALYST Trial Schema

The gemtuzumab-ozogamicin arm has been de-prioritised and is not open to recruitment.

AE, adverse events; CRP, C-reactive protein; NEW-2, National Early Warning Scale 2; WHO, World Health Organization

For peer review only

For peer review only - http://bmjopen.bmj.com/site/about/guidelines.xhtml

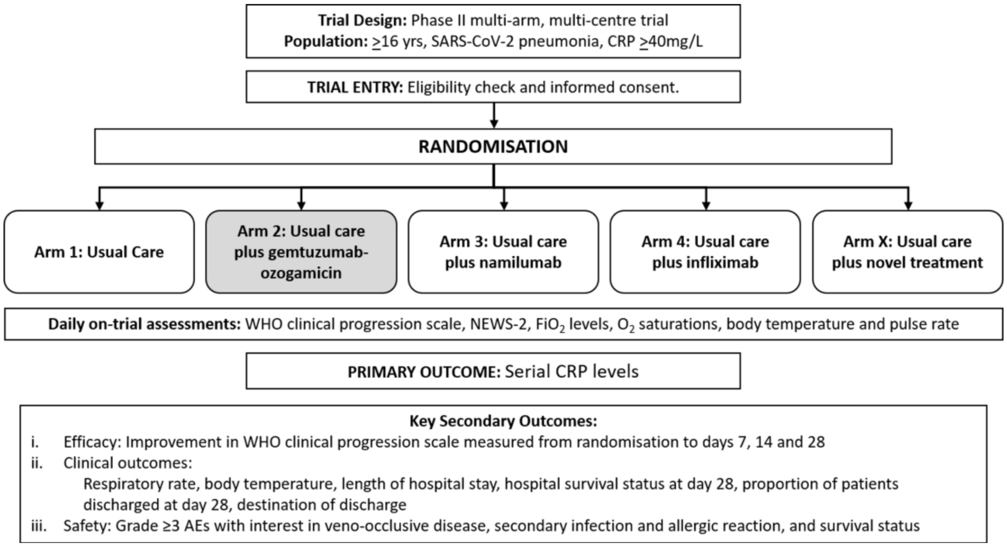

Figure 1 CATALYST Trial Schema. The gemtuzumab-ozogamicin arm has been de-prioritised and is not open to recruitment. AE, adverse events; CRP, C-reactive protein; NEW-2, National Early Warning Scale 2; WHO, World Health Organisation

203x109mm (300 x 300 DPI)

Supplementary Appendix 1 – CATALYST investigators and sites

The CATALYST investigators include the following;

Chief Investigator Prof. Tonny Veenith (University Hospitals Birmingham), deputy Chief Investigator Dr Benjamin Fisher (University of Birmingham), Dr Francis Mussai (University of Birmingham), Prof. Gary Middleton (University of Birmingham), Dr Dhruv Parekh (University of Birmingham), Dr Anna Rowe (Cancer Research UK Clinical Trials Unit (CRCTU), University of Birmingham), Prof. Duncan Richards (University of Oxford), Dr Mathew Rowland (University of Oxford), Prof. Julian Bion (University of Birmingham), Daniel Slade (CRCTU, University of Birmingham), Prof. Simon Gates (CRCTU, University of Birmingham), Prof. Pam Kearns (CRCTU, University of Birmingham), Dr Rowena Sharpe (CRCTU, University of Birmingham), Dr Sarah Bowden (CRCTU, University of Birmingham), Prof. David Thickett (University Hospitals Birmingham), Prof. Julian Bion (University of Birmingham), Dr Tony Whitehouse (University Hospitals Birmingham), Dr James Scriven (University Hospitals Birmingham), Dr Mansoor Bangash (University Hospitals Birmingham), Prof. Fang Gao-Smith (University Hospitals Birmingham), Dr Jaimin Patel (University Hospitals Birmingham), Prof. Elizabeth Sapey (University Hospitals Birmingham), Prof. Mark Coles (University of Oxford), Prof. Peter Watkinson (University of Oxford), Prof. Naj Rahman (University of Oxford), Prof. Ling-Pei Ho (University of Oxford), Prof. Brian Angus (University of Oxford), Dr Alex Mentzer (University of Oxford), Dr Alex Novak (University of Oxford), Prof. Marc Feldmann (University of Oxford).

CRCTU staff: Charlotte Gaskell, Camilla Bathurst, Joseph van de Wiel, Alex Vince, Lili Evans, Rhian Jones, Karan Kaliri, Susie Mee, Karen James, Bushra Rahman, Karen Turner.

Trial Steering Committee: Prof. Michael Matthay (Chair), Prof. Danny McAuley (Deputy Chair), Prof. Paul Dark, Prof. Sir Andrew McMichael, Andrew Hall (Independent Statistician), Simon Farrell (PPI representative), Hannah Farrell (PPI representative)

Independent Data Management Committee: Prof. Adam Hill (Chair), Prof. Christina Yap (Independent Statistician), Prof. Anthony Gordon.

Scientific Advisory Board: Prof. Philip Newsome (Chair; Birmingham BRC Deputy Director, Clinician), Prof. Helen McShane (Deputy Chair, Oxford BRC Director, Clinician), Prof. Graham Cooke (Imperial College BRC Representative, Clinician), Prof. Duncan Richards (Director of Oxford CTRU, Clinician), Prof. Bryan Williams (UCL BRC Director, Clinician), Prof. Ling-Pei Ho (Translational Research Collaborative Representative, Clinician), Prof. Tonny Veenith (Chief Investigator, Clinician), Dr Ben Fisher (Deputy Chief Investigator, Clinician), Prof. Vincenzo Libri (CRF Representative, Clinician), Prof. Julian Bion (Clinical Trials Oversight Committee Representative, Clinician), Prof. Pamela Kearns (Trial Sponsor Representative, Director of CRCTU, Clinician), Dr Rowena Sharpe (Trial Management Group Representative), Prof. Simon Gates (Senior Biostatistician, CRCTU), Dr Rebecca Turner (Statistician).

CATALYST sites: Queen Elizabeth, Birmingham; John Radcliffe, Oxford; Imperial Charing Cross, London; Imperial Hammersmith, London; Imperial St Mary's, London; Royal Bolton Hospital, Bolton; Good Hope Hospital, Birmingham; Heartlands Hospital, Birmingham; University Hospital of Wales, Cardiff; University College London Hospital, London; Morriston Hospital, Swansea; The Royal Hallamshire Hospital, Sheffield.

For peer review only - http://bmjopen.bmj.com/site/about/guidelines.xhtml

Supplementary Appendix 2 – CATALYST WHO dataset

Data category	Information
Primary registry and trial identifying number	EudraCT Number 2020-001684-89
Date of registration in primary registry	15-May-2020
Secondary identifying numbers	ISRCTN: 40580903
Source(s) of monetary or material support	Medical Research Council
Primary sponsor	University of Birmingham
Secondary sponsor(s)	n/a
Contact for public queries	TV: t.v.veenith@bham.ac.uk<br>BF: b.fisher@bham.ac.uk
Contact for scientific queries	TV: t.v.veenith@bham.ac.uk<br>BF: b.fisher@bham.ac.uk
Public title	Which treatment could lessen the severity of a coronavirus infection when compared with usual care in an NHS setting?
Scientific title	A multicentre, open-label, phase II, multi-arm trial for an early and accelerated evaluation of the potential treatments for COVID-19 in hospitalised adults
Countries of recruitment	UK
Health condition(s) or problem(s) studied	COVID-19
Intervention(s)	Usual care provided following the current institutional policy for patients with COVID-19
	Usual care combined with namilumab
	Usual care combined with infliximab

For peer review only - http://bmjopen.bmj.com/site/about/guidelines.xhtml

CATALYST

Data category	Information
Key inclusion and exclusion criteria	Ages eligible for study: ≥16 years<br>Sexes eligible for study: both<br>Accepts healthy volunteers: no
	Inclusion criteria: adult patient (≥ 16 years), patient hospitalised with SARS-CoV-2 pneumonia
	Exclusion criteria: allergy against namilumab or infliximab, pregnancy or breastfeeding women, tuberculosis or other severe (non-SARS-CoV-2) infections
Study type	Interventional
	Allocation: randomised, open-label
	Primary purpose: safety and biological signal for efficacy
	Phase II
Date of first enrolment	May-2020
Target sample size	Up to 60 per arm
Recruitment status	Closed
Primary outcome(s)	C-reactive protein concentration over time (time frame: 28 days)
Key secondary outcome	WHO Clinical Progression Improvement Scale (time frame: 28 days)

For peer review only - http://bmjopen.bmj.com/site/about/guidelines.xhtml

Supplementary Appendix 3 – Summary of CATALYST protocol changes

Amendment number	Date of approval	Protocol version number	Type of amendment	Summary of amendment
1	**REC:**<br>14-May-20	n/a	Substantial Amendment	Addition of Oxford and UCL as sites
2	**MHRA:**<br>29-May-20<br>**HRA:**<br>01-Jun-20	3.0	Substantial Amendment	Addition of two new IMPs: Namilumab and Infliximab.<br>Update SOE, amendments to inclusion/ exclusion criteria.<br>Specifically: New exclusion criteria relating to the addition of the new drugs:<br>1) Known hypersensitivity to drug products or excipients<br>2) Patients with tuberculosis or other severe infections such as (non-COVID-19) sepsis, abscesses, and opportunistic infections requiring treatment<br>3) Patients with moderate or severe heart failure (NYHA class III/IV)
3	**REC:**<br>10-Jun-20	n/a	Substantial Amendment	Addition of new sites
4	**MHRA:**<br>08-Jun-20	n/a	Substantial Amendment	IMPD update
5	**MHRA:**<br>12-Jun-20<br>**REC:**<br>12-Jun-20	4.0	Substantial Amendment	Amendment to inclusion criteria. Specifically:<br>Inclusion criterion 1 changed to:<br>'Hospitalised adult (≥16 yrs) patients with a clinical picture strongly suggestive of SARS-CoV-2 pneumonia (confirmed by chest X-ray or CT scan, with or without a positive reverse transcription polymerase chain reaction [RT-PCR] assay)' in order to:<br>• Allow CT imaging as evidence for COVID-19 pneumonia<br>• Allow recruitment of patients with strong clinical suspicion for COVID-19 pneumonia but with negative PCR assay
6	**MHRA:**<br>19-Jun-20<br>**REC:**<br>20-Jun-20	5.0	Substantial Amendment	Non-substantial amendments to Sample Collection Sub-study text.<br>Amendment to exclusion criteria.<br>Specifically:<br>'Concurrent immunosuppression with biological agents or prednisone dose > 20mg'<br>Was changed to<br>'Concurrent immunosuppression with biological agents' in order to allow patients to be recruited on dexamethasone, following the RECOVERY data

For peer review only

For peer review only - http://bmjopen.bmj.com/site/about/guidelines.xhtml

CATALYST

Amendment number	Date of approval	Protocol version number	Type of amendment	Summary of amendment
7	**MHRA:**<br>12-Oct-20<br>**REC:**<br>12-Oct-20	6.0	Substantial Amendment	Change of Primary and Secondary Outcomes<br>Specifically:<br>Primary outcome changed to CRP (previously a secondary outcome) from the oxygen saturation to fractional inspired oxygen concentration (SpO2/FiO2) ratio, which now becomes a secondary outcome<br>Hospital free days added as a secondary outcome<br>Overall survival listed as a safety measure (previously death included under hospital survival status as a clinical outcome)<br><br>Applicable changes to Inclusion/ Exclusion Criteria<br>Specifically:<br>Inclusion criteria changed from<br>'Oxygen saturation (SaO2) of ≤94% while breathing ambient air or a ratio of the partial pressure of Oxygen (PaO2) to the fraction of inspired oxygen (FiO2) (PaO2:FiO2) ≤ 300 mg Hg (≤40kPa'), to 'CRP ≥40'<br>The following exclusion criteria that relate to the unopened Myelotarg arm were removed from general exclusion and made arm specific:<br>• Known veno-occlusive disease<br>• Neutrophil count < 2 x 109/l or White Blood Cell Count < 4.0 x 109/l<br>The following exclusion criteria was removed as it was felt to be unnecessarily hindering recruitment:<br>• Chronic Obstructive Pulmonary Disease (known FEV1 < 50% predicted or ambulatory or long term oxygen therapy<br><br>Inclusion of Abbreviations list and eCRF table<br>Update to Statistical Analysis section<br>• Justification for CRP, operating characteristics and decision rules

For peer review only - http://bmjopen.bmj.com/site/about/guidelines.xhtml

<To be printed on hospital headed paper>

CATALYST - An early phase platform trial in (suspected) COVID-19

Which treatment could lessen the severity of a (suspected or confirmed) COVID-19 infection when compared with usual care in an NHS setting?

Patient Information Sheet

For peer review only

Invitation to take part

CATALYST is a clinical trial being led by doctors and researchers based at the University of Birmingham and University Hospitals Birmingham NHS Foundation Trust, and in collaboration with the University of Oxford. You do not have to take part. Taking part is entirely voluntary, and you should only participate if you want to. Choosing not to take part in this trial will not affect your care in any way.

Before you decide whether you want to take part, it is essential for you to understand why the research is being done and what your participation will involve.

Please take time to read the following information carefully and ask your doctor if there is anything that is not clear or if you would like more information.

The purpose of the trial

There is currently no vaccine and few effective treatments for COVID-19. As COVID-19 is a new illness, we are constantly learning more about how it affects the human body. We know that the COVID-19 virus affects a number of different cells in your body, including a type of blood cell called a macrophage (immune cell), and that it can cause the number of these cells to increase in your body. To fight an infection, your immune cells produce proteins called cytokines and chemokines. These proteins can cause inflammation and at high levels can lead to damage in the tissues and organs in your body. Researchers believe this is why some people with COVID-19 infection become very ill.

Therapies we are testing:

A new, unlicensed drug called namilumab which has been tested in patients with arthritis and other inflammatory conditions. It may reduce inflammation in the body caused by the coronavirus. It is currently being given to patients with COVID-19 in a clinical trial in Italy. Namilumab is being provided free of charge by Izana Bioscience Limited (now part of Roivant Sciences) for use in this trial.

For peer review only - http://bmjopen.bmj.com/site/about/guidelines.xhtml

A drug called infliximab (Remsima) which is widely used to treat arthritis and other conditions may reduce inflammation in the body caused by the coronavirus. Infliximab is being provided free of charge by Celltrion Healthcare UK Limited for use in this trial.

Once this inflammation has been reduced, it may be possible that your immune system will adapt and fight off the virus more effectively.

Infliximab has not been tested in patients with COVID-19, and namilumab has only been given to a limited number of patients in Italy, therefore testing these drugs is the purpose of this trial. If any of the drugs benefit people with a (suspected or confirmed) COVID-19 infection in this trial, the drug will then be included in another larger-scale clinical trial being conducted throughout the UK, which is designed to compare treatments to find out which is the best at treating this infection.

This is a national trial, and some hospitals running it are unable to offer patients all of the therapies available within the trial. Your doctor will tell you which treatments are available at your hospital, and if applicable, which ones are not available.

Why have I been invited?

You have been invited to take part in this trial because you are aged 16 years or older, you have been admitted to hospital, and your doctor believes you have a COVID -19 infection and you have higher levels of C-reactive protein in your blood than normal (this increases when there's an infection or inflammation in your body).

What will happen to me if I take part?

- You will be agreeing to take part in a clinical trial and will be actively monitored for 28 days and longer if necessary.
- This clinical trial has three treatment groups or 'arms': one for each of the treatments listed above, and the other arm is 'usual care'. Usual care means that you will receive exactly the same treatment that you would receive in the hospital whether you decide to take part in the trial or not.
- The treatment you will receive is randomly allocated (chosen by chance) by a computer. Neither you nor your doctor will be able to choose the treatment you receive. Your research doctor or nurse will inform you of which treatment you will receive once they have entered you into the trial.
- If you are allocated the 'usual care' arm, you will receive the same medical care as all other patients being treated for a (suspected or confirmed) COVID-19 infection.

For peer review only - http://bmjopen.bmj.com/site/about/guidelines.xhtml

- If you are allocated to receive namilumab, you will receive this in addition to the usual medical care received by patients who have this disease. Namilumab will be given to you through a drip in a vein, usually in your arm, on one occasion only.
- If you are allocated to infliximab, you will receive this in addition to the usual medical care received by patients who have this disease. Infliximab will be given to you through a drip in a vein, usually in your arm, on one occasion only.
- You will have routine blood samples whilst you are in the hospital as part of 'usual care.' If you are allocated to the namilumab or infliximab arm, you may have more blood samples taken regularly before and during treatment to check it is safe for you to receive treatment.
- If you are a female of child bearing potential, a pregnancy test will be performed as part of the screening tests.
- We would also like your permission to collect samples for research. The collection of these samples is optional. This will only take place at certain hospitals. Your doctor will tell you if research samples are being collected at your hospital. This includes collecting samples of blood, and may include swabs from your nose and throat, and if you are placed on a ventilator, samples of the secretions from the tube placed in your windpipe to help you breathe may be taken. Samples will be collected on up to three separate occasions. These samples will be used in laboratory studies to gain a better understanding of COVID-19 and how patients respond to treatment.
- While you are in hospital your health and wellbeing will be monitored in accordance with usual care.

What are the potential benefits of taking part?

This research has been designed to help develop treatment for future patients with suspected or confirmed COVID-19 infection. It is important to understand that you may not directly benefit from taking part in this trial. The benefits for you as an individual are unknown.

What are the possible disadvantages and risks of taking part?

This trial is testing a new way of treating suspected or confirmed COVID-19 infection and you may have side effects from the treatment while taking part in the trial.

All of the treatments have been given to humans before but not in suspected or confirmed COVID-19 infection so there may be side effects that your doctors are not currently aware of, and these may be serious because of this an independent committee will be monitoring the safety of the treatments in this trial on a regular basis.

Everyone taking part in the trial will be monitored carefully for side effects. However, the doctors do not know all the side effects that may occur and we don't know how the drugs used in this trial will interact with the

For peer review only - http://bmjopen.bmj.com/site/about/guidelines.xhtml

other drugs being used to treat (suspected or confirmed) COVID-19. Side effects may be mild or serious or may even be life-threatening. The doctors may give you medicines to help lessen side effects or the trial treatment may be postponed or stopped, depending on the side-effects they may experience.

Specific information is provided below for each of the trial treatments available in the trial is included below:

Namilumab

Namilumab is a new drug that is being tested to treat diseases that cause inflammation such as rheumatoid arthritis.

The side effects that are known so far are:

- Low neutrophil count (a type of white blood cell)
- Minor symptoms such as runny nose and headache
- Tachycardia (a fast heart beat)*

* A short change in heart rhythm was seen with no apparent harm in one person receiving namilumab in another trial.

Infliximab

Infliximab has been widely used for rheumatoid arthritis and other conditions for 20 years but it has some potential side effects. It has been used to treat patients with sepsis (serious infection) before on intensive care units and was demonstrated to be safe. The more common known side effects of infliximab when used to treat other conditions such as arthritis have been summarised below:

While you are receiving Infliximab:

Common side effects (experienced by 1 in 100 people) include:

- Allergic reaction: this can be mild but is sometimes severe and may even be life-threatening. For this reason you will be very carefully monitored while receiving the infliximab infusion and for the period afterwards
- Feeling and being sick
- Headache
- Flu-like symptoms

During the days or weeks receiving Infliximab:

Very common side effects (experienced by 1 in 10 people) include:

- Increased risk of infection. Infliximab can interfere with the body's ability to fight other infections caused by bacteria, fungi, other viruses such as hepatitis B, and tuberculosis. You will be monitored

For peer review only - http://bmjopen.bmj.com/site/about/guidelines.xhtml

carefully for signs of infection other than COVID-19 and be offered appropriate treatment if another infection is detected.

Common side effects (experienced by between 1 in 10 to 1 in 100 people) include:

- Abnormal liver function tests. Blood tests will be done frequently to check that your liver is working properly.
- Some patients may experience worsening of psoriasis

Pregnancy and Breast Feeding

There is very little known about the effects of namilumab on an unborn baby and there is some information available about the infliximab on an unborn baby. As a precaution, women who are breast feeding are excluded from the trial and women of child bearing potential must have a negative pregnancy test prior to startng the trial. It is important that if you receive a trial treatment that you use adequate birth control if you (or your partner if you are male) are of child bearing potential. For namilumab, male and female participants should use effective contraception for 18 weeks after the last dose of drug. For infliximab, male and female participants should use contraception for 26 weeks after the last dose of drug.

Effective contraception is a method that has a failure rate of less than 1% a year when used correctly and all the time. Examples of these include:

- combined (oestrogen and progesterone containing) hormonal contraception e.g. the "pill", patch
- progesterone only contraception (includes the "mini-pill", injection, implant)
- Intrauterine device (IUD) or hormone-releasing system (IUS)
- vasectomised partner
- sexual abstinence

Recent guidelines in rheumatology patients suggests infliximab can be given to patients up until the 16th week of pregnancy. However, as this is the first time infliximab is being used in suspected or confirmed COVID-19 patients it is important that you understand the contraception information above.

It is also important that you do not breastfeed for 6 months after the last dose of namilumab or infliximab.

Is there any prohibited medication whilst I am on the trial?

Your trial doctor will look at the medicines that you are already taking and let you know whether you are able to still take them while you are taking part in the trial, or whether you would need to stop any of them.

For peer review only - http://bmjopen.bmj.com/site/about/guidelines.xhtml

What will happen if I don't want to carry on with the trial?

You can withdraw from the trial at any time without this having any effect on your medical care, however all information and blood samples already collected from your time on the trial will still be used.

Will my taking part in this trial be kept confidential?

All information collected about you for this trial will be subject to the General Data Protection Regulation 2018 and Data Protection Act 2018 for Health and Care Research and will be kept strictly confidential.

The University of Birmingham is the Sponsor for this trial. The University of Birmingham will be using information from your medical records in order to undertake this trial and will act as the data controller. This means that the University of Birmingham are responsible for looking after your information and using it properly. University of Birmingham and the NHS will keep identifiable information about you for at least 10 years after the trial has finished; this allows the results of the trial to be verified if needed.

In the Trial Office you will be identified by a unique trial number. In routine communication between your hospital and the Trial Office you will only be identified by trial number, initials and date of birth. Information about you, your health and wellbeing may be provided to the Trial Office on paper or electronically. We would also like to collect your NHS Number. This will allow researchers to collect information about your health and wellbeing from national records (e.g. Office for National Statistics, NHS Central Registries or other registries including those managed by NHS Digital) after the trial has ended. This will help us to determine the long-term impact of the trial treatment and COVID-19 on people's health.

By taking part in the trial you will be agreeing to allow research staff from the Trial Office to look at the trial records, including your medical records. It may be necessary to allow authorised personnel from government regulatory agencies (e.g. Medicines and Healthcare products Regulatory Agency (MHRA)), the Sponsor and/or NHS bodies to have access to information about you. This is to ensure that the trial is being conducted to the highest possible standards.

If you are randomised to receive namilumab or infliximab, pseudo-anonymised data from the trial may also be provided to the pharmaceutical company who is providing the drug you are given for safety monitoring or licensing purposes, where applicable. This is for your and others' protection to track the safety of the trial treatment. This may involve sending data outside of the United Kingdom to a European country of the United States of America. Your name and any identifying details (such as date of birth) will not be given to any of these parties.

We may be asked to share the trial information (data) we have collected with researchers running other studies, so that they can perform analysis on the data to answer other important questions about COVID-19.

For peer review only - http://bmjopen.bmj.com/site/about/guidelines.xhtml

These other researchers may be based in universities, NHS organisations, companies involved in health research and may be in this country or abroad. Non-identifiable summary information may also be shared with COVID-19 related UK government departments. Any such request is carefully considered by the trial researchers and will only be granted if the research is of high scientific standard and the necessary procedures and approvals are in place. The information will only be used for the purpose of health research and cannot be used to contact you or to affect your care. It will not be used to make decisions about future services available to you, such as insurance.

All individuals who have access to your information have a duty of confidentiality to you. Under no circumstances will you be identified in any way in any report, presentation or publication arising from this or any other research study.

You can withdraw your consent to our processing any more of your data at any time. Your rights to access change or move your information are limited, as we need to manage your information in specific ways in order for the research to be reliable and accurate. If you withdraw from the trial, we will keep the information about you and the samples that we have already obtained. Under the provisions of the General Data Protection Regulation 2018 and Data Protection Act 2018 you have the right to know what information the University of Birmingham has recorded about you. If you wish to view this information or find more about how we use this information, please contact Legal Services at Legal Services, University of Birmingham, Edgbaston, Birmingham, B15 2TT.

What will happen to the samples I give?

If you agree to take part in the optional research sample collection, the samples will be sent to laboratories based in the Universities of Birmingham or Oxford for storage and use in laboratory based research for the trial. If you give your consent, any samples leftover at the end of analysis for the trial, may be kept for future ethically approved research. These laboratory-based research projects will include genetic analysis of these samples. This DNA and RNA analysis is for scientific purposes and is not expected to provide findings of any clinical significance for you or your relatives, so the results will not be fed back to you. It is difficult to predict exactly what scientific developments there may be so we cannot give precise details of what research might be done.

No one using your samples for laboratory research will have access to your personal details. The samples sent to the University laboratories will be identified by your unique trial number only. All samples relating to you will be stored in accordance with the Human Tissue Act 2004.

For peer review only - http://bmjopen.bmj.com/site/about/guidelines.xhtml

What will happen to the results of the research trial?

At the end of the trial, the findings will be published in peer-reviewed medical and scientific journals. These publications will be available upon request from your trial doctor. We will also make a lay summary of the result available on the trial websites.

Who is organising and funding the research?

The trial is sponsored and being undertaken by the University of Birmingham in collaboration with University Hospitals Birmingham NHS Foundation Trust. The trial is being coordinated by the Cancer Research UK Clinical Trials Unit (CRCTU) within the University of Birmingham.

The trial is funded by an educational grant from UK Research and Innovation and drugs are being provided free of charge by the pharmaceutical companies.

Who has reviewed the trial?

All research in the NHS is looked at by an independent group of people called a Research Ethics Committee to protect your safety, rights, wellbeing and dignity. This trial has been reviewed and given favourable opinion by the East Midlands-Nottingham 2 Research Ethics Committee and by the NHS Health Research Authority. While the trial is ongoing the results will be reviewed regularly by an independent Data Monitoring Committee to ensure that it is appropriate to continue with the trial.

Expenses and Payments

As you will already be an inpatient in the hospital during the course of the trial you will not have to make any extra visits to participate in the trial and therefore will incur no additional expenses.

What if there is a problem?

If you have a concern about any aspect of this trial, you should ask to speak with your trial doctor who will do their best to answer your questions (see contact number at the end of this form). If you remain unhappy and wish to complain formally, you can do this through your hospital's Patient Advice and Liaison Services (PALS); they can be contacted by:

(Insert local contact details).

If you are harmed and this is due to someone's negligence then you may have grounds for legal action for compensation against the Sponsor of the trial (University of Birmingham) or the NHS Trust but you may have to pay your legal costs. NHS Trust Hospitals have a duty of care to all patients treated, whether or not the patient is taking part in a clinical trial, and the normal NHS complaints mechanisms will still be available to you (if appropriate).

For peer review only - http://bmjopen.bmj.com/site/about/guidelines.xhtml
For peer review only

Further information and contact details

Trial Doctor: ____

Research Nurse: ____

☎: _________________________ Emergency (24 hours) ☎: _________________________

For peer review only

For peer review only - http://bmjopen.bmj.com/site/about/guidelines.xhtml

<To be printed on hospital headed paper>

CATALYST - An early phase platform trial in (suspected or confirmed) COVID-19

Which treatment could lessen the severity of a (suspected or confirmed) COVID-19 infection when compared with usual care in an NHS setting?

Personal Legal Representative Information Sheet

Invitation to take part

CATALYST is a clinical trial being led by the University of Birmingham and University Hospitals Birmingham NHS Foundation Trust in collaboration with the University of Oxford. You are being asked to assess the information contained within this information sheet on the patient's behalf, and make a decision based on the presumed wish of the patient on whether they would like to take part in the trial or not. The decision must be based on the patient's presumed will, and not based on your own views or objection.

Taking part is entirely voluntary, and choosing not to take part in this trial will not affect his/ her care in any way.

Before you decide whether the patient would want to take part, it is essential for you to understand why the research is being done and what his/her participation will involve. For this reason, the information contained within this personal legal representative information sheet is identical to that contained within the patient information sheet. The patient information sheet will be presented to the patient should he/ she clinically improve to the point of being able to assess the information to make an informed decision on whether to continue to take part in the trial or not.

Please take time to read the following information carefully.

The purpose of the trial

There is currently no vaccine and few effective treatments for COVID-19. As COVID-19 is a new illness, we are continually learning more about how it affects the human body. We know that the COVID-19 virus affects a number of different cells in the body, including a type of blood cell called a macrophage (immune cell), and that it can cause the number of these cells to increase in the body. To fight an infection, immune cells produce proteins called cytokines and chemokines. These proteins can cause inflammation and at high levels can lead to

For peer review only - http://bmjopen.bmj.com/site/about/guidelines.xhtml

damage in the tissues and organs in the body. Researchers believe this is why some people with COVID-19 infection become very ill.

Therapies we are testing:

A new, unlicensed drug called namilumab which has been tested in patients with arthritis and other inflammatory conditions. It may reduce inflammation in the body caused by the coronavirus. It is currently being given to patients with COVID-19 in a clinical trial in Italy. Namilumab is being provided free of charge by Izana Bioscience Limited (now part of Roivant Sciences) for use in this trial.

A drug called infliximab (Remsima) which is widely used to treat arthritis and other conditions may reduce inflammation in the body caused by the coronavirus. Infliximab is being provided free of charge by Celltrion Healthcare UK Limited for use in this trial.

Once this inflammation has been reduced, it may be possible that the immune system will adapt and fight off the virus more effectively.

Infliximab has not been tested in patients with COVID-19, and nalimumab has only been given to a limited number of patients in Italy. Therefore, the purpose of this trial is to assess if these drugs may be helpful in treating COVID-19. If in this trial any drug shows an initial sign of benefit in (suspected or confirmed) COVID-19, then it will be included in another larger-scale clinical trial being conducted throughout the UK, which is designed to compare treatments to find out which is the best at treating this infection.

This is a national trial and some hospitals running this trial are unable to offer patients all of the therapies available within this trial. The doctor will tell you which treatments are available at the hospital, and if applicable, which ones are not available.

Why has my relative/ friend been invited?

Your relative/ friend has been invited to take part in this trial because they are aged 16 years or older, they have been admitted to hospital, and their doctor believes they have either a suspected or confirmed COVID -19 infection or have tested positive with a COVID-19 infection and they have higher levels of C-reactive protein in their blood than normal (this increases when there's an infection or inflammation in your body).

What will happen to my relative/ friend if they take part?

For peer review only - http://bmjopen.bmj.com/site/about/guidelines.xhtml
For peer review only

- You will agree for your relative/ friend to take part in a clinical trial, and they will be actively monitored for 28 days and longer if necessary.
- This clinical trial has three treatment groups or 'arms': one for each of the treatments listed above, and the other arm is 'usual care'. Usual care means that they will receive the same treatment that everyone with a (suspected or confirmed) COVID-19 infection would get in the hospital whether you decide your relative/ friend takes part in the trial or not.
- The treatment they will receive is randomly allocated (chosen by chance) by a computer. Neither you nor your relative's/ friend's doctor will be able to select the treatment your relative/ friend receives. The research doctor or nurse will inform you of which treatment your relative/ friend will receive once they have entered them into the trial.
- If your relative/ friend is allocated the 'usual care' arm, they will receive the same medical care as all other patients being treated for a (suspected or confirmed) COVID-19 infection.
- If your relative/ friend is allocated to receive namilumab, they will get this in addition to the usual medical care received by patients who have this disease. Namilumab will be given to your relative/ friend through a drip in a vein, usually in their arm, on one occasion only.
- If your relative/ friend is allocated to receive infliximab, they will get this in addition to the usual medical care received by patients who have this disease. Infliximab will be given to your relative/ friend through a drip in a vein, usually in their arm, on one occasion only.
- Your relative/ friend will have routine blood samples while they are in the hospital as part of 'usual care.' If your relative/ friend is allocated to the namilumab or infliximab arm, they may have more blood samples taken regularly before and during treatment to check it is safe for them to receive treatment.
- If your relative/ friend are a female of childbearing potential, a pregnancy test will be performed as part of the screening tests.
- We would also like your permission to collect samples from your relative/ friend for research. The collection of these samples is optional. **This will only take place at certain hospitals**. The doctor will tell you if research samples are being collected at your relative/ friend's hospital. This includes collecting samples of blood, and may include swabs from your relative/ friend's nose and throat, and if they are placed on a ventilator, samples of the secretions from the tube placed in their windpipe to help them breathe may be taken. Samples will be collected on up to three separate occasions. These samples will be used in laboratory studies to gain a better understanding of COVID-19 and how patients respond to treatment.
- While your relative/ friend is in hospital, their health and wellbeing will be monitored in accordance with usual care.

For peer review only - http://bmjopen.bmj.com/site/about/guidelines.xhtml

What are the potential benefits of taking part?

This research has been designed to help develop a treatment for future patients with (suspected or confirmed) COVID-19 infection. It is important to understand that your relative/ friend may not directly benefit from taking part in this trial. The benefits for them as an individual are unknown.

What are the possible disadvantages and risks of taking part?

Your relative/ friend may have side effects from the treatment while taking part in the trial. This trial is testing a new way of treating (suspected or confirmed) COVID-19 infection.

All of the treatments have been given to humans before but not in (suspected or confirmed) COVID-19 infection, so there may be side effects that the doctors are not currently aware of, and these may be serious; because of this an independent committee will be monitoring the safety of the treatments in this trial regularly.

Everyone taking part in the trial will be monitored carefully for side effects. However, the doctors do not know all the side effects that may occur and we don't know how the drugs used in this trial will interact with the other drugs being used to treat (suspected or confirmed) COVID-19. Side effects may be mild or serious or may even be life-threatening. The doctors may give your relative/ friend medicines to help lessen side effects or the trial treatment may be postponed or stopped, depending on the side-effects they may experience.

Specific information is provided below for each of the trial treatments available in the trial is included below:

Namilumab

Namilumab is a new drug that is being tested to treat diseases that cause inflammation such as rheumatoid arthritis.

The side effects that are known so far are:

- Low neutrophil count (a type of white blood cell)
- Minor symptoms such as runny nose and headache
- Tachycardia (a fast heart beat)*

* A short change in heart rhythm was seen with no apparent harm in one person receiving namilumab in another trial.

Infliximab

Infliximab has been widely used for rheumatoid arthritis and other conditions for 20 years but it has some potential side effects. It has been used to treat patients with sepsis (serious infection) before on intensive care units and was demonstrated to be safe. The more common known side effects of infliximab when used to treat other conditions such as arthritis have been summarised below:

For peer review only - http://bmjopen.bmj.com/site/about/guidelines.xhtml

For peer review only

While your relative/ friend is receiving Infliximab:

- Common side effects (experienced by 1 in 100 people) include:
- Allergic reaction: this can be mild but is sometimes severe and may even be life-threatening. For this reason your relative/ friend will be very carefully monitored while receiving the iInfliximab infusion and for the period afterwards
- Feeling and being sick
- Headache
- Flu-like symptoms

During the days or weeks receiving Infliximab:

Very common side effects (experienced by 1 in 10 people) include:

- Increased risk of infection. Infliximab can interfere with the body's ability to fight other infections caused by bacteria, fungi, other viruses such as hepatitis B, and tuberculosis. Your relative/ friend will be monitored carefully for signs of infection other than COVID-19 and be offered appropriate treatment if another infection is detected.

Common side effects (experienced by between 1 in 10 to 1 in 100 people) include:

- Abnormal liver function tests. Blood tests will be done frequently to check that your relative/ friend's liver is working properly.
- Some patients may experience worsening of psoriasis

Pregnancy and Breast Feeding

There is very little known about the effects of namilumab on an unborn baby and there is some information available about the and infliximab on an unborn baby,. As a precaution, women who are breast feeding are excluded from the trial and women of child bearing potential must have a negative pregnancy test prior to startng the trial. It is important that if your relative/ friend receives a trial treatment that they use adequate birth control if they (or their partner if they are male) are of child bearing potential. For namilumab, male and female participants should use effective contraception for 18 weeks after the last dose of drug. For infliximab, male and female participants should use contraception for 26 weeks after the last dose of drug.

Effective contraception is a method that has a failure rate of less than 1% a year when used correctly and all the time. Examples of these include:

- combined (oestrogen and progesterone containing) hormonal contraception e.g. the "pill", patch
- progesterone only contraception (includes the "mini-pill", injection, implant)
- Intrauterine device (IUD) or hormone-releasing system (IUS)
- vasectomised partner

For peer review only - http://bmjopen.bmj.com/site/about/guidelines.xhtml

- sexual abstinence

Recent guidelines in rheumatology patients suggests infliximab can be given to female patients up until the 16th week of pregnancy. However, as this is the first time infliximab is being used in (suspected or confirmed) COVID-19 patients it is important your relative/ friend understands the contraception information above.
It is also important that any female patients do not breastfeed for 6 months after the last dose of namilumab or infliximab.

Is there any prohibited medication whilst my relative/ friend is on the trial?

Your relative/ friend's trial doctor will look at the medicines that they are already taking and let you/ your relative/ friend know whether they are able to still take them while they are taking part in the trial, or whether they would need to stop any of them.

What will happen if I don't want my relative/ friend to carry on with the trial?

You can withdraw your relative/ friend from the trial at any time without this having any effect on their medical care, however, all information and blood samples already collected from their time on the trial will still be used.

Will their taking part in this trial be kept confidential?

All information collected about your relative/ friend for this trial will be subject to the General Data Protection Regulation 2018 and Data Protection Act 2018 for Health and Care Research and will be kept strictly confidential.

The University of Birmingham is the Sponsor for this trial. The University of Birmingham will be using information from your relative/ friend's medical records in order to undertake this trial and will act as the data controller. This means that the University of Birmingham are responsible for looking after your relative/ friend's information and using it properly. The University of Birmingham and the NHS will keep identifiable information about them for at least ten years after the trial has finished; this allows the results of the trial to be verified if needed.

In the Trial Office, your relative/ friend will be identified by a unique trial number. In routine communication between the hospital and the Trial Office, your relative/ friend will only be identified by trial number, initials and date of birth. Information about your relative/ friend, their health and wellbeing may be provided to the Trial Office on paper or electronically. We would also like to collect your relative/ friend's NHS Number. This will allow researchers to collect information about their health and wellbeing from national records (e.g. Office for National Statistics, NHS Central Registries or other registries including those managed by NHS Digital) after the

For peer review only - http://bmjopen.bmj.com/site/about/guidelines.xhtml

trial has ended. This will help us to determine the long-term impact of the trial treatment and COVID-19 on people's health.

By taking part in the trial, you will agree to allow research staff from the Trial Office to look at the trial records, including the medical records of your relative/ friend. It may be necessary to allow authorised personnel from government regulatory agencies (e.g. Medicines and Healthcare products Regulatory Agency (MHRA)), the Sponsor and/or NHS bodies to have access to information about your relative/ friend. This is to ensure that the trial is being conducted to the highest possible standards.

If the your relative/ friend is randomised to receive namilumab or infliximab, pseudo-anonymised data from the trial may also be provided to the pharmaceutical company who is providing the drug they are given for safety monitoring or licensing purposes, where applicable. This is for their and others' protection to track the safety of the trial treatment. This may involve sending data outside of the United Kingdom to a European country of the United States of America. Your relative/ friend's name and any identifying details (such as date of birth) will not be given to any of these parties.

We may be asked to share the trial information (data) we have collected with researchers running other studies so that they can perform analysis on the data to answer other important questions about COVID-19. These other researchers may be based in universities, NHS organisations companies involved in health research and maybe in this country or abroad. Non-identifiable summary information may also be shared with COVID-19 related UK government departments. Any such request is carefully considered by the trial researchers and will only be granted if the research is of high scientific standard and the necessary procedures and approvals are in place. The information will only be used for the purpose of health research and cannot be used to contact your relative/ friend or affect their care. It will not be used to make decisions about future services available to them, such as insurance.

All individuals who have access to your relative/ friend's information have a duty of confidentiality to them. Under no circumstances will they be identified in any way in any report, presentation or publication arising from this or any other research study.

You can withdraw your consent to our processing any more of your relative/ friend's data at any time. Your rights to access change or move your relative/ friend's information are limited, as we need to manage their information in specific ways in order for the research to be reliable and accurate. If you withdraw your relative/ friend from the trial, we will keep the information about them and the samples that we have already obtained. Under the provisions of the General Data Protection Regulation 2018 and Data Protection Act 2018, your relative/ friend has the right to know what information the University of Birmingham has recorded about them.

For peer review only - http://bmjopen.bmj.com/site/about/guidelines.xhtml

If your relative/ friend wishes to view this information or find more about how we use this information, please contact Legal Services at Legal Services, University of Birmingham, Edgbaston, Birmingham, B15 2TT.

What will happen to the samples they give?

If you agree for your relative/ friend to take part in the optional research sample collection, the samples will be sent to laboratories based in the Universities of Birmingham or Oxford for storage and use in laboratory-based research for the trial. If you give your consent, any samples leftover at the end of analysis for the trial, may be kept for future ethically approved research. This laboratory-based research will include genetic analysis of these samples. This DNA and RNA analysis is for scientific purposes and is not expected to provide findings of any clinical significance for your relative/ friend or their relatives, so the results will not be fed back to your relative/ friend. It is difficult to predict precisely what scientific developments there may be so we cannot give precise details of what research might be done.

No one using your relative/ friend's samples for laboratory research will have access to their personal details. The samples sent to the University laboratories will be identified by your relative/ friend's unique trial number only. All samples relating to them will be stored in accordance with the Human Tissue Act 2004.

For peer review only

What will happen to the results of the research trial?

At the end of the trial, the findings will be published in in peer-reviewed medical and scientific journals. These publications will be available upon request from your relative/ friend's trial doctor. We will also make a lay summary of the result available on the trial websites.

Who is organising and funding the research?

The trial is sponsored and being undertaken by the University of Birmingham in collaboration with University Hospitals Birmingham NHS Foundation Trust . The trial is being coordinated by the Cancer Research UK Clinical Trials Unit (CRCTU) within the University of Birmingham.

The trial is funded by an educational grant from UK Research and Innovation and drugs are being provided free of charge by the pharmaceutical companies.

Who has reviewed the trial?

All research in the NHS is looked at by an independent group of people called a Research Ethics Committee to protect your relative/ friend's safety, rights, wellbeing and dignity. This trial has been reviewed and given favourable opinion by the East Midlands-Nottingham 2 Research Ethics Committee and by the NHS Health Research Authority. While the trial is ongoing the results will be reviewed regularly by an independent Data Monitoring Committee to ensure that it is appropriate to continue with the trial.

For peer review only - http://bmjopen.bmj.com/site/about/guidelines.xhtml

Expenses and Payments

As your relative/ friend will already be an inpatient in the hospital during the trial they will not have to make any extra visits to participate in the trial and therefore will incur no additional expenses.

What if there is a problem?

If you have a concern about any aspect of this trial, you should ask to speak with your relative/ friend's trial doctor who will do their best to answer your questions (see contact number at the end of this form). If you remain unhappy and wish to complain formally, you can do this through your relative/ friend's hospital's Patient Advice and Liaison Services (PALS); they can be contacted by:

(Insert local contact details).

If your relative/ friend is harmed and this is due to someone's negligence then your relative/ friend may have grounds for legal action for compensation against the Sponsor of the trial (University of Birmingham) or the NHS Trust but they may have to pay their legal costs. NHS Trust Hospitals have a duty of care to all patients treated, whether or not the patient is taking part in a clinical trial, and the normal NHS complaints mechanisms will still be available to your relative/ friend (if appropriate).

Further information and contact details

Trial Doctor: ____

Research Nurse: ____

☎: ______________________ Emergency (24 hours) ☎: ______________________

For peer review only - http://bmjopen.bmj.com/site/about/guidelines.xhtml

<To be printed on hospital headed paper>

CATALYST - An early phase platform trial in (suspected or confirmed) COVID-19

Which treatment could lessen the severity of a (suspected or confirmed) COVID-19 infection when compared with usual care in an NHS setting?

Professional Legal Representative Information Sheet

For peer review only

Invitation to take part

CATALYST is a clinical trial being led by the University of Birmingham and University Hospitals Birmingham NHS Foundation Trustand in collaboration with the University of Oxford. You are being asked to consent on the basis of the presumed will of the patient by assessing the information contained within this information sheet. This applies both to the wish of the patient to take part, or to refuse to take part. Taking part is entirely voluntary, and choosing not to take part in this trial will not affect his/ her care in any way.

Before you decide whether the patient would want to take part, it is essential for you to understand why the research is being done and what his/her participation will involve. For this reason, the information contained within this professional legal information sheet is identical to that contained within the patient information sheet. The patient information sheet will be presented to the patient should he/ she clinically improve to the point of being able to assess the information in order to make an informed decision on whether to continue to take part in the trial or not.

Please take time to read the following information carefully.

The purpose of the trial

There is currently no vaccine and few effective treatments for COVID-19. As COVID-19 is a new illness, we are constantly learning more about how it affects the human body. We know that the COVID-19 virus affects several different cells in the body, including a type of blood cell called a macrophage (immune cell), and that it can cause the number of these cells to increase in the body. To fight an infection, immune cells produce proteins called cytokines and chemokines. These proteins can cause inflammation and at high levels can lead to damage in the tissues and organs in the body. Researchers believe this is why some people with COVID-19 infection become very ill.

For peer review only - http://bmjopen.bmj.com/site/about/guidelines.xhtml

Therapies we are testing:

A new, unlicensed drug called namilumab which has been tested in patients with arthritis and other inflammatory conditions. It may reduce inflammation in the body caused by the coronavirus. It is currently being given to patients with COVID-19 in a clinical trial in Italy. Namilumab is being provided free of charge by Izana Bioscience Limited (now part of Roivant Sciences) for use in this trial.

A drug called Infliximab (Remsima) which is widely used to treat arthritis and other conditions may reduce inflammation in the body caused by the coronavirus. Infliximab is being provided free of charge by Celltrion Healthcare UK Limited for use in this trial.

Once this inflammation has been reduced, it may be possible that the immune system will adapt and fight off the virus more effectively.

Infliximab has not been tested in patients with COVID-19, and namilumab has only been given to a limited number of patients in Italy. Therefore, the purpose of this trial is to assess if these drugs may be helpful in treating COVID-19. If any of the drugs benefit people with a (suspected or confirmed) COVID-19 infection in this trial, the drug will be included in another larger-scale clinical trial being conducted throughout the UK, which is designed to compare treatments to find out which is the best at treating this infection.

This is a national trial, and some hospitals running this trial are unable to offer patients all of the therapies available within this trial. The trial doctor will tell you which treatments are available at your hospital, and if applicable, which ones are not available.

Why has the patient been invited?

The patient has been invited to take part in this trial because they are aged 16 years or older, they have been admitted to hospital, and their doctor believes they have either a suspected or confirmed COVID -19 infection and the they have higher levels of C-reactive protein in their blood than normal (this increases when there's an infection or inflammation in your body).

What will happen to the patient if they take part?

- You will agree for the patient to take part in a clinical trial and they will be actively monitored for 28 days and longer if necessary.

For peer review only - http://bmjopen.bmj.com/site/about/guidelines.xhtml

- This clinical trial has three treatment groups or 'arms': one for each of the treatments listed above, and the other arm is 'usual care'. Usual care means that the patient will receive exactly the same treatment that everyone with a (suspected or confirmed) COVID-19 infection would receive in the hospital whether you decide the patient takes part in the trial or not. The treatment the patient will receive is randomly allocated (chosen by chance) by a computer. Neither you nor the patient's doctor will be able to choose the treatment they receive. The research doctor or nurse will inform you of which treatment the patient will receive once they have entered the patient into the trial.
- If the patient is allocated the 'usual care' arm, they will receive the same medical care as all other patients being treated for a (suspected or confirmed) COVID-19 infection.
- If the patient is allocated to receive namilumab, they will receive this in addition to the usual medical care received by patients who have this disease. Namilumab will be given to the patient through a drip in a vein, usually in their arm, on one occasion only.
- If the patient is allocated to receive infliximab, they will receive this in addition to the usual medical care received by patients who have this disease. Infliximab will be given to the patient through a drip in a vein, usually in their arm, on one occasion only.
- The patient will have routine blood samples whilst they are in the hospital as part of 'usual care.' If the patient is allocated to the namilumab or infliximab arm, the patient may have more blood samples taken regularly before and during treatment to check it is safe for them to receive treatment.
- If the patient is a female of childbearing potential, a pregnancy test will be performed as part of the screening tests.
- We would also like your permission to collect samples for research from the patient. The collection of these samples is optional. **This will only take place at certain hospitals.** The doctor will tell you if research samples are being collected at the patient's hospital. This includes collecting samples of blood, and may include swabs from their nose and throat, and if they are placed on a ventilator, samples of the secretions from the tube placed in their windpipe to help them breathe may be taken. Samples will be collected on up to three separate occasions. These samples will be used in laboratory studies to gain a better understanding of COVID-19 and how patients respond to treatment.
- While the patient is in hospital their health and wellbeing will be monitored in accordance with usual care

What are the potential benefits of taking part?

This research has been designed to help develop a treatment for future patients with (suspected or confirmed) COVID-19 infection. It is important to understand that the patient may not directly benefit from taking part in this trial. The benefits for the patient as an individual are unknown.

For peer review only - http://bmjopen.bmj.com/site/about/guidelines.xhtml

What are the possible disadvantages and risks of taking part?

This trial is testing a new way of treating (suspected or confirmed) COVID-19 infection and the patient may have side effects from the treatment while taking part in the trial.

All of the treatments have been given to humans before but not in (suspected or confirmed) COVID-19 infection so there may be side effects that the doctors are not currently aware of, and these may be serious; because of this an independent committee will be monitoring the safety of the treatments in this trial on a regular basis.

Everyone taking part in the trial will be monitored carefully for side effects. However, the doctors do not know all the side effects that may occur and we don't know how the drugs used in this trial will interact with the other drugs being used to treat (suspected or confirmed) COVID-19. Side effects may be mild or serious or may even be life-threatening. The doctors may give the patient medicines to help lessen side effects or the trial treatment may be postponed or stopped, depending on the side-effects they may experience.

Specific information is provided below for each of the trial treatments available in the trial is included below:

Namilumab

Namilumab is a new drug that is being tested to treat diseases that cause inflammation such as rheumatoid arthritis.

The side effects that are known so far are:

- Low neutrophil count (a type of white blood cell)
- Minor symptoms such as runny nose and headache
- Tachycardia (a fast heart beat)*

* A short change in heart rhythm was seen with no apparent harm in one person receiving namilumab in another trial.

Infliximab

Infliximab has been widely used for rheumatoid arthritis and other conditions for 20 years but it has some potential side effects. It has been used to treat patients with sepsis (serious infection) before on intensive care units and was demonstrated to be safe. The more common known side effects of infliximab when used to treat other conditions such as arthritis have been summarised below:

Whilst receiving Infliximab:

- Common side effects (experienced by 1 in 100 people) include:

For peer review only - http://bmjopen.bmj.com/site/about/guidelines.xhtml

- Allergic reaction: this can be mild but is sometimes severe and may even be life-threatening. For this reason the patient will be very carefully monitored while receiving the iInfliximab infusion and for the period afterwards
- Feeling and being sick
- Headache
- Flu-like symptoms

During the days or weeks receiving Infliximab:

Very common side effects (experienced by 1 in 10 people) include:

- Increased risk of infection. Infliximab can interfere with the body's ability to fight other infections caused by bacteria, fungi, other viruses such as hepatitis B, and tuberculosis. The patient will be monitored carefully for signs of infection other than COVID-19 and be offered appropriate treatment if another infection is detected.

Common side effects (experienced by between 1 in 10 to 1 in 100 people) include:

- Abnormal liver function tests. Blood tests will be done frequently to check that the patient's liver is working properly.
- Some patients may experience worsening of psoriasis

Pregnancy and Breast Feeding

There is very little known about the effects of namilumab on an unborn baby and there is some information available about the and infliximab on an unborn baby. As a precaution, women who are breast feeding are excluded from the trial and women of child bearing potential must have a negative pregnancy test prior to startng the trial. It is important that if the patient receive's a trial treatment that they use adequate birth control if they (or their partner if they are male) are of child bearing potential. For namilumab, male and female participants should use effective contraception for 18 weeks after the last dose of drug. For infliximab, male and female participants should use contraception for 26 weeks after the last dose of drug.

Effective contraception is a method that has a failure rate of less than 1% a year when used correctly and all the time. Examples of these include:

- combined (oestrogen and progesterone containing) hormonal contraception e.g. the "pill", patch
- progesterone only contraception (includes the "mini-pill", injection, implant)
- Intrauterine device (IUD) or hormone-releasing system (IUS)
- vasectomised partner
- sexual abstinence

For peer review only - http://bmjopen.bmj.com/site/about/guidelines.xhtml

Recent guidelines in rheumatology patients suggests infliximab can be given to patients up until the 16th week of pregnancy. However, as this is the first time infliximab is being used in (suspected or confirmed) COVID-19 patients it is important that the patient understands the contracpetion information above.

It is also important that any female patients do not breastfeed for 6 months after the last dose of namilumab or infliximab.

Is there any prohibited medication whilst the patient is on the trial?

The trial doctor will look at the medicines that the patient is already taking and let you know whether the patient is able to still take them while they are taking part in the trial, or whether they would need to stop any of them.

What will happen if I don't want the patient to carry on with the trial?

You can withdraw the patient from the trial at any time without this having any effect on their medical care, however all information and blood samples already collected from the patient's time on the trial will still be used.

Will their taking part in this trial be kept confidential?

All information collected about the patient for this trial will be subject to the General Data Protection Regulation 2018 and Data Protection Act 2018 for Health and Care Research and will be kept strictly confidential.

The University of Birmingham is the Sponsor for this trial. The University of Birmingham will be using information from the patient's medical records in order to undertake this trial and will act as the data controller. This means that the University of Birmingham are responsible for looking after the patient's information and using it properly. The University of Birmingham and the NHS will keep identifiable information about the patient for at least 10 years after the trial has finished; this allows the results of the trial to be verified if needed.

In the Trial Office the patient will be identified by a unique trial number. In routine communication between their hospital and the Trial Office the patient will only be identified by trial number, initials and date of birth. Information about the patient, their health and wellbeing may be provided to the Trial Office on paper or electronically. We would also like to collect the patient's NHS Number. This will allow researchers to collect information about their health and wellbeing from national records (e.g. Office for National Statistics, NHS Central Registries or other registries including those managed by NHS Digital) after the trial has ended. This will help us to determine the long-term impact of the trial treatment and COVID-19 on people's health.

For peer review only - http://bmjopen.bmj.com/site/about/guidelines.xhtml

By taking part in the trial, you will be agreeing to allow research staff from the Trial Office to look at the trial records, including the patient's medical records. It may be necessary to allow authorised personnel from government regulatory agencies (e.g. Medicines and Healthcare products Regulatory Agency (MHRA)), the Sponsor and/or NHS bodies to have access to information about the patient. This is to ensure that the trial is being conducted to the highest possible standards.

If the patient is randomised to receive namilumab or infliximab, pseudo-anonymised data from the trial may also be provided to the pharmaceutical company who is providing the drug the patient is given for safety monitoring or licensing purposes, where applicable. This is for the patient's and others' protection to track the safety of the trial treatment. This may involve sending data outside of the United Kingdom to a European country of the United States of America. The patient's name and any identifying details (such as date of birth) will not be given to any of these parties.

We may be asked to share the trial information (data) we have collected with researchers running other studies, so that they can perform analysis on the data to answer other important questions about COVID-19. These other researchers may be based in universities, NHS organisations, companies involved in health research and may be in this country or abroad. Non-identifiable summary information may also be shared with COVID-19 related UK government departments. Any such request is carefully considered by the trial researchers and will only be granted if the research is of high scientific standard and the necessary procedures and approvals are in place. The information will only be used for the purpose of health research and cannot be used to contact you, the patient, or to affect the patient's care. It will not be used to make decisions about future services available to them, such as insurance.

All individuals who have access to the patient's information have a duty of confidentiality to them. Under no circumstances will the patient be identified in any way in any report, presentation or publication arising from this or any other research study.

You can withdraw your consent to our processing any more of the patient's data at any time. Your rights to access change or move the patient's information are limited, as we need to manage the information in specific ways in order for the research to be reliable and accurate. If you withdraw the patient from the trial, we will keep the information about them and the samples that we have already obtained. Under the provisions of the General Data Protection Regulation 2018 and Data Protection Act 2018 the patient has the right to know what information the University of Birmingham has recorded about the patient. If the patient wishes to view this information or find more about how we use this information, they should contact Legal Services at Legal Services, University of Birmingham, Edgbaston, Birmingham, B15 2TT.

For peer review only - http://bmjopen.bmj.com/site/about/guidelines.xhtml

What will happen to the samples the patient gives?

If you agree for the patient to take part in the optional research sample collection, the samples will be sent to laboratories based in the Universities of Birmingham or Oxford for storage and use in laboratory-based research for the trial. If you give your consent, any samples leftover at the end of analysis for the trial, may be kept for future ethically approved research. These laboratory-based research projects will include genetic analysis of these samples. This DNA and RNA analysis is for scientific purposes and is not expected to provide findings of any clinical significance for the patient or their relatives, so the results will not be fed back to the patient. It is difficult to predict precisely what scientific developments there may be so we cannot give precise details of what research might be done.

No one using the samples for laboratory research will have access to the patient's personal details. The samples sent to the University laboratories will be identified by the patient's unique trial number only. All samples relating to the patient will be stored in accordance with the Human Tissue Act 2004.

What will happen to the results of the research trial?

At the end of the trial, the findings will be published in peer-reviewed medical and scientific journals. These publications will be available to the patient upon request from their trial doctor. We will also make a lay summary of the result available on the trial websites.

Who is organising and funding the research?

The trial is sponsored and being undertaken by the University of Birmingham in collaboration with University Hospitals Birmingham NHS Foundation Trust The trial is being coordinated by the Cancer Research UK Clinical Trials Unit (CRCTU) within the University of Birmingham.

The trial is funded by an educational grant from UK Research and Innovation and drugs are being provided free of charge by the pharmaceutical companies.

Who has reviewed the trial?

All research in the NHS is looked at by an independent group of people called a Research Ethics Committee to protect the patient's safety, rights, wellbeing and dignity. This trial has been reviewed and given favourable opinion by the East Midlands-Nottingham 2 Research Ethics Committee and by the NHS Health Research Authority. While the trial is ongoing the results will be reviewed regularly by an independent Data Monitoring Committee to ensure that it is appropriate to continue with the trial.

For peer review only - http://bmjopen.bmj.com/site/about/guidelines.xhtml

Expenses and Payments

As the patient will already be an inpatient in the hospital during the course of the trial they will not have to make any extra visits to participate in the trial and therefore will incur no additional expenses.

What if there is a problem?

If you have a concern about any aspect of this trial, you should ask to speak with the patient's trial doctor who will do their best to answer your questions (see contact number at the end of this form). If you remain unhappy and wish to complain formally, you can do this through the hospital's Patient Advice and Liaison Services (PALS); they can be contacted by:

(Insert local contact details).

If the patient is harmed and this is due to someone's negligence then there may be grounds for legal action for compensation against the Sponsor of the trial (University of Birmingham) or the NHS Trust. NHS Trust Hospitals have a duty of care to all patients treated, whether or not the patient is taking part in a clinical trial, and the normal NHS complaints mechanisms will still be available to them (if appropriate).

Further information and contact details

Trial Doctor: ____

Research Nurse: ____

☎: ______________________ Emergency (24 hours) ☎: ______________________

For peer review only

For peer review only - http://bmjopen.bmj.com/site/about/guidelines.xhtml

Supplementary Appendix 5 - Informed Consent Forms

<To be printed on hospital headed paper>

CATALYST- Early phase platform trial in (suspected or confirmed) COVID-19

Which treatment could lessen the severity of a (suspected or confirmed) COVID-19 infection when compared with usual care in an NHS setting?

Informed Consent Form

Site: .... **Patient's Trial Number:** [ ][ ][ ]

Investigator: .... **EudraCT:** 2020-001684-89

Please initial each box

1. I confirm that I have read and understood the Patient Information Sheet (version .... dated.........................) for the above trial. I have had the opportunity to consider the information, ask questions and have had these answered satisfactorily. [ ]

2. I understand that my participation is voluntary and that I am free to withdraw at any time without giving any reason, without my medical care or legal rights being affected. [ ]

3. I give permission for my initials, date of birth and NHS Number to be given to the Trial Office based within the University of Birmingham (Birmingham, UK) when I am randomised into the trial. [ ]

Original to be kept in the Investigator Site File, 1 copy in hospital notes, 1 copy to the patient

For peer review only - http://bmjopen.bmj.com/site/about/guidelines.xhtml

4. I understand that relevant sections of my medical notes and data collected during the trial may be looked at by individuals from the Trial Office, regulatory authorities, the Sponsor (the University of Birmingham) and/or NHS bodies, where it is relevant to my taking part in this research. I give permission for these individuals to have access to my records. ☐

5. I understand that data and samples from the trial may be provided to third parties (e.g. laboratories, pharmaceutical companies, other academic institutions or relevant UK government departments) for research, safety monitoring or licensing purposes, where applicable. I understand that this may involve sending data outside of the United Kingdom to a European country or the United States of America and that my name and any identifying details will NOT be given to these third parties, instead I will be identified by my unique Trial Number. ☐

6. I understand that if I withdraw from the trial, any data collected up until the date of my withdrawal will be analysed and used as part of the research. ☐

7. I agree to my GP being informed of my participation in this trial. ☐

8. I agree to take part in the above trial. ☐

OPTIONAL

9. I agree to the collection and storage of research samples for analysis as part of the CATALYST trial and for use in other ethically approved laboratory projects, which may include genetic analysis of these samples. I understand that I will not be personally informed of the results of these additional research projects and that if I withdraw from the trial any samples that have been collected up to the date of my withdrawal will not be destroyed. ☐

For peer review only - http://bmjopen.bmj.com/site/about/guidelines.xhtml

Name of patient	Date	Signature
Name of person taking consent	Date	Signature

You must have signed the Site Signature & Delegation Log

Original to be kept in the Investigator Site File, 1 copy in hospital notes, 1 copy to the patient

For peer review only - http://bmjopen.bmj.com/site/about/guidelines.xhtml

<To be printed on hospital headed paper>

CATALYST- Early phase platform trial in (suspected or confirmed) COVID 19

Which treatment could lessen the severity of a (suspected or confirmed) COVID 19 infection when compared with usual care in an NHS setting?

Personal Representative Informed Consent Form

Site: .... **Patient's Trial Number:** ☐☐☐

Investigator: .... **EudraCT:** 2020-001684-89

Please initial each box

1. I confirm that I been consulted about ....'s participant in this trial and have read and understand the Personal Legal Representative Information Sheet (version .... dated........................) for the above trial and what it means to be a personal legal representative. I have had the opportunity to consider the information, ask questions and have had these answered satisfactorily. ☐

2. I understand that his/her participation is voluntary and that I am free to change my opinion on what he/she would have wished for at any time without giving any reason, and without his/her medical care or legal rights being affected. ☐

3. I understand that his/her direct informed consent will be obtained at the earliest opportunity. ☐

Original to be kept in the Investigator Site File, 1 copy in hospital notes, 1 copy to the patient/ legal representative

For peer review only - http://bmjopen.bmj.com/site/about/guidelines.xhtml

4. I understand that his/her initials, date of birth and NHS Number will be given to the Trial Office based within the University of Birmingham (Birmingham, UK) when he/she is randomised to the trial.

5. I understand that relevant sections of his/her medical notes and data collected during the trial may be looked at by individuals from the Trial Office, regulatory authorities, the Sponsor (the University of Birmingham) and/or NHS bodies, where it is relevant to him/her taking part in this research.

6. I understand that data and samples from the trial may be provided to third parties (e.g. laboratories, pharmaceutical companies, other academic institutions or relevant UK government departments) for research, safety monitoring or licensing purposes, where applicable. I understand that this may involve sending data outside of the United Kingdom to a European country or the United States of America and that the patients name and any identifying details will NOT be given to these 3rd parties, instead he/she will be identified by his/ her unique Trial Number.

7. I understand that if he/she withdraws or is withdrawn from the trial, any samples and data that have been collected up to the date of withdrawal will be analysed and used as part of the research.

8. I agree to the patients GP being informed of his/ her participation in this trial.

9. In my opinion, the patient would have no objections to taking part in the above trial

OPTIONAL

10. I agree to the collection and storage of research samples for analysis as part of the CATALYST trial and for use in other ethically approved laboratory projects, which may include genetic analysis of these

For peer review only - http://bmjopen.bmj.com/site/about/guidelines.xhtml

samples. I understand that the patient will not be personally informed of the results of these additional research projects and that if he/ she withdraws from the trial any samples that have been collected up to the date of his/her withdrawal will not be destroyed.

Name of patient: ____

________________________________ ___________________ ___________________________

Name of Personal Representative **Date** **Signature**

____

Relationship to patient

__________________________________ ___________________ ___________________________

Name of person taking consent **Date** **Signature**

You must have signed the Site Signature & Delegation Log

Original to be kept in the Investigator Site File, 1 copy in hospital notes, 1 copy to the patient/ legal representative

For peer review only - http://bmjopen.bmj.com/site/about/guidelines.xhtml

<To be printed on hospital headed paper>

CATALYST- Early phase platform trial in (suspected or confirmed) COVID 19

Which treatment could lessen the severity of a (suspected or confirmed) COVID 19 infection when compared with usual care in an NHS setting?

Professional Legal Representative Informed Consent Form

Site: .... **Patient's Trial Number:** ☐☐☐

Investigator: .... **EudraCT:** 2020-001684-89

Please initial each box

1. I confirm that I been consulted about ....'s participant in this trial and have read and understand the Professional Legal Representative Information Sheet (version .... dated.......................) for the above trial and what it means to be a professional legal representative. I have had the opportunity to consider the information, ask questions and have had these answered satisfactorily. ☐

2. I understand that his/her participation is voluntary and that I am free to change my opinion on what he/she would have wished for at any time without giving any reason, and without his/her medical care or legal rights being affected. ☐

3. I understand that his/her direct informed consent will be obtained at the earliest opportunity. ☐

Original to be kept in the Investigator Site File, 1 copy in hospital notes, 1 copy to the patient/ legal representative,

For peer review only - http://bmjopen.bmj.com/site/about/guidelines.xhtml

4. I understand that his/her initials, date of birth and NHS Number will be given to the Trial Office based within the University of Birmingham (Birmingham, UK) when he/she is randomised to the trial.

5. I understand that relevant sections of his/her medical notes and data collected during the trial may be looked at by individuals from the Trial Office, regulatory authorities, the Sponsor (the University of Birmingham) and/or NHS bodies, where it is relevant to him/her taking part in this research.

6. I understand that anonymised data and samples from the trial may be provided to third parties (e.g. laboratories, pharmaceutical companies, other academic institutions or relevant UK government departments) for research, safety monitoring or licensing purposes, where applicable. I understand that this may involve sending data outside of the United Kingdom to a European country or the United States of America and that the patients name and any identifying details will NOT be given to these third parties, instead he/ she will be identified by his/ her unique Trial Number.

7. I understand that if he/she withdraws or is withdrawn from the trial, any samples and data that have been collected up to the date of withdrawal will be analysed and used as part of the research.

8. I agree to the patients GP being informed of his/ her participation in this trial.

9. In my opinion, the patient would have no objections to taking part in the above trial

OPTIONAL

10. I agree to the collection and storage of research samples for analysis as part of the CATALYST trial and for use in other ethically approved laboratory projects, which may include genetic analysis of these

For peer review only - http://bmjopen.bmj.com/site/about/guidelines.xhtml

samples. I understand that the patient will not be personally informed of the results of these additional research projects and that if he/ she withdraws from the trial any samples that have been collected up to the date of his/her withdrawal will not be destroyed.

____

Name of patient:

________________________________	___________________	___________________________
**Name of Professional Legal Representative**	**Date**	**Signature**
__________________________________	___________________	___________________________
**Name of person taking consent**	**Date**	**Signature**

You must have signed the Site Signature & Delegation Log

For peer review only

Original to be kept in the Investigator Site File, 1 copy in hospital notes, 1 copy to the patient/ legal representative,

For peer review only - http://bmjopen.bmj.com/site/about/guidelines.xhtml

Appendix 6 – CATALYST schedule of events for intervention arms

Arm 2: Gemtuzumab Ozogamicin (Mylotarg):

	Baseline	Day 1	Day 2	Day 3	Day 4	Day 5 (+24 hrs)	Day 6	Day 7	Day 8	Day 9	Day 10 (+24 hrs)	Day 11	Day 12	Day 13	Day 14	Day 15 – Day 27	Day 28⌂
IMP pre-medications #		x				x					x						
IMP Administration – Mylotarg		x				x					x						
Liver function test π		x				x					x						
Vital signs (heart rate, blood pressure, temperature) ~		x				x					x						

⌂ Information on Serious Adverse Events (SAEs) will be collected until 28 days after the last IMP administration, which may be after this time point.

Dexamethasone (9.9mg), antihistamine (chlorpheniramine 4-8mg PO or 10mg IV), paracetamol (1g PO or IV) to be given one hour prior to administration.

π Liver function test must be obtained (within the last 24hrs) and REVIEWED prior to IMP administration.

~ Vital signs must be monitored during the infusion and the patient observed for 4 hours after the infusion has ended.

Arm 3: Namilumab

	Baseline	Day 1	Day 2	Day 3	Day 4	Day 5	Day 6	Day 7	Day 8	Day 9	Day 10	Day 11	Day 12	Day 13	Day 14	Day 15 – Day 27	Day 28⌂
IMP administration – Namilumab		x															
Vital signs (heart rate, blood pressure, temperature) ~		x															

⌂ Information on Serious Adverse Events (SAEs) will be collected until 28 days after the last IMP administration, which may be after this time point.

~ Vital signs must be monitored during the infusion and the patient observed for 1 hour after the infusion has ended

For peer review only

For peer review only - http://bmjopen.bmj.com/site/about/guidelines.xhtml

Arm 4: Infliximab (Remsima)

	Baseline	Day 1	Day 2	Day 3	Day 4	Day 5	Day 6	Day 7	Day 8	Day 9	Day 10	Day 11	Day 12	Day 13	Day 14	Day 15 – Day 27	Day 28⌂
IMP pre-medications #		X															
IMP Administration - Infliximab		X															
Vital signs (heart rate, blood pressure, temperature) ~		X															

⌂ Information on Serious Adverse Events (SAEs) will be collected until 28 days after the last IMP administration, which may be after this time point.

PRN only - antihistamine (chlorpheniramine 4-8mg PO or 10mg IV), paracetamol (1g PO or IV) to be given one hour prior to administration at local centre discretion.

~ Vital signs must be monitored during the infusion and the patient observed for 2 hours after the infusion has ended.

For peer review only

For peer review only - http://bmjopen.bmj.com/site/about/guidelines.xhtml

Supplementary Appendix 7 – Statistical considerations

The simulations and tables below demonstrate the operating characteristics of a trial design with the chosen decision criteria, based on a simpler analysis of the area under the curve for sequential CRP data, with effect sizes informed from a dataset from 1026 hospitalised COVID-19 patients at Queen Elizabeth Hospital, Birmingham. In our simulations, we compared a traditional fixed trial design recruiting 120 patients with candidate adaptive designs. We present basic operating characteristics for the fixed design (Table 6A) and the chosen adaptive design (Table 6B). We studied six scenarios of treatment effect, and estimated, through simulation, the probability of a trial stopping early for "success" or "futility," and ultimately concluding success. Simulations were performed in Fixed and Adaptive Clinical Trial Simulator (FACTS) software using default non-informative priors.

Table 6A. Operating characteristics for a fixed trial design of 120 patients.

Scenario	Probability stopping early for success	Probability stopping early for futility	Overall probability of success	Mean number of patients
Null	0	0	0.101	120
A	0	0	0.537	120
B	0	0	0.926	120
C	0	0	0.997	120
D	0	0	0.008	120
E	0	0	0	120

Scenarios A, B, and C are beneficial effects of the intervention with (true) treatment effects of 0.25, 0.5 and 0.75 standard deviations, "null" is zero treatment effect and D and E are harmful effects of 0.25 and 0.5 standard deviations. "success" and "futility" are defined as above.

Table 6B. Operating characteristics for an adaptive design with interim analyses at 40 and 80 patients.

Scenario	Probability stopping early for success	Probability stopping early for futility	Overall probability of success	Mean number of patients
Null	0.148	0.624	0.176	66
A	0.455	0.281	0.559	70
B	0.798	0.089	0.890	59
C	0.965	0.012	0.985	48
D	0.03	0.901	0.031	52
E	0.003	0.986	0.003	43

The adaptive design achieves similar probabilities of success in scenarios where the treatment effect is truly beneficial (A, B and C), and increases the probability of success only slightly if the intervention is harmful (D and E). There is some increase in the probability of success if the treatment effect is zero (Type I error) but this is offset by the very substantial reductions in the numbers of patients needed in all scenarios. Moreover, Type I error is not a serious problem as all interventions would be evaluated further in phase III trials.

For peer review only - http://bmjopen.bmj.com/site/about/guidelines.xhtml

Statistical Analysis Plan

A randomised phase II proof of principle multi-arm multi-stage trial designed to guide the selection of interventions for phase III trials in hospitalised patients with COVID-19 infection.

Version: 2.0
March 17, 2021

Sponsor(s): University of Birmingham
EudraCT Number: 2020-001684-89
Sponsor Reference Number: RG_20-030

Author(s):

Name: Mr Daniel Slade
Trial role: Statistical Lead

Name: Miss Charlotte Gaskell
Trial role: Trial Statistician

Name: Professor Simon Gates
Trial role: Statistical Lead & Co-Investigator

Reviewed & Approved by:

Name: Dr. Benjamin Fisher
Trial role: Acting Chief Investigator

Cancer Research UK Clinical Trials Unit
@CRCTU

For peer review only - http://bmjopen.bmj.com/site/about/guidelines.xhtml

Key personnel involved in the Statistical Analysis Plan:

Name	Trial role
Mr Daniel Slade	Statistical Lead
Miss Charlotte Gaskell	Trial Statistician
Dr. Tonny Venith	Chief Investigator
Dr. Benjamin Fisher	Deputy Chief Investigator
Professor. Simon Gates	Statistical Lead & Co-Investigator

Document Control Sheet:

Statistical Analysis Plan version:	Reason for update:
v1.0	Initial Version
v2.0	Incorporation of joint-modelling and AUC approach for the primary analysis, complimentary analyses to attempt to take account of censoring. Addition of ITT analysis for secondary endpoints and MITT definition for secondary endpoints. Modification of primary analysis to state inference will be based on only the interaction term of the model. Specification of subgroup analyses based on disease severity, a request of the DMC.

For peer review only

For peer review only - http://bmjopen.bmj.com/site/about/guidelines.xhtml

Contents

1 INTRODUCTION 4
1.1 Purpose of the Statistical Analysis Plan 4
1.2 Summary of the Trial 4

2 TIMING AND REPORTING OF INTERIM AND FINAL ANALYSES 6

3 RECRUITMENT AND RANDOMISATION 6
3.1 Recruitment 6
3.2 Randomisation 6
3.3 Ineligible Patients 6

4 DATA QUALITY 6
4.1 Data Quality: CRFs 6
4.2 Return Rates: CRFs 6

5 TRIAL POPULATION 7
5.1 Patient Characteristics 7
5.2 Definition of Populations for Analysis 7

6 TREATMENT RECEIVED 7

7 SAFETY ANALYSIS 7

8 ANALYSIS 7
8.1 Analysis of Primary Outcome Measure 8
8.2 Analysis for Secondary Outcome Measures 8
8.3 Subgroup Analysis 9

9 SAMPLE SIZE 9

10 STATISTICAL SOFTWARE 10

11 STORAGE AND ARCHIVING 10

For peer review only - http://bmjopen.bmj.com/site/about/guidelines.xhtml

1 INTRODUCTION

1.1 Purpose of the Statistical Analysis Plan

This Statistical Analysis Plan (SAP) provides guidelines for the analysis and presentation of results for the Catalyst trial. This plan, along with all other documents relating to the analysis of this trial, will be stored in the 'Statistical Documentation' section of the Trial Master File. The statistical analysis will be carried out by the trial Statisticians.

1.2 Summary of the Trial

Trial Design
Catalyst is a rapid, open-label, phase II, multi-arm, multi-stage trial permitting an efficient evaluation of the potential efficacy of these targeted drugs which can then be considered for larger-scale testing by one of the current national platform trials.

Objectives
Primary Objectives

- To investigate whether candidate treatments demonstrate evidence of greater attenuation of inflammation as defined by an improvement in C-reactive protein (CRP) concentrations compared with usual care in COVID-19 patients.
- To recommend drugs that should be evaluated further in one of the phase III trials.

Outcome Measures
Primary Outcome Measures

- C-reactive protein measured over time up to day 14 for each patient.

Secondary Outcome Measures

- World Health Organisation (WHO) Clinical Progression improvement Scale (1-10 scale; for the purposes of this trial level 0, no viral RNA detected, will not be assessed)
- The ratio of the oxygen saturation to fractional inspired oxygen concentration (SpO2/FiO2), measured from randomisation to day 14, hospital discharge or death. SpO2 and FiO2 are measured as part of routine clinical care
- Respiratory rate
- Body temperature
- NEWS-2 score
- Length of hospital stay
- Hospital survival status at day 28 / hospital free days
- Proportion of patients discharged at day 28
- Destination of discharge
- Lymphocyte and Neutrophil counts and ratios
- Ferritin, D-Dimer and LDH
- Adverse events (AEs) and Serious Adverse Events (SAEs) as recorded by Common Terminology Criteria for Adverse Events (CTCAE), version 4.03 of grade ≥ 3 with interest in veno-occlusive disease (VOD), secondary infection and allergic reaction
- Overall Survival

Exploratory Outcome Measures

For peer review only - http://bmjopen.bmj.com/site/about/guidelines.xhtml

- Blood inflammatory mediators, biomarkers, transciptome and cellular immunology in relation to COVID-19 infection
- Viral load
- Host DNA assessed at baseline to assess for predictors of disease severity and drug response
- blood biomarkers of aveolar epithelial cell damage to include surfactant D and RAGE

Patient Population
This trial seeks to recruit hospitalised patients with COVID-19 who are hypoxic, admitted to either a hospital ward or ICU, and are at risk of deterioration.

Sample Size
A total of up to 60 patients per treatment arm will be recruited.

For peer review only

For peer review only - http://bmjopen.bmj.com/site/about/guidelines.xhtml

2 TIMING AND REPORTING OF INTERIM AND FINAL ANALYSES

There two planned interim analyses for the primary endpoint at n=20 and n=40 per arm respectively. Data analyses pertaining to trial conduct, data quality and patient safety will be supplied in confidence to an independent DMC throughout the period the trial is running. The DMC shall review the available data on a proposed 3 monthly basis.

The final analyses for the trial will be conducted once the end of trial has been reached. The final analyses will incorporate the primary, secondary and all exploratory outcomes as detailed in this analysis plan. The end of trial is defined as 6 months after the last data capture.

3 RECRUITMENT AND RANDOMISATION

3.1 Recruitment

At the point of analysis the following data will be reported:

- Date of the database snapshot used for recruitment analysis
- Total number of patients who have been recruited into the trial and randomised to each treatment arm
- Recruitment over time (monthly and cumulative)
- Recruitment by site

3.2 Randomisation

Patients will be randomised 1:1 between Usual Care (Control Arm) and interventional arms using the minimisation procedure described by Pocock and Simon, with a single stratification variable with two levels; Care status: 'On ward' or 'ICU'. Patients will be randomised into either a control group or to receive interventional treatments that are available at their site.

3.3 Ineligible Patients

Ineligible patients are defined as those registered patients who are subsequently found to not meet the eligibility criteria of the trial after being recruited. The proportion of ineligible patients and reasons for their ineligibility will be reported for each treatment arm. In addition the number of patients who were screened in total will be reported along with the number of patients not recruited to the trial and their associated reasons e.g. ineligible.

4 DATA QUALITY

4.1 Data Quality: CRFs

Patient data is collected using case report forms (CRFs) and electronic case report forms (eCRFs). Data collected in this way will be stored on a trial database. The trial database will be checked for missing data and any discrepancies at least annually but prior to any analysis as according to the trial specific data validation plan, which will be developed by both the trial statisticians and the trial coordinator.

4.2 Return Rates: CRFs

The proportion of returned CRFs compared to those that were expected will be reported for each case report form.

For peer review only

For peer review only - http://bmjopen.bmj.com/site/about/guidelines.xhtml

5 TRIAL POPULATION

5.1 Patient Characteristics

A summary of patient characteristics will be reported. Descriptive statistics will be provided in the summary including counts and percentages for categorical data items and mean (sd), median and ranges for continuous data items.

5.2 Definition of Populations for Analysis

Safety Population - Safety population will include all patients who receive any trial treatment. For interventional arms this requires the patient to have received some IMP.

MITT Population - The Modified Intention-To-Treat population for the primary analyses will include all patients who receive any trial treatment and who have a baseline CRP measurement and at least one further CRP measurement post baseline. For the secondary endpoints, this includes all patients who receive any trial treatment and have available data for the respective outcome measure.

ITT Population - This includes all randomised patients in their treatment arms, that have available data for the respective outcome measure.

6 TREATMENT RECEIVED

For each treatment arm, the proportion of participants who received treatment as per protocol will be reported. The proportion of participants who discontinued treatment early will also be reported along with a tabulation of the reasons. Summary statistics for all participants on treatment arms will be reported e.g. median/mean time on treatment, these statistics will be tailored for the specific arms as naturally the treatments may widely differ and thus different summary measures will be relevant.

7 SAFETY ANALYSIS

The number of serious adverse events (including SARs and SUSUARs), and the number of treatment-related deaths will be reported for each treatment arm. The reporting period for Adverse Events/Serious Adverse Events (SAE's) will commence from the date of consent. Safety will be assessed by looking at adverse events (CTCAE).

The following details will be reported for each treatment arm for all patients who are part of the safety population:

- Adverse events at baseline, summarised by event and number of patients experiencing such events.
- Max grade experienced for all patients.
- A summary of number of events and patients for all toxicities by event and grade.
- The number of events and patients for all grades of toxicities.
- All serious adverse events will be reported, details to be presented include but are not limited to; admitting event, other events, reason for SAE, outcome, sequel and relatedness.

8 ANALYSIS

For all analyses data will be analysed for each intervention against the control group, including in each analysis only those participants who were eligible for the those treatment arms at the point of randomisation. The primary analysis will be conducted on the MITT population and all secondary analyses will be conducted on both the MITT population and ITT unless otherwise specified.

New intervention arms may be added as new interventions become available. All comparisons will be performed temporally with regards to control arm data.

For peer review only - http://bmjopen.bmj.com/site/about/guidelines.xhtml

8.1 Analysis of Primary Outcome Measure

The CRP data will be modelled using Bayesian multi-level models that allow for nesting of the repeated measures data within patient, and allowing for non-linear responses. This approach will facilitate an assessment of the effects of the treatments on the CRP. Specifically, posterior probabilities for the treatment/time interaction term will be used to conduct decision making. Care status as a randomisation stratification factor will be incorporated accordingly into the model structure along with age as a known prognostic indicator.

At the specified decision points, with interim analyses at n=20 and n=40 and a final analysis at n=60 per arm, the CRP data will be considered in the context of the emerging safety data to make a recommendation as outlined below:

a) If there is strong evidence of an additional anti-inflammatory effect (CRP) and a satisfactory safety profile consider progression to clinical endpoint evaluation whether in this trial or in another one

b) Terminate arm and do not proceed (based on lack of evidence of an additional biological effect or of an unfavourable safety signal)

We will define that 'strong evidence' or 'success' will be if there is an 90% probability that the intervention arm is better than usual care in reducing CRP as seen by the treatment/time interaction covariate. 'lack of evidence' or 'futility' is defined as less than 50% probability of the intervention being better than usual care. However, given the large number of agents being investigated in various phase II trials, the size of effect and the totality of data will be reviewed before recommending adoption by a phase III platform.

In addition to the above analysis we will analyse the data using two further approaches, namely, modelling AUC and an additional joint-modelling approach for CRP and discharge/death, this is to ascertain if censoring events for CRP; discharge/death, have had any impact on inference and if so to model accordingly.

8.2 Analysis for Secondary Outcome Measures

Outcome measures

- World Health Organisation (WHO) Clinical Progression improvement Scale
  - Time to improvement, measured from the date of randomisation, an event here is defined as at least a one-point improvement on the Time to Clinical Improvement Scale. A Kaplan-Meier plot will be produced for each treatment and control arm comparison, estimates of median time to improvement will be reported along with associated confidence intervals (where they can be estimated). In addition to the one-point improvement an additional analysis utilising a two-point improvement will be conducted, to be comparable with other studies.
  - Patients' scores on the Clinical Improvement Scale for each day will be displayed graphically, and modelled using Bayesian longitudinal ordinal regression, as described by Harrell (`http://hbiostat.org/proj/covid19/bayesplan.html`).
- The ratio of the oxygen saturation to fractional inspired oxygen concentration (SpO2/FiO2) will be presented graphically over time.
- Length of hospital stay will be summarised via descriptive statistics, stratified by treatment group. Reasons for such lengths of stay will be reported and summarised accordingly.
- Respiratory rate, body temperature and NEWS-2, will be plotted over time and summarised through descriptive statistics. These measures may also be modelled over time using multilevel modelling. Exploratory data analysis will drive model formulation, assumptions will be tested accordingly. All modelling will be exploratory in nature.
- The proportion of patients discharged at day 28 along with destination of discharge will be presented accordingly.
- Hospital survival status at 28 days will be reported as a tabulation of the proportion of patients who have died, been discharged or are still in hospital by day 28. Hospital-free days will be summarised through descriptive statistics, patients still in hospital or who have died will be incorporated having 0 hospital-free days.

For peer review only - http://bmjopen.bmj.com/site/about/guidelines.xhtml

- Lymphocyte, neutrophil and full blood counts with lymphocyte: neutrophil ratios and ferritin, D-Dimer and Triglycerides LDH values will be plotted over time and summarised through descriptive statistics. These measures may also be modelled over time using multilevel modelling. Exploratory data analysis will drive model formulation, assumptions will be tested accordingly. All modelling will be exploratory in nature.
- AEs and SAEs will be analysed as per section 7
- Overall Survival - Measured from the date of registration, an event here is defined as death. Patients are followed up until they have either died or are censored at date last seen. A Kaplan-Meier plot will be produced for each comparison, estimates of median survival will be reported along with associated confidence intervals (where they can be estimated)

8.3 Subgroup Analysis

Exploratory subgroup analyses will be conducted to attempt to ascertain the effect of disease severity on outcomes. The subgroups of 'non-severe disease' and 'severe disease' are defined as those that have a baseline WHO score of < 6 and ≥ 6 respectively. Other exploratory subgroup analyses may be conducted based on known prognostic indicators e.g. age group.

9 SAMPLE SIZE

The tables below demonstrate the operating characteristics of a trial design with the chosen decision criteria, based on a simpler analysis of area under the curve for sequential CRP data, with effect sizes informed from a dataset from 1026 hospitalised COVID-19 patients at Queen Elizabeth Hospital, Birmingham.

It is anticipated that our proposed hierarchical analysis will have superior operating characteristics. In our simulations, we compared a traditional fixed trial design recruiting 120 patients with candidate adaptive designs. We present basic operating characteristics for the fixed design (Table 1) and the chosen adaptive design (Table 2). We studied six scenarios of treatment effect, and estimated, through simulation, the probability of a trial stopping early for "success" or "fultility," and ultimately concluding success. Scenarios A, B, and C are beneficial effects of the intervention with (true) treatment effects of 0.25, 0.5 and 0.75 standard deviations, "null" is zero treatment effect and D and E are harmful effects of 0.25 and 0.5 standard deviations. "success" and "futility" are defined as above.

Table 1: Operating characteristics for a fixed trial design of 120 patients

Scenario	Probability stopping early for success	Probability stopping early for futility	Overall probability of success	Mean number of patients
Null	0	0	0.101	120
A	0	0	0.537	120
B	0	0	0.926	120
C	0	0	0.997	120
D	0	0	0.008	120
E	0	0	0	120

The adaptive design achieves similar probabilities of success in scenarios where the treatment effect is truly beneficial (A, B and C), and increases the probability of success only slightly if the intervention is harmful (D and E). There is some increase in the probability of success if the treatment effect is zero (Type I error) but this is offset by the very substantial reducitions in the numbers of patients needed in all scenarios. Moreover, Type I error is not seen as a serious problem as all interventions would be evaluated further in Phase 3 trials.

For peer review only - http://bmjopen.bmj.com/site/about/guidelines.xhtml

Table 2: operating characteristics for an adaptive design with interim analyses at 40 and 80 patients

Scenario	Probability stopping early for success	Probability stopping early for futility	Overall probability of success	Mean number of patients
Null	0.140	0.607	0.143	70
A	0.471	0.254	0.573	74
B	0.847	0.062	0.910	61
C	0.974	0.010	0.989	51
D	0.030	0.918	0.031	54
E	0.003	0.985	0.003	47

10 STATISTICAL SOFTWARE

Statistical analyses will be carried out using relevant statistical software; SAS , Stata or R respectively. Version numbers and session details will be stated and logged with any analysis.

11 STORAGE AND ARCHIVING

Catalyst files are stored in a restricted access directory on a secure server and will be saved for archive purposes according to CRCTU policy and procedure.

For peer review only

For peer review only - http://bmjopen.bmj.com/site/about/guidelines.xhtml

Supplementary Appendix 9 – Adverse event definitions

Adverse Event

Any untoward medical occurrence in a patient or clinical trial subject administered a medicinal product and which does not necessarily have a causal relationship with this treatment.

Comment:

An AE can therefore be any unfavourable and unintended sign (including abnormal laboratory findings), symptom or disease temporally associated with the use of an investigational medicinal product, whether or not related to the investigational medicinal product.

Adverse Reaction

All untoward and unintended responses to an IMP related to any dose administered.

Comment:

An AE judged by either the reporting Investigator or Sponsor as having causal relationship to the IMP qualifies as an AR. The expression reasonable causal relationship means to convey in general that there is evidence or argument to suggest a causal relationship.

Serious Adverse Event

Any untoward medical occurrence or effect that at any dose:

Results in death

Is life threatening*

Requires hospitalisation** or prolongation of existing inpatients' hospitalisation

Results in persistent or significant disability or incapacity

Is a congenital anomaly/birth defect

Or is otherwise considered medically significant by the investigator ***

Comments:

The term severe is often used to describe the intensity (severity) of a specific event. This is not the same as serious, which is based on patients/event outcome or action criteria.

* Life threatening in the definition of an SAE refers to an event in which the patient was at risk of death at the time of the event; it does not refer to an event that hypothetically might have caused death if it were more severe.

** hospitalisation is defined as an unplanned, formal inpatient admission, even if the hospitalisation is a precautionary measure for continued observation. Thus, hospitalisation for protocol treatment (e.g. line insertion), elective procedures (unless brought forward because of worsening symptoms) or for social reasons (e.g. respite care) are not regarded as an SAE.

*** Medical judgment should be exercised in deciding whether an AE is serious in other situations. Important AEs that are not immediately life threatening or do not result in death or hospitalisation but may jeopardise the subject or may require intervention to prevent one of the other outcomes listed in the definition above, should be considered serious.

Serious Adverse Reaction

An Adverse Reaction which also meets the definition of a Serious Adverse Event.

Suspected Unexpected Serious Adverse Reaction

A SAR that is unexpected i.e. the nature, or severity of the event is not consistent with the applicable product information.

For peer review only - http://bmjopen.bmj.com/site/about/guidelines.xhtml

A SUSAR should meet the definition of an AR, UAR and SAR.

Unexpected Adverse Reaction

An AR, the nature or severity of which is not consistent with the applicable product information (e.g. Investigator Brochure for an unapproved IMP or (compendium of) Summary of Product Characteristics (SPC) for a licensed product).

When the outcome of an AR is not consistent with the applicable product information the AR should be considered unexpected.

For peer review only

For peer review only - http://bmjopen.bmj.com/site/about/guidelines.xhtml

SPIRIT

STANDARD PROTOCOL ITEMS: RECOMMENDATIONS FOR INTERVENTIONAL TRIALS

SPIRIT 2013 Checklist: Recommended items to address in a clinical trial protocol and related documents*

Section/item	Item No	Description
**Administrative information**		
Title	1	Descriptive title identifying the study design, population, interventions, and, if applicable, trial acronym<br><br>Page 1; lines 1-3
Trial registration	2a	Trial identifier and registry name. If not yet registered, name of intended registry<br><br>Page 2; lines 55-56
	2b	All items from the World Health Organization Trial Registration Data Set<br><br>Appendix 2
Protocol version	3	Date and version identifier<br><br>Page 2; lines 50-51
Funding	4	Sources and types of financial, material, and other support<br><br>Page 10; lines 399-410
Roles and responsibilities	5a	Names, affiliations, and roles of protocol contributors<br><br>Names and affiliations on page 1; lines 4-31<br>Names and role on page 10; lines 394-396
	5b	Name and contact information for the trial sponsor<br><br>Page 7; lines 281-282
	5c	Role of study sponsor and funders, if any, in study design; collection, management, analysis, and interpretation of data; writing of the report; and the decision to submit the report for publication, including whether they will have ultimate authority over any of these activities<br><br>Page 10; lines 413-415
	5d	Composition, roles, and responsibilities of the coordinating centre, steering committee, endpoint adjudication committee, data management team, and other individuals or groups overseeing the trial, if applicable (see Item 21a for data monitoring committee)

For peer review only

		Page 8; lines 291-309
**Introduction**		
Background and rationale	6a	Description of research question and justification for undertaking the trial, including summary of relevant studies (published and unpublished) examining benefits and harms for each intervention<br>Page 3; lines 69-92
	6b	Explanation for choice of comparators<br>Page 5; lines 157-159
Objectives	7	Specific objectives or hypotheses<br>Page 3; lines 89-91
Trial design	8	Description of trial design including type of trial (eg, parallel group, crossover, factorial, single group), allocation ratio, and framework (eg, superiority, equivalence, noninferiority, exploratory)<br>Page 3; lines 96-105
**Methods: Participants, interventions, and outcomes**		
Study setting	9	Description of study settings (eg, community clinic, academic hospital) and list of countries where data will be collected. Reference to where list of study sites can be obtained<br>Page 4; line 122 and Appendix 1
Eligibility criteria	10	Inclusion and exclusion criteria for participants. If applicable, eligibility criteria for study centres and individuals who will perform the interventions (eg, surgeons, psychotherapists)<br>Page 4; lines 122-136
Interventions	11a	Interventions for each group with sufficient detail to allow replication, including how and when they will be administered<br>Page 5; lines 157-178
	11b	Criteria for discontinuing or modifying allocated interventions for a given trial participant (eg, drug dose change in response to harms, participant request, or improving/worsening disease)<br>n/a
	11c	Strategies to improve adherence to intervention protocols, and any procedures for monitoring adherence (eg, drug tablet return, laboratory tests)<br>n/a

For peer review only - http://bmjopen.bmj.com/site/about/guidelines.xhtml

	11d	Relevant concomitant care and interventions that are permitted or prohibited during the trial<br><br>Page 5; lines 181-18
Outcomes	12	Primary, secondary, and other outcomes, including the specific measurement variable (eg, systolic blood pressure), analysis metric (eg, change from baseline, final value, time to event), method of aggregation (eg, median, proportion), and time point for each outcome. Explanation of the clinical relevance of chosen efficacy and harm outcomes is strongly recommended<br><br>Page 5; lines 189-214
Participant timeline	13	Time schedule of enrolment, interventions (including any run-ins and washouts), assessments, and visits for participants. A schematic diagram is highly recommended (see Figure)<br><br>Table 2 and Appendix 9
Sample size	14	Estimated number of participants needed to achieve study objectives and how it was determined, including clinical and statistical assumptions supporting any sample size calculations<br><br>Page 6; lines 217-239
Recruitment	15	Strategies for achieving adequate participant enrolment to reach target sample size<br><br>n/a

Methods: Assignment of interventions (for controlled trials)

Allocation:

Sequence generation	16a	Method of generating the allocation sequence (eg, computer-generated random numbers), and list of any factors for stratification. To reduce predictability of a random sequence, details of any planned restriction (eg, blocking) should be provided in a separate document that is unavailable to those who enrol participants or assign interventions<br><br>Page 3; lines 102-105
Allocation concealment mechanism	16b	Mechanism of implementing the allocation sequence (eg, central telephone; sequentially numbered, opaque, sealed envelopes), describing any steps to conceal the sequence until interventions are assigned<br><br>n/a
Implementation	16c	Who will generate the allocation sequence, who will enrol participants, and who will assign participants to interventions

For peer review only - http://bmjopen.bmj.com/site/about/guidelines.xhtml

		Page 4; line 152-154
Blinding (masking)	17a	Who will be blinded after assignment to interventions (eg, trial participants, care providers, outcome assessors, data analysts), and how<br><br>n/a
	17b	If blinded, circumstances under which unblinding is permissible, and procedure for revealing a participant's allocated intervention during the trial<br><br>n/a
**Methods: Data collection, management, and analysis**		
Data collection methods	18a	Plans for assessment and collection of outcome, baseline, and other trial data, including any related processes to promote data quality (eg, duplicate measurements, training of assessors) and a description of study instruments (eg, questionnaires, laboratory tests) along with their reliability and validity, if known. Reference to where data collection forms can be found, if not in the protocol<br><br>Page 7; lines 268-273
	18b	Plans to promote participant retention and complete follow-up, including list of any outcome data to be collected for participants who discontinue or deviate from intervention protocols<br><br>Page 6; lines 204-207
Data management	19	Plans for data entry, coding, security, and storage, including any related processes to promote data quality (eg, double data entry; range checks for data values). Reference to where details of data management procedures can be found, if not in the protocol<br><br>Page 7; lines 265-269
Statistical methods	20a	Statistical methods for analysing primary and secondary outcomes. Reference to where other details of the statistical analysis plan can be found, if not in the protocol<br><br>Page 6; lines 217-243 and Appendices 6 and 7
	20b	Methods for any additional analyses (eg, subgroup and adjusted analyses)<br><br>Page 6; lines 243-245
	20c	Definition of analysis population relating to protocol non-adherence (eg, as randomised analysis), and any statistical methods to handle missing data (eg, multiple imputation)<br><br>Page 7; lines 247-251

For peer review only - http://bmjopen.bmj.com/site/about/guidelines.xhtml

Methods: Monitoring

Data monitoring	21a	Composition of data monitoring committee (DMC); summary of its role and reporting structure; statement of whether it is independent from the sponsor and competing interests; and reference to where further details about its charter can be found, if not in the protocol. Alternatively, an explanation of why a DMC is not needed<br>Page 8; lines 298-304
	21b	Description of any interim analyses and stopping guidelines, including who will have access to these interim results and make the final decision to terminate the trial<br>Page 6; lines 224-230
Harms	22	Plans for collecting, assessing, reporting, and managing solicited and spontaneously reported adverse events and other unintended effects of trial interventions or trial conduct<br>Page 7; lines 254-265
Auditing	23	Frequency and procedures for auditing trial conduct, if any, and whether the process will be independent from investigators and the sponsor<br>Page 7; lines 273-277

Ethics and dissemination

Research ethics approval	24	Plans for seeking research ethics committee/institutional review board (REC/IRB) approval<br>Page 10; lines 383-387
Protocol amendments	25	Plans for communicating important protocol modifications (eg, changes to eligibility criteria, outcomes, analyses) to relevant parties (eg, investigators, REC/IRBs, trial participants, trial registries, journals, regulators)<br>Appendix 3
Consent or assent	26a	Who will obtain informed consent or assent from potential trial participants or authorised surrogates, and how (see Item 32)<br>Page 4; line 139-150
	26b	Additional consent provisions for collection and use of participant data and biological specimens in ancillary studies, if applicable<br>n/a

For peer review only - http://bmjopen.bmj.com/site/about/guidelines.xhtml

Confidentiality	27	How personal information about potential and enrolled participants will be collected, shared, and maintained in order to protect confidentiality before, during, and after the trial<br><br>Page 8; lines 312-314
Declaration of interests	28	Financial and other competing interests for principal investigators for the overall trial and each study site<br><br>Page 10; lines 418-420
Access to data	29	Statement of who will have access to the final trial dataset, and disclosure of contractual agreements that limit such access for investigators<br><br>Page 7; lines 277-278
Ancillary and post-trial care	30	Provisions, if any, for ancillary and post-trial care, and for compensation to those who suffer harm from trial participation<br><br>n/a
Dissemination policy	31a	Plans for investigators and sponsor to communicate trial results to participants, healthcare professionals, the public, and other relevant groups (eg, via publication, reporting in results databases, or other data sharing arrangements), including any publication restrictions<br><br>Page 10; lines 388-390
	31b	Authorship eligibility guidelines and any intended use of professional writers<br><br>Page 10; lines 390-391
	31c	Plans, if any, for granting public access to the full protocol, participant-level dataset, and statistical code<br><br>n/a
**Appendices**		
Informed consent materials	32	Model consent form and other related documentation given to participants and authorised surrogates<br><br>Appendices 4 and 5
Biological specimens	33	Plans for collection, laboratory evaluation, and storage of biological specimens for genetic or molecular analysis in the current trial and for future use in ancillary studies, if applicable<br><br>n/a

*It is strongly recommended that this checklist be read in conjunction with the SPIRIT 2013 Explanation & Elaboration for important clarification on the items. Amendments to the protocol should be tracked and dated. The SPIRIT checklist is copyrighted by the SPIRIT

For peer review only

Group under the Creative Commons "Attribution-NonCommercial-NoDerivs 3.0 Unported" license.

For peer review only
